# Efficacy of Albendazole and Mebendazole Against Soil Transmitted Infections among Pre-School and School Age Children: A Systematic Review and Meta-Analysis

**DOI:** 10.1007/s44197-024-00231-7

**Published:** 2024-05-02

**Authors:** Temesgen Bekele, Lata Lachisa, Arega Tsegaye, Ketema Bacha, Tsige Ketema

**Affiliations:** College of Natural Sciences, Department of Biology, Jimma University, Jimma, Ethiopia

**Keywords:** Albendazole, Ascaris, Hookworms, Mebendazole, Soil transmitted helminthes, Trichuris

## Abstract

**Background:**

Soil-transmitted helminthic (STH) infections are the leading cause of stunting among children. To lessen the burden, the World Health Organization (WHO) recommended a periodic deworming program through the use of single-dose therapy in the endemic regions. Therefore, the purpose of this study was to synthesize evidence about the efficacy of anthelminthic drugs against STH infections among preschool and school-age children.

**Methods:**

The Preferred Reposting Items for Systematic Reviews and Meta-Analyses (PRISMA) criteria were followed in this study. Relevant electronic databases, including PubMed, Scopus, Embase, DOAJ, Science Direct, the WHO Clinical Trials.gov library, Google Scholar, and AJOL databases, were searched for relevant publications. Randomized controlled trials (RCTs) and non-randomized interventional studies focused on the efficacy of albendazole and mebendazole against STHs in children were included in the study. Review Manager was used to analyze the data. A random effects model was used to obtain the pooled estimated efficacy. To evaluate heterogeneity, the *I*^*2*^ test and Cochrane Q (χ^2^) were employed. The risk of publication bias was investigated using Egger’s test and the funnel plot. The protocol of this review was registered at the PROSPERO international prospective register of systematic reviews (CRD42023401196).

**Results:**

Of the 69 publications selected for the systematic review, 66 with complete data were included in the meta-analysis. Single doses of albendazole and mebendazole have shown satisfactory efficacy [egg reduction rate (ERR)] against *Ascaris lumbricoides* [95.54% (95% CI: 88.75–102.34%) and 98.69% (95% CI: 97.68–99.65%), respectively. The effectiveness of these two drugs against *Trichuris trichiura* and hookworms was comparatively low (< 80% ERR), except for albendazole, which showed high ERRs [93.44% (95%CI: 92.39–94.49%)] against hookworms. The cure rate (CR) of albendazole against *T. trichiura*, *A. lumbricoides*, and hookworms were 50.8%, 91.3%, and 78.32%, respectively. Likewise, mebendazole showed CRs of 48.15%, 92.8%, and 49.32% against *T. trichiura*, *A. lumbricoides*, and hookworms, respectively. Subgroups such as studies conducted after 2000, diagnostic type (McMaster), and longer follow-up weeks significantly reduced the efficacy of the two drugs against *T. trichura*. While the combination of albendazole or mebendazole with other drugs and RCT showed significantly improved efficacy against *T. trichura.* The count of eggs per gram of stool (EPG) was identified as one of the variables that negatively and significantly influenced the efficacy of albendazole or mebendazole against *A. lumbricoides*.

**Conclusion:**

Despite the wide range of ERRs and CR reported in the different articles included in this review, the pooled estimated efficacy of albendazole and mebendazole against STHs falls in the satisfactory category of WHO recommendations. Further evaluation of the combination of anthelminthic drugs as a preventive chemotherapy option and routine drug efficacy testing are necessary to prevent the emergence and widespread use of drug-resistant STHs.

**Supplementary Information:**

The online version contains supplementary material available at 10.1007/s44197-024-00231-7.

## Introduction

Over 4.5 billion individuals are at risk of contracting soil-transmitted helminthic (STH) infections, which are one of the neglected tropical diseases (NTDs) that affect an estimated 2 billion people worldwide [1]. These STH are particularly common in tropical and subtropical regions of the world, mainly affecting the most impoverished and marginalized populations that have limited access to hygienic conditions, clean water, and sanitation [2]. *Ascaris lumbricoides*, *T. trichiura*, and hookworms (*Ancylostoma duodenale* and *Necator americanus*) are the principal STH parasites [1]. Preschool aged children, school-age children, and women in their reproductive years, and adults working in high-risk activities such as mining, and farming are the most vulnerable populations [2].

Achieving and maintaining the elimination of STH morbidity in preschool- and school-aged children is one of the World Health Organization (WHO) global targets by 2030 [3]. The establishment of routine mass drug administration along with routine anti-helminthic treatment was the technique used to achieve this target [4]. The WHO stated that in 103 countries where STH is endemic, an estimated 267.5 million preschool-aged children and 568.8 million school-aged children need therapy [5]. Around 500 million children, 60% of them at risk, received preventive chemotherapy in endemic countries in 2021. Preventive chemotherapy’s main goal is to reduce morbidity in the risk population by lowering the frequency of infections with moderate- and heavy-intensity [6, 7]. To set and maintain public health safety beyond the original targets, the WHO recommended preventive chemotherapy, which is chiefly single-doses of albendazole (400 mg) and mebendazole (500 mg), which are efficient, affordable, and simple to administer by non-medical personnel [8, 9].

The WHO made recommendations during its review of the 2017 WHO Guideline of Preventive Chemotherapy to control STH infections in at-risk population groups, including monitoring anthelminthic efficacy of front-line treatments and the use of drug combinations to increase anthelminthic effectiveness and mitigate risks of developing drug resistance for both first-line and second-line treatments [9]. Using a range of study settings, treatment options, and follow-up days, many researchers conducted efficacy trials of these prophylactic chemotherapies and found differing cure rates (CRs) and egg reduction rates (ERRs) in different geographical regions. Therefore, this study was to design to answer á review question *‘what are the efficacy of albendazole and mebendazole against soil transmitted helminthic infections among pre-school and school age children?’*

## Methodology

### Research Design

The study was conducted according to Preferred Reporting Items for Systematic Reviews and Meta-Analyses (PRISMA) guidelines. Hence the review proposal was designed in a way that could help to develop a search strategy. The protocol was registered at PROSPERO International Prospective Register of systematic reviews, with ID: CRD42023401196 (available at: https://www.crd.york.ac.uk/prospero/display_record.php?ID=CRD42023401196) [10].

### Searches Methods

A comprehensive search of the literature was carried out using the following academic electronic databases: PubMed, Scopus, EMBASE, ScienceDirect, WHO Clinical Trial.gov. library, Directory of Open Access Journals (DOAJ), African Journals Online (AJOL), and Google Scholar. In addition, an effort was made to gather additional publications manually and to contact the original author to get further details and clarification. An in-depth search strategy using Medical subject heading (MeSH) terms and search strings used as follows; (“***Efficacy AND Albendazole AND Mebendazole AND soil-transmitted helminths OR STH AND children***”)] in titles or abstracts was developed for each database (S1 Table). All published articles (until 31st January 2024), independent of region, or publication year and written in English were searched using all search strategies. Results from manual searches were exported to EndNote. After exporting every database search result to EndNote, the data was merged and any duplicates were removed.

### Eligibility Criteria for Studies to be Included

The studies reported on the effectiveness of mebendazole and/or albendazole against soil-transmitted helminthes (STHs) in children are eligible for inclusion in this review. We included randomized and non-randomized clinical trials, such as randomized double/single-blind or double/single-blind placebo trials, single-blinded non-inferiority trials, open-label trials, prospective cohort studies, and cross-sectional studies. The study did not include any gray literature. Furthermore, all forms of reviews, conference abstracts, commentary, editorials, protocols, letters to the editor, personal opinions, non-human or in vitro studies, treatment with solely other anti-helminthic drugs, or studies that demonstrated the effectiveness of albendazole or mebendazole in patients other than preschool-age children and school-age children, as well as those with incomplete data were excluded from the study.

### The Study Selection Procedure

Titles and abstracts of every record found by the search strategy were independently checked by two authors (TK and TB). Then, eligibility was determined and full-text copies of publications thought to be possibly relevant were retrieved. Individual judgments that disagreed were settled by discussion in the presence of third author (KB). All studies excluded after full-text were assessed, and their reasons for the exclusion were indicated in the supplementary file (S2 Table). Key characteristics of the studies included in the review were extracted based on the format prepared by the *PICOS* model guide (Table 1).

**Table 1:** PICOS strategies

PICOS	Characteristic criteria for inclusion
P: population	The study participants are, children aged < 18 years and living in geo-helminthic infections endemic regions, targeted by the WHO’s deworming program, confirmed to have any geo-helminthic infections, fulfilled the inclusion criteria included in each study. STH-infected pre-school and school-age children enrolled in the individual studies conducted at health facilities, school compound, or community level, and treated with albendazole or mebendazole or with combination of these drugs with other (albendazole or mebendazole plus ivermectin or other drugs, Albendazole plus mebendazole or others) were the study participants.
I: intervention/exposure	Treatment with albendazole (400 mg/kg) and/or mebendazole (500 mg/kg) or other combination treatment
C: comparison/ control	Any placebo or anthelminthic drugs other than albendazole or mebendazole,
O: outcomes	ERR/CR achieved by albendazole or mebendazole or any form of their combination therapy against STH parasites among children < 18 years old. These include the CR of albendazole alone or with a combination of other drugs or the CR of mebendazole alone or with the combination of other drugs. In addition, ERR recorded for soil-transmitted helminthes infected children on day 14, 21, or other days/weeks, diagnosed by any valid procedures (Kato Katz, McMaster, concentration (sedimentation or floatation)], direct wet mount, and molecular methods (qPCR).
S: studies	The studies reported on the effectiveness or efficacy of mebendazole and/or albendazole against soil-transmitted helminthes (STHs) in children are eligible for the study. Randomized controlled trials (randomized double/single-blind or double/single-blind placebo trials, single-blinded non-inferiority trials) and non-randomized interventional studies (cross-sectional studies, prospective or longitudinal cohort studies) on the efficacy of mebendazole or albendazole or mebendazole plus albendazole or albendazole plus other drugs, mebendazole plus other drugs against soil-transmitted helminths (*Ascaris lumbricoides*, *Trichuris trichura*, and hookworms) in children (pre-school and school-age) were the study types included in the review. Studies from any regions targeted by WHO deworming programs (Africa, Asia, and South America), and published before 31st January 2024 were included in the study.

### Data Extraction

The study team created a format for data extraction. Two authors (TK and TB) separately extracted the data from each eligible article. The study involved gathering information on the following aspects: Key characteristics of the children (both asymptomatic and symptomatic), study settings (health facilities, schools, or communities), socio-demographic characteristics [age, sex (male or female), mean age, age range], geographical location (continents and country), sample size, diagnosis methods [Kato Katz, McMaster, concentration (sedimentation or floatation)], direct wet mount and molecular methods (qPCR), infection intensity (light, moderate, and heavy), egg per gram of stool (EPG) in geometric mean (before and after treatment), treatment options (albendazole or mebendazole alone or with combination of other anthelminthic drugs), follow-up weeks, and the treatment outcomes [ERR and CR] were collected from each eligible study. A third researcher verified the consistency of the target data after two researchers had independently searched for and retrieved it. A disagreement over individual judgments was resolved through discussion in the presence of the third reviewer.

### Eligibility Criteria for Studies to be Included

Studies that reported the effectiveness of mebendazole and/or albendazole against STHs in children were eligible for inclusion in this review. We included randomized and non-randomized clinical trials, such as randomized double/single-blind or double/single-blind placebo trials, single-blinded non-inferiority trials, open-label trials, prospective cohort studies, and cross-sectional studies. The study did not include any gray literature. Furthermore, all forms of reviews, conference abstracts, commentary, editorials, protocols, letters to the editor, personal opinions, non-human or in vitro studies, treatment with solely other anti-helminthic drugs, or studies that demonstrated the effectiveness of albendazole or mebendazole in patients other than preschool-age children and school-age children, as well as those with incomplete data were excluded from the study.

### The Study Selection Procedure

Titles and abstracts of every record found by the search strategy were separately checked by two authors (TK and TB). Then, eligibility was determined and full-text copies of publications thought to be possibly relevant were retrieved. Individual judgments that disagreed were settled by discussion in the presence of a third author (KB). All studies excluded after full-text were assessed, and their reasons for the exclusion were indicated in the supplementary file (S2 Table).

### Data Extraction

The study team created a format for data extraction. Two authors (TK and TB) separately extract the data from each eligible article. The study involved gathering information on the following aspects: Key characteristics of the children (both asymptomatic and symptomatic), study settings (health facilities, schools, or communities), socio-demographic characteristics [age, sex (male or female), mean age, age range], geographical location (continents and country), sample size, diagnosis methods [Kato katz, McMaster, concentration (sedimentation or floatation)], direct wet mount and molecular methods (qPCR), infection intensity (light, moderate, and heavy), EPG in geometric mean (before and after treatment), treatment options (albendazole or mebendazoles alone or with combination of other anthelminthic drugs), follow-up weeks, and the treatment outcomes [ERR and CR] were collected from each eligible study. A third researcher verified the consistency of the target data after two researchers had independently searched for and retrieved it. A disagreement over individual judgments was resolved through discussion in the presence of the third reviewer.

### Risk of Bias (Quality) Assessment

The risk of bias for each article included in the study was independently evaluated by the two research team members (TK and KB) following the Cochrane risk of bias-2 (RoB 2) tool [11] for randomized controlled trials (RCT), and risk of bias in non-randomized Studies - of Interventions-1 (ROBINS-1), for the non-randomized interventional studies [12]. Whenever the two authors encountered disagreement, there was a third author (TB) involved in solving the disagreement through discussion. The ROBINS-1 risk of bias assessment includes the bias due to confounding, bias in the selection of participants into the study, bias in the classification of interventions, bias due to deviation from intended intervention, bias due to missing data, bias in the measurement of the outcome, and bias in the selection of report results. Also, RoB-2 included the critical appraisal domains such as incomplete outcome data (attrition bias), reporting bias, blinding of personnel and participants (performance bias), blinding of outcome assessment (detection bias), random sequence generation (selection biases), and allocation concealment (selection biases). An overall risk of bias was subsequently classified as low, unclear, or high for each RCT study [11]. The bias risks were graded as low risk, moderate risk, serious risk, and critical risk for non-randomized interventional studies [12]. Consequently, a significant number of articles on RCT included in this review encountered a high risk of bias in the selection of the participants; allocation concealment (32.5%), and performance bias [blinding of outcome assessment (detection bias), 30%]. In addition, a substantial number of publications had low risks of performance bias (blinding of participants and person, *n* = 38/40, 95%), reporting bias (n = = 38/40, 95%), and random sequence generation bias (*n* = 33, 82.5%) [S3 Table (a)]. Likewise, all non-randomized interventional studies had a low risk of one of the appraisal domains, which was biased in the classification of interventions, 100%. The majority of the studies (*n* = 23, 88.46%), had a low risk of biases to deviation from the intended intervention and missing data. On the other hand, several studies (53.8%) had a moderate risk of bias to confounding, bias in the measurement of the outcome, and bias in the selection of participants into the study. A serious risk of bias in the measurement of the outcome was found in three studies [S3 Table (b)]. Detailed assessment tools for each critical appraisal tool for risk of bias are attached as a supplementary document (S4 Table).

### Strategy for Data Synthesis

The extracted data, along with comprehensive details on the first author’s name, study region, year of publication, study design, diagnosis method, treatment options, important study participant characteristics, infection intensity, treatment outcomes, and other relevant data, were entered into a Microsoft Excel spreadsheet. The Cochrane Review Manager (version 5.4) was used to analyze the data for both qualitative and quantitative synthesis. For every trial, the combined estimated ERR was provided. For each study, the standard error of the mean (SE) was calculated from the standard deviation obtained using the formula, *StDev* = Öp (1 − p), where p is a proportion of the population with the treatment success. Then, SE was calculated from the *StDev* using the formula, *SE* = *StDev/*Ön, where n is the sample size (sample size of each study). The Q (χ^2^) and *I*^2^ tests were used to evaluate the heterogeneity among the studies. Significant statistical heterogeneity was defined as a p-value of the χ^2^ test less than 0.05 for the Cochrane’s test. 25%, 50%, and > 75% of *I*^2^ were considered to indicate moderate, medium, and high heterogeneity, respectively. Due to significant variability (*I*^2^ > 75%, *p* < 0.05), the combined estimated ‘Efficacy of Mebendazole and Albendazole against soil-transmitted helminthic infections in children’ was determined using a random effects (DerSaimonian and Laird) model. All reported p values were two-sided and statistically significant if *p* < 0.05. Forest plots were used to display point estimates and confidence intervals. Publication bias for studies included in the meta-analysis was assessed quantitatively using Egger’s test and qualitatively by constructing a funnel plot and looking for asymmetry. Furthermore, for the sensitivity analysis, multivariate meta-regression model was conducted to investigate the role of the subgroups on the observed high heterogeneity (using R-software version 4.2.0, ‘*Meta*’, ‘*Metafor*’). Hence, nine subgroups were identified: region of the study, study year, study designs, treatment options, diagnosis types, EPGs at baseline, age of the children, follow-up weeks, and countries income as per the new World Bank classification. The model’s goodness of fit was evaluated using the residual analysis (R2), Bayesian Information Criterion (BIC) and Akaike Information Criterion (AIC). Also, the outlier ERR of the two drugs against the STHs were analyzed using Grubbs test on the R-software.

## Results

**Fig. 1:**
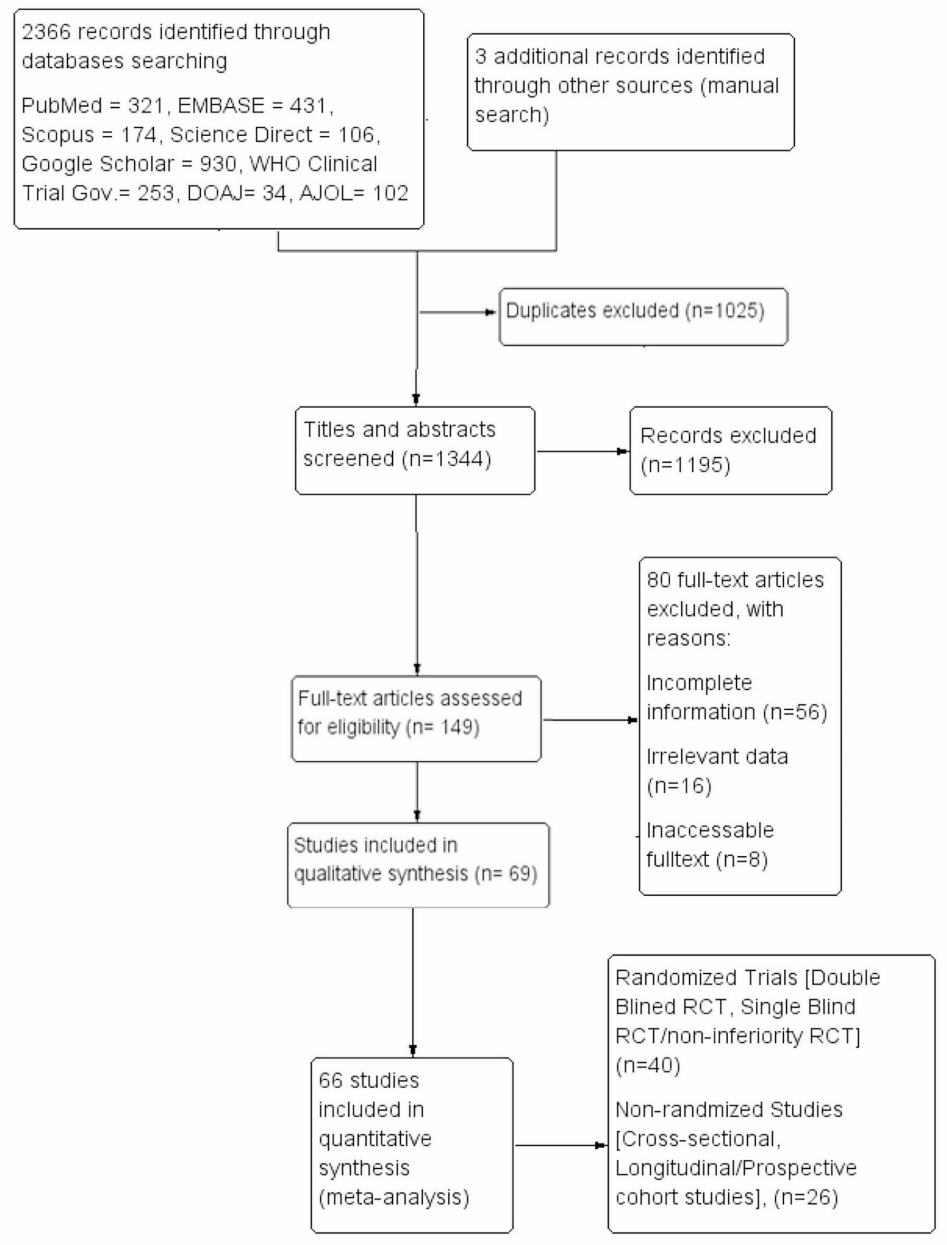
Study flow diagram

A total of 2369 records were identified through PubMed (*n* = 321), Scopus (*n* = 174), EMBASE (*n* = 431), Science Direct (*n* = 106), WHO Clinical Trial.gov. (*n* = 253), Directory Online Access Journal (*n* = 34), African Journal Online (AJOL) (*n* = 102) and Google Scholar (*n* = 930) databases. A total of 1025 records unrelated and ineligible records were excluded at the beginning of screening. Full texts of 149 eligible articles were reviewed, and 69 articles were included in the study from which 66 were used for the meta-analysis (Fig. 1).

### The Characteristics of the Included Studies

In this study, articles included for the review were 69, and those selected for meta-analysis were 66. These studies were conducted between 1990 and 2021 on different continents and published between 1991 and 31st January 2024. Most of the studies included were from Africa (37/69), followed by Asia (26/69), and South America (3/69); three studies included data from different continents (Africa, Asia, and South America). Most of these studies have included many treatment options, and the number of treatment options included in the meta-analysis was 140. These treatment options are, for *T. trichiura* (albendazole = 83, mebendazol = 36), *A. lumbricoides* (albendazole *=* 71, mebendazol = 38), and Hookworms (albendazole = 58, mebendazol = 35) (Table 1). The majority of the studies were conducted in school (63/69), and only four were undertaken at the community level (4/69), in health facilities (1/69) and one study didn’t indicate the study setting. A total of 67,083 pre- and school-age children, whose mean age was 10.8 years and ranged from 2 to 18 years, were included in the study, except one study where maximum age was 19. The prevalence of soil-transmitted helminthic (STH) infection was 35% (*n* = 23,504/67,083). The main diagnosis tool used in most of the studies was Karo Katz (*n* = 57, 82.6%). Only a few studies used McMaster alone (*n* = 5), McMaster plus concentration (*n* = 2), Kato Katz plus wet mount (*n* = 2), and others (*n* = 3). Only two of these studies used molecular methods (qPCR) as a confirmatory test for parasite identification and egg counting. The overall CR of albendazole against the *T.trichiura*, *A. lumbricoides*, and Hookworms were 50.8%, 91.3%, and 78.32% respectively. Likewise, mebendazole showed CRs of 48.15%, 92.8%, and 49.32% against *T. trichiura*, *A. lumbricoides*, and Hookworms respectively (Table 2).

**Table 2:** A summary of basic characteristics of the studies included in the review

Authors ID	Treatment type	Study design		T. trichiura		A. lumbricoides		Hookworm	
		Sample size	STH positive						
				ERR	CR	ERR	CR	ERR	CR
Adegnika et al., 2014	Alb (400 mg)	436	266	7	40	94	85	54	54
	Alb (400 mg)*			58	67	97	91	92	92
	Alb (400 mg)**			91	83	99	91	93	93
Albonico et al.,1994	Alb (400 mg)	2648	402	73.3	10.5	99.6	98.9	99.7	56.8
	Meb (500 mg)			81.6	14.2	99.3	97.8	82.4	22.4
Albonico et al., 2002	Meb (500 mg)	1329	448	83.6	25.2	96.1	98	67	13.2
Albonico et al., 2007	Alb (400 mg) GSK	2140	1506	71.7	28.6	92.6	97	87.1	74.3
	Alb (400 mg) Royal drug			71.4	26.6	93.8	95	80.8	53.3
	Alb (400 mg) Curex			63.2	28	91.9	82.6	73.1	50.7
Albonico et al., 2003	Meb (500 mg)	904	458	86.9	22.8	99.5	93.8	37	4.4
	Meb (500 mg) plus levamisole (40 or 80 mg)			87.9	17.1	99.6	100	92.9	26
Amelia et al., 2013	Meb (500 mg) plus Pyrantel Pamoate (10 mg)	288	130	91.8	89.5	97.4	98.5	ND	ND
	Meb (500 mg)			97	78.5	99.1	95.4	ND	ND
Antu and Nugraha, 2019	Alb (400 mg)	807	185	20.4	66.7	100	100	ND	ND
	Alb (400 mg) plus Levamisole (25 mg)			7.6	94.7	100	100	ND	ND
	Meb (500 mg) plus Levamisole (25 mg)			8.8	92.3	100	100	ND	ND
	Alb (400 mg) plus Levamisole (25 mg)			7.6	94.7	100	100	ND	ND
Barda et al., 2018	Alb (400 mg) plus moxidectin (8 mg)	942	634	99.8	ND	ND	ND	ND	ND
	Alb (400 mg) plus Oxantel Pamoate (25 mg/kg)			98.5	ND	ND	ND	ND	ND
Bartoloni et al., 1993	Alb (400 mg)	117	48	45.7	33.3	100	100	92.8	81.8
	Meb (500 mg)	62	40	17.2	60	100	100	62.4	17.2
Belizario et al., 2003	Alb (400 mg)plus Ivermectin (200 µg)	2284	784	35.1	97.5	97.5	99.5	ND	ND
	Alb (400 mg)			31.5	54	93	93	ND	ND
	Alb (400 mg)plus Diethylcarbamazine (6 mg)			20	79.4	96.6	96.6	ND	ND
Dalimunthe et al., 2007	Meb (500 mg)	326	311	98.1	97.3	99.9	96.9	100	100
	Meb (100 mg) plus Pyrantel Pamoate(10 mg)			97.9	94.2	100	100	100	100
Ekenjoku et al., 2013	Meb (500 mg)	400	284	100	100	100	100	ND	90.4
Eshetu et al., 2020	Meb (500 mg)	300	120	ND	ND	ND	ND	68.9	30.8
	Meb (multiple dose)			ND	ND	ND	ND	99.5	96.1
Ejigu et al., 2021	Meb (500 mg)	422	296	ND	ND	76.4	60	53.1	32.4
Flohr et al., 2007	Meb (500 mg)	271	168	ND	ND	ND	ND	52	38
Getachew, 2014	Alb (400 mg) Bendex	679	418	24.4	ND	98.7	ND	88.7	ND
	Alb (400 mg) Ovis®			20.4	ND	97.8	ND	98.1	ND
Gebreyesus et al., 2024	Alb (400 mg)	3162	2030	68.3	49.5	84.5	71.5	98.8	97.2
Humphries et al., 2017	Alb (400 mg)	140	82	ND	ND	ND	ND	61	36
Husin et al., 2022	Alb (400 mg)	449	199		61.2		87.5	100	100
	Meb(500 mg)				65.6		31		83.3
Iqbal et al., 2021	Alb (400-450 mg)	296	192	ND	ND	ND	ND	75	75
	Meb (300 -350 mg)			ND	ND	ND	ND	71	71
Ismail et al., 1999	Alb (400 mg)	176	155	70.3	43.6	ND	ND	ND	ND
	Alb (400 mg) plus Diethylcarbamazine (6 mg)			69	29.8	ND	ND	ND	ND
	Alb (400 mg) plus ivermectine (200 µg)			93.8	79.3	ND	ND	ND	ND
Kabatende et al., 2023	Alb (400 mg)	4998	1526	17.6	40.3	94.6	95.1	97.4	96.7
Keller et al., 2016	Alb (400 mg) plus Moxidectin (8 mg)	379	294	97.4	62.5	100	75	99.7	81.8
	Alb (400 mg) plus Moxidectin (16 mg)			98.4	61.9	100	100	98.3	80
	Alb (400 mg)plus Moxidectin (24 mg)			98.6	69.2	89.3	20	99.6	90
Knopp et al., 2010	Alb (400 mg) plus placebo	1240	610	40.3	9.8	100	100	94	59
	Alb (400 mg) plus ivermectin			91.1	37.9	99.9	92.9	78.7	66.7
	Meb (500 mg)plus placebo			66.7	18.6	99.79	77.8	99.8	35.3
	Meb (500 mg) plus ivermectin (200 µg)			96.7	55.1	100	100	78.7	25.7
Kihara et al., 2007	Alb (400 mg) plus praziquanto	2300	441	45	18.2	100	100	96	96
Krücken et al., 2017	Alb (400 mg)	1182	698	ND	ND	75.4	86.7	ND	ND
Levecke et al., 2014	Meb (500 mg)	5830	2589	0	ND	97.5	ND	84.4	ND
				56.2	ND	99.8	ND	71.9	ND
				ND	ND	ND	ND	79.1	ND
				65.9	ND	98.6	ND	65.4	ND
				51.2	ND	97.1	ND	74.6	ND
				76.8	ND	93.1	ND	95	ND
Legesse et al..,2004	Alb(400 mg)	717	703	69.8	17.1	99.9	92.5	ND	ND
	Meb (100 mg) East Afr			88.5	27.9	99.9	93	ND	ND
	Meb (100 mg) Indian			96.5	53.5	99.9	99	ND	ND
	Meb (100 mg) SouthAfrica			99.1	89.8	99.9	96.5	ND	ND
Lubis et al., 2012	Alb (400 mg)	ND	229	ND	ND	99.3	96.7	ND	ND
	Meb (500 mg)			ND	ND	100	100	ND	ND
Mani et al., 2002	Alb plus Diethylcarbamazine	646	321	84	81.58	96.6	74.3	94.2	89.5
Matamoros et al.., 2021	Alb (400 mg)	377	176	47.7	4.2	ND	ND	ND	ND
	Alb 1 × (400 mg) plus ivermectin (600 µg)			96.7	88.6	ND	ND	ND	ND
	Alb (400 mg)**			72.1	33.3	ND	ND	ND	ND
	Alb (400 mg) plus ivermectin (600 µg) for 3 days			100	100	ND	ND	ND	ND
Moser et al., 2018	Alb (400 mg) plus pyrantel pamoate(20 mg), and oxantel pamoate(20 mg/kg)	1524	533	99.6	93	99.9	90.9	99.9	84.1
	Alb (400 mg) plus oxantel pamoate (20 mg)			100	100	99.9	95.8	99	52.9
	Meb (500 mg) Pyrantel pamoate (20 mg), and oxantel pamoate (20 mg)			98.8	88.5	100	100	99.6	69.6
Müller et al., 2016	Alb (400 mg)	149	90	46	1.1	94.3	97.2	ND	ND
Muchiri et al., 2001	Meb (600 mg)***	1186	726	93.4	60.6	99.4	79.6	66.3	50
	Meb (600 mg)****			94.1	68.3	99.9	97.5	85.1	55
	Alb (600 mg)			90.5	67.8	99.6	83.5	96.7	92.4
Nadyne et al., 2017	Meb (500 mg) (2x) 3days	410	259	39.9	60.7	94.5	93.9	52.9	70.8
Nasution et al., 2014	Alb (400 mg)	212	116	54.5	66	ND	ND	ND	ND
	Alb (400 mg) plus Diethylcarbamazine (6 mg)			60.7	60	ND	ND	ND	ND
Nkengazong et al., 2010	Alb (600mg)^*^	420	178	55.3	84.6	52.2	82	100	100
Ngonjo et al., 2015	Alb (400 mg)	1396	731	100	100	94.8	98.4	ND	ND
Nisha et al., 2021	Alb (400 mg)	68	58	55.7	41.4	99.9	93.1	ND	ND
Nontasut et al., 1997	Alb (400mg) ^**^	ND	ND	ND	33.3	ND	83.3	ND	91.6
	Meb (100 mg)			ND	93.3	ND	100	ND	81.8
	Mab (25 mg)			ND	38.1	ND	93.3	ND	64
	Meb (50 mg)			ND	41.4	ND	90.5	ND	48.6
	Meb (75 mg)			ND	51.4	ND	88.2	ND	35.3
Norhayati et al., 1997	Alb (400 mg)	205	123	49.1	5.5	99.9	97.4	96.6	93.1
Ortiz et al., 2002	Alb (400 mg)	ND	ND	98.4	58	99.9	91	ND	ND
Patel et al., 2020	Alb (200 mg) Preschool age children	1343	291	63.8	9.5	99.9	83.3	ND	ND
	Alb (400 mg) Preschool age children			87.1	17.4	99.6	66.7	100	100
	Alb (600 mg) Preschool age children			88.5	27.8	100	100	0	ND
	Alb (400 mg) School age children			82	16.3	99.9	88.9	0	0
	Alb (600 mg) School age children			88.4	25.6	100	100	100	100
	Alb (800 mg) School age children			78.8	17.1	99.9	84.6	100	100
Palmeirim et al., 2018	Meb (500 mg)	354	206	71.7	6.8	100	100	52.7	13
	Meb (100 mg) (multiple dose			98.1	42.9	100	98	99.8	97.9
Palmeirim et al., 2020	Meb (chewable)	1465	500	73.3	9.8	98.7	95.3	38.1	12.7
	Meb (solid)			74.2	7.3	99.8	97.8	28.1	11.2
Payne et al., 2016	Meb (500 mg)	948	50	14	14.29	100	100	77	70.4
Putra et al., 2005	Alb (400 mg)	434	333	ND	ND	100	100	ND	ND
Rahman et al., 1996	Alb (400 mg)	294	192	ND	83.4	ND	87.3	ND	89.1
	Meb (400 mg)			ND	33.3	ND	83.3	ND	
Rochmah et al., 2016	Alb (400 mg)	65	28	62.4	12.8	ND	ND	ND	ND
Sam, 2011	Alb (400 mg)	2549	161	76	84.6	100	100	96	83.6
Samuel et al., 2014	Alb (400 mg)	298	263	83.1	30.8	99.9	96.6	99.8	97.4
Sam-Wobo et al., 2021	Alb (400 mg)	282	151	69.2	ND	99.7	ND	99.4	
Sapulete et al., 2020	Alb (400mg) ^**^	600	392	55.3	60	ND	ND	ND	ND
	Alb (400 mg) plus Pyrantel Pamoate (10 mg)***			44.7	40	ND	ND	ND	ND
Silber et al.,2017	Meb (500 mg)	726	295	59.7	42	97.9	83.7	100	100
Soukhathammavong et al., 2012	Alb (400 mg)	465	200	67	33.3	100	92.9	86.7	36
	Meb (500 mg)			66	27.9	100	93.3	76.3	17.6
Speich et al., 2012	Alb (400 mg)	701	353	45.6	14.5	100		ND	81.8
	Alb (400 mg) (plus Nitazoxanide (1000 mg)			54.9	16	100		ND	85.5
Subba and Singh, 2020	Alb (400 mg)	300	10	100	100	81.4	55.5	ND	ND
Speich et al., 2015	Alb ( 400 mg) plus ivermectin (200 µg)	650	440	94.5	30	100	98	17.5	22.2
	Alb (400 mg) plus Meb (500 mg) (1x)			51.6	9	99.9	97.5	95.4	250
	Alb (400 mg) plus Oxantel Pamoate (20 mg)			99.2	74	99.9	97.9	92.7	47.8
	Meb (500 mg)			58.5	9	100	95.5	90.9	45.5
Speich et al., 2016	Alb (400 mg) plus Ivermectin (200 µg)	650	405	94.9	28	99.9	97.8	94.6	44.7
	Alb (400 mg) plus Meb (500 mg)			54.9	8.9	100	100	94.1	48.8
	Alb (400 mg) plus Oxantel Pamoate (20 mg)			99.2	68	99.9	97.7	91.9	48
	Meb (500 mg)			55.6	7.7	99.9	95.4	60.3	24.4
Sungkar et al., 2019	Alb (400 mg)**	246	192	91	61	100	97	100	100
Suteno et al., 2020	Alb (400 mg)	463	235	75.5	52.5	ND	ND	ND	ND
	Alb (400 mg) plus Meb (500 mg)			93.5	71.1	ND	ND	ND	ND
Tefera et al., 2015	Alb (400 mg)	715	326	99.9	99.4	99.9	59.9	99.9	93.7
Vercruysse et al., 2011	Alb (400 mg)	8841	2319	99.9	85.7	99.1	93.3	99.9	98.9
				100	100	98	98	99.6	52
				99.6	100	99.2	100	99.5	87.4
				99.9	47.4	93	99.3	91.9	87.1
				98.9	88.9	98.9	95.2	81.6	74.4
				100	21	82.6	96.4	92.6	86.6
Walker et al., 2021	Alb (400 mg)	645	441	65.1	ND	98.5	ND	85.6	ND
				61	ND	96.5	ND	82	ND
				29	ND	95.3	ND	63.3	ND
Welsche et al., 2023	Alb (400 mg) plus Moxidectin (8 mg)	771	550	96.8	34.3	100	100	98.8	75
	Alb (400 mg) plus Ivermectin (200 µg/kg)			99	54	100	96.4	97.4	62.9
	Albendazole (400 mg)			86.2	26.3	100	91.7	100	100
Worku, 2018	Alb (400 mg)	393	138	38	42	99.9	98.2	54.4	71.4
Yahia et al.., 2019	Alb (400 mg)	314	78	ND	ND	96.1	93.8	91.2	88.2
Yap et al., 2013	Alb (400 mg)**	250	211	88.8	19.6	99.9	91.5	99.1	96.7
Zeleke et al., 2020	Meb (500 mg)	504	130	56.3	28.6	99.9	96.9	49.6	23.1

*NB*: Alb = Albendazole, Meb = Mebendazole, Iver = Ivermectine, RCT = randomized controlled trials, DBRCT = Double blind randomized controlled trials, CT = clinical trial, CR = Cure rate, ERR = egg reduction rate, studies with asterisk: * = double (2x) dose, ** = triple (3x) dose, *** = single dose for three days, **** = multiple dose

The number of children infected with *T. trichiura*, *A. lumbricoides*, and Hookworms was 16,929, 14,102, and 11,526, respectively. Concerning infection intensity (data from 38 studies), the number of children infected with *T. trichiura* and who had light, moderate, and heavy infections were 6881, 1916, and 341 respectively. For *A. lumbricoides* infection, documented light, moderate, and heavy infections were 5851, 2288, and 2575, respectively. Likewise, for hookworm, the numbers of children with light, moderate, and heavy infections were 5815, 290, and 131, respectively. The EPG at enrollment and post-treatment (only report of 45 studies) for *T. trichiura* were 825.9 and 240.4, for *A. lumbricoides*, 6830 and 147.6, and for hookworms, 551 and 90.8, respectively. Follow-up weeks post-treatment of anthelmintic drugs were 1–2 for 21 studies, 2–3 weeks for 27 studies, 4 weeks for 15 studies, and five studies each had 6, 7, 8, 10, and 12-week follow-up durations. One study was without information about the follow-up days [13]. Majority of the studies included in the meta-analysis were randomized clinical trial of different types (*n* = 40, 60.6%), followed by non-randomized interventional studies designated ascross-sectional, prospective cohort study, or longitudinal cohort studies) (*n* = 26, 39.4%) (S4 Table).

### Efficacy of Albendazole and Mebendazole Against *T. Trichiura*

**Fig. 2:**
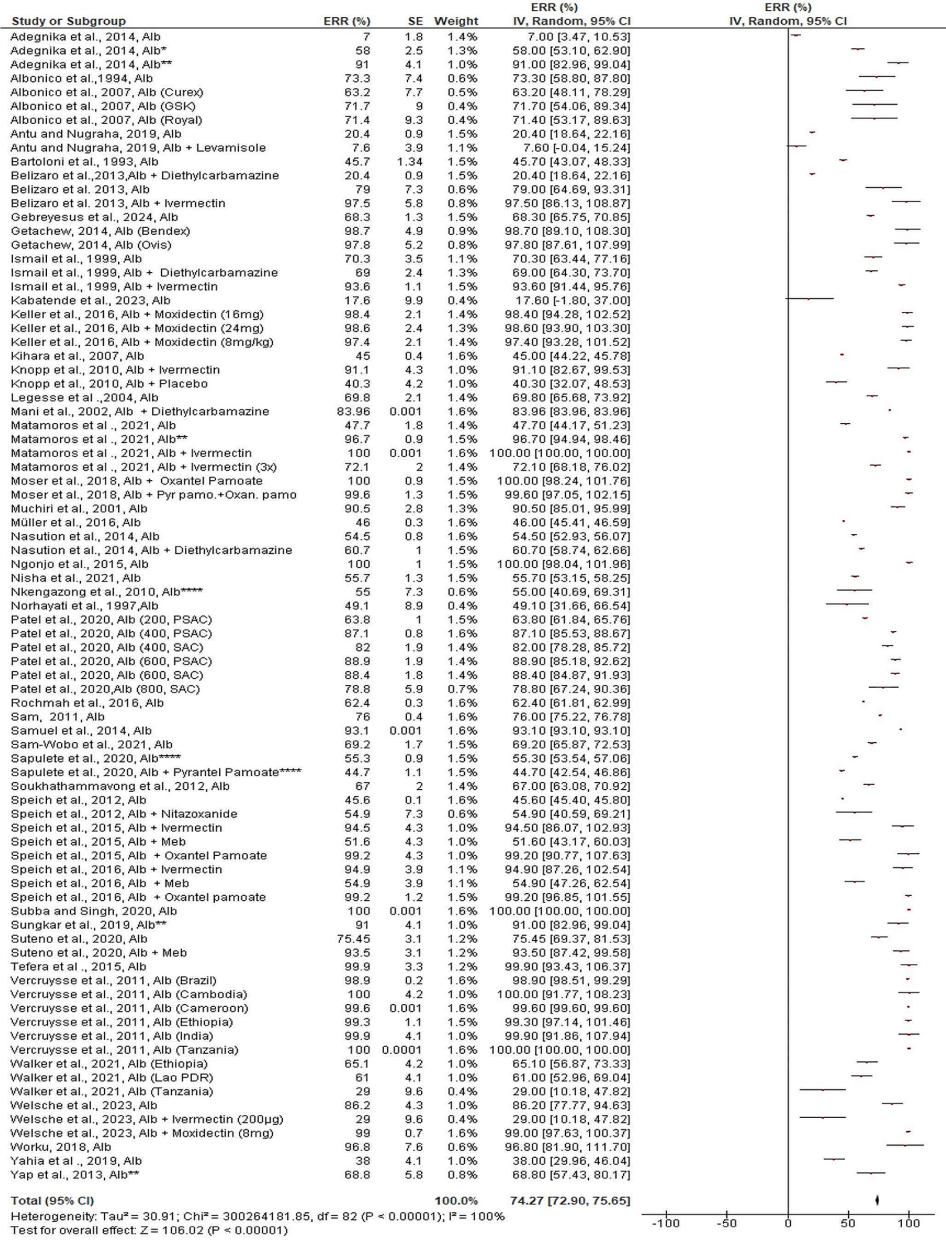
Pooled in vivo efficacy of albendazole against T. trichiura in pre-school and school age children. NB: Studies with asterisk (* = double dose, ** = triple dose, *** = single dose for three days, **** = multiple dose), Alb = Albendazole, Meb = Mebendazole, Pyr.Pamo + Oxan.pamo = Pyrantel pamoate and Oxantel Pamoate

**Fig. 3:**
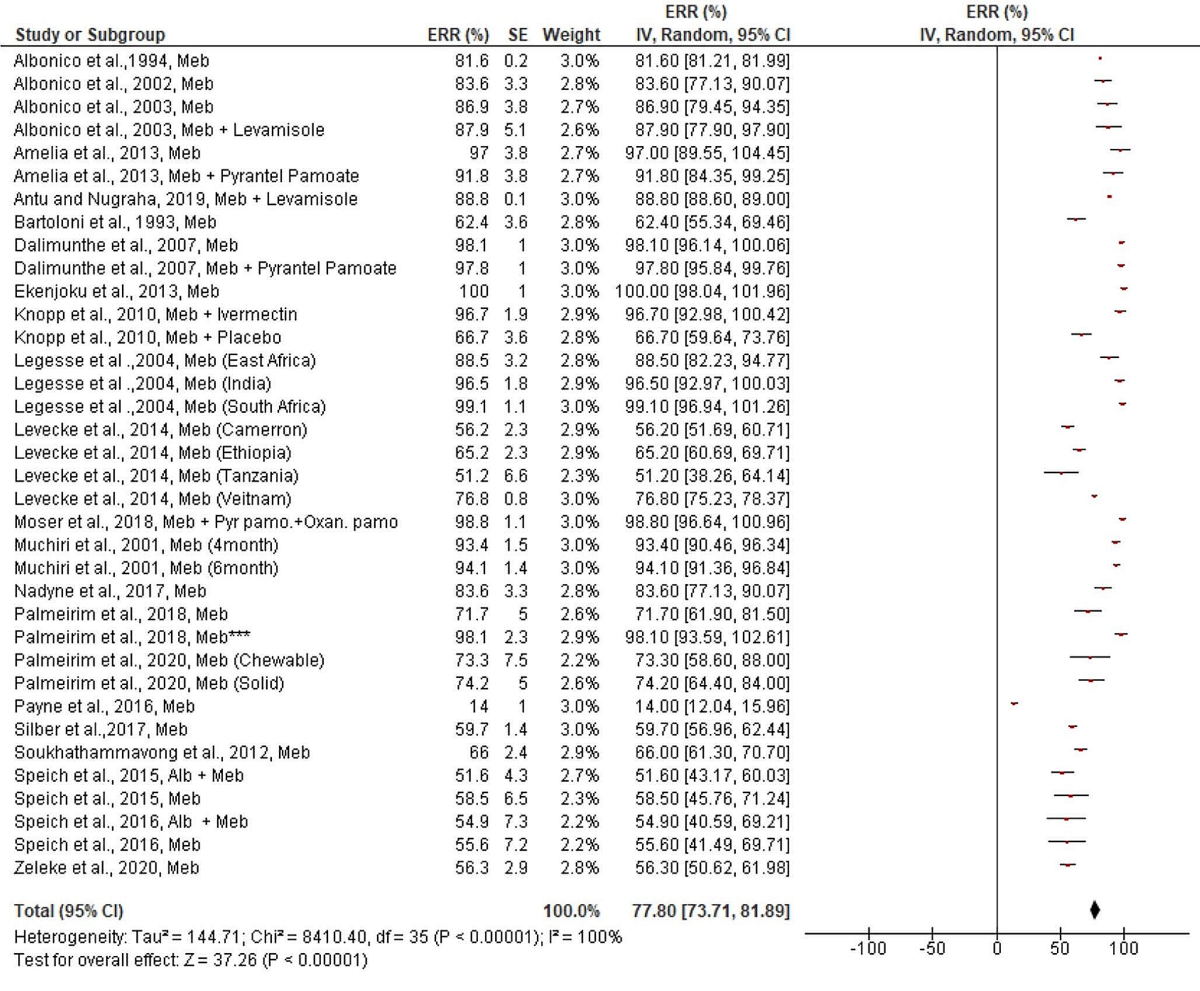
Pooled in vivo efficacy of Mebendazole against T. trichiura in pre-school and school age children. NB: Studies with asterisk (* = double dose, ** = triple dose, *** = single dose for three days, **** = multiple dose), Alb = Albendazole, Meb = Mebendazole, Pyr. Pamo + Oxan.pamo = Pyrantel pamoate and Oxantel Pamoate

The estimated pooled overall efficaciousness of albendazole against *T. trichiura* was 74.27% (95% CI: 72.95–75.69%) (Fig. 2). Studies differ from one another, as does the pharmacological regimen (single or in combination with other drugs). Figure 2 shows that the highest ERR (100%) was found in Africa: Kenya in 2014 [14], and Tanzania in 2009 [15], Asia: Cambodia in 2016 [15], and South America Honduras in 2019 [16]. The lowest ERR (7%) was reported from Africa (Gabon, single dose) [17]. The estimated pooled efficacy of mebendazole against *T. trichiura* was 77.8% (95%CI: 73.71–81.89%). The range of ERR observed is 14–100% (Fig. 3). Every study reported > 50% ERR, except for one study from Africa/Cameroon [18]. Mebendazole was found to be 100% effective in a single study reported from Nigeria, Africa, without combination treatment [19] (Fig. 3). The symmetrical funnel plot qualitatively demonstrated the absence of publication bias among the studies assessing albendazole and mebendazole’s effectiveness against *T. trichiura*. Also, egger’s regression test quantitatively showed insignificant bias as well (bias coefficient = 0.32, *p* = 99) (S1 Figure).

Regardless of the follow-up weeks, the efficacy of albendazole alone was slightly less than the total pooled estimated efficacy (72.72%, 95%CI: 71.73–73.74%). When combined with other anti-helminthic drugs, such as moxidectin at varied doses (8–24 mg/kg), and oxantel pamoate (20 mg/kg), its ERR showed increased and varied from 97.4 to 100%. Overall, there were also no statistically significant differences between albendazole taken alone or when combined with other drugs (χ^2^ = 3.05, *p* = 0.05, *I*^2^ = 67.3%). Also, mebendazole alone (74.06%, 95%CI: 65.17–82.95%) didn’t show significant differences (χ^2^ = 3.24, *p* = 0.07, *I*^*2*^ = 69.1%) than when it combined with other drugs (76.32%, 95%CI: 71.7–80.94%) although slight increment was observed (S2 Figure).

### Efficacy of Albendazole and Mebendazole against *A. Lumbricoides*

**Fig. 4:**
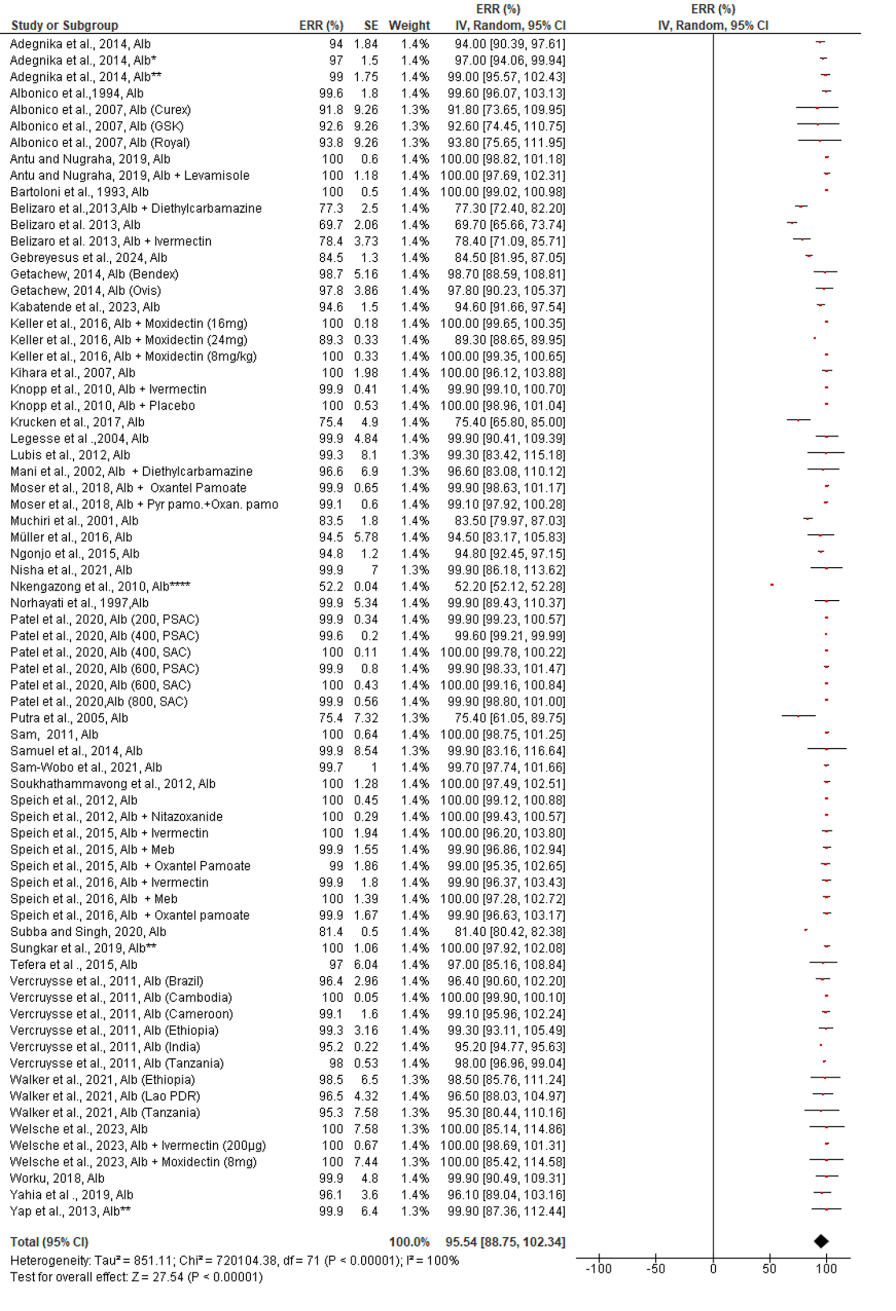
Pooled in vivo efficacy of Albendazole against A. lumbricoides in pre-school and school age children. NB: Studies with asterisk (* = double (2x) dose, ** = triple (3x) dose, **** = multiple dose), Alb = Albendazole, Meb = Mebendazole, Pyr.Pamo + Oxan.pamo = Pyrantel pamoate and Oxantel Pamoate

**Fig. 5:**
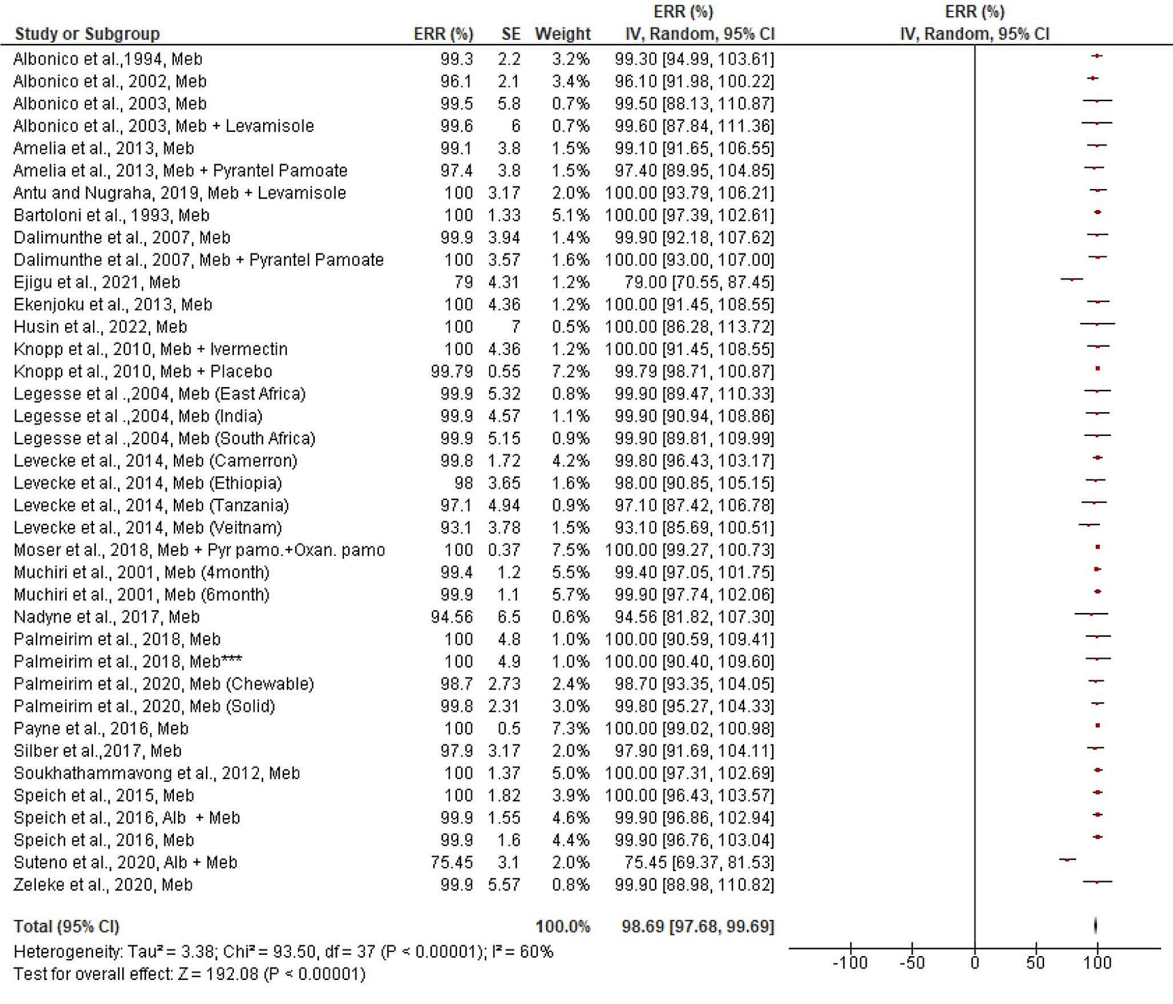
Pooled in vivo efficacy of Mebendazole against Ascaris lumbricoides in pre-school and school age children in different regions. NB: Studies with asterisk (*** = single dose for three days), Alb = Albendazole, Meb = Mebendazole, Pyr.Pamo + Oxan.pamo = Pyrantel pamoate and Oxantel Pamoate

In contrast, the estimated pooled efficacy of Albendazole against *A. lumbricoides* (95.54%, 95% CI: 88.75–102.34%) was much higher than that of *T. trichiura*, regardless of the drug’s mode of therapy (single or in combination with other drugs). Except for two reports, every study included in the review stated that albendazole had a greater ERR (> 80%) against *A. lumbricoides* (Fig. 4). Mebendazole, on the other hand, demonstrated outstanding efficacy against *A. lumbricoides* (98.69%, 95% CI: 97.68–99.69%). Except for one trial (Fig. 5), every study included in the review had an efficacy of more than 90%. Studies examining the effectiveness of albendazole and/or mebendazole against *A. lumbricoides* were included in the review showed a significant publication bias both quantitatively (Egger’s regression test) and qualitatively (asymmetric funnel plot) (bias coefficient = -24.5 (95%CI: 16.1–32.9, *p* < 0.0001) (S3 Figure).

The pooled estimated efficacy of albendazole and mebendazole when they were combined with other drugs or administered alone for the in vivo treatment of *A. lumbricoides*, significant differences were not observed (χ^2^ = 0.34, *p* = 0.56, *I*^*2*^ = 0%) and (χ^2^ = 2, *p* = 0.16, *I*^*2*^ = 49.9%) respectively (S4 Figure).

### Efficacy of Albendazole and Mebendazole against Hookworms

**Fig. 6:**
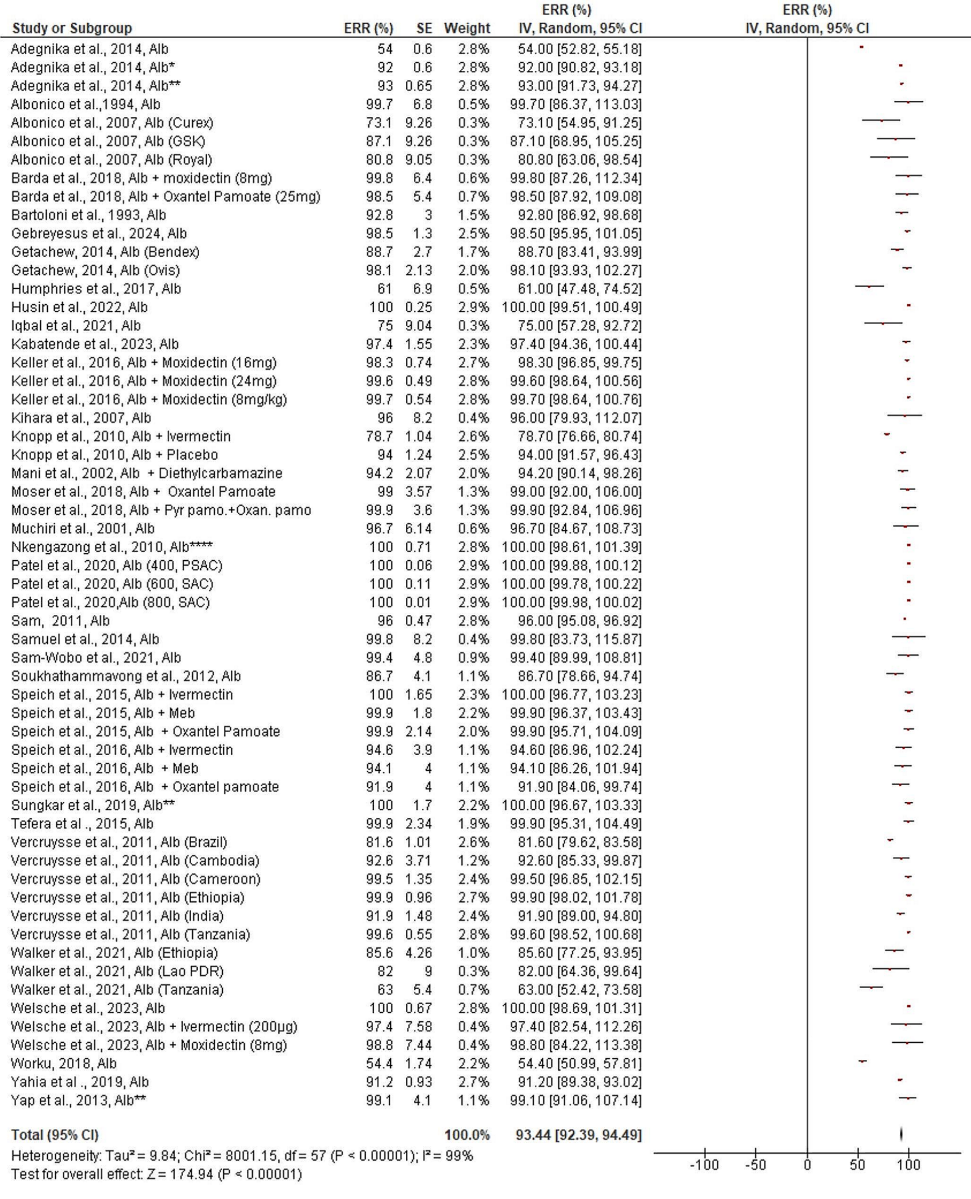
Pooled in vivo efficacy of Albendazole against Hookworms in pre-school and school age children. NB: Studies with asterisk (*** = single dose for three days), Alb = Albendazole, Meb = Mebendazole, Pyr.Pamo + Oxan.pamo = Pyrantel pamoate and Oxantel Pamoate

With a high level of heterogeneity (99%) among the efficacy trials included in the study, the pooled estimated ERR of albendazole against hookworm was 93.44% (95%CI: 92.39–94.49%), with its ERR varying from 54 to 100% (Fig. 6). When albendazole was taken together with other drugs (96.34, 95% CI: 93.14–99.54%), such as moxidectin, ivermectin, diethycarbamazine, and oxante pamoate, the ERR is significantly greater (χ^2^ = 5.07, *p* = 0.02, I2 = 80.3%) than when it was taken singly (92.41, 95% CI: 91.19–93.63%). Moxidectin and oxante pamoate are two of the drugs which combine with albendazole to treat hookworms with good efficacy (> 98% ERR). Additionally, albendazole combined with ivermectin demonstrated > 95% ERR (S5 Figure), except for one study (Knopp et al., 66.67%) (S5 Figure).

**Fig. 7:**
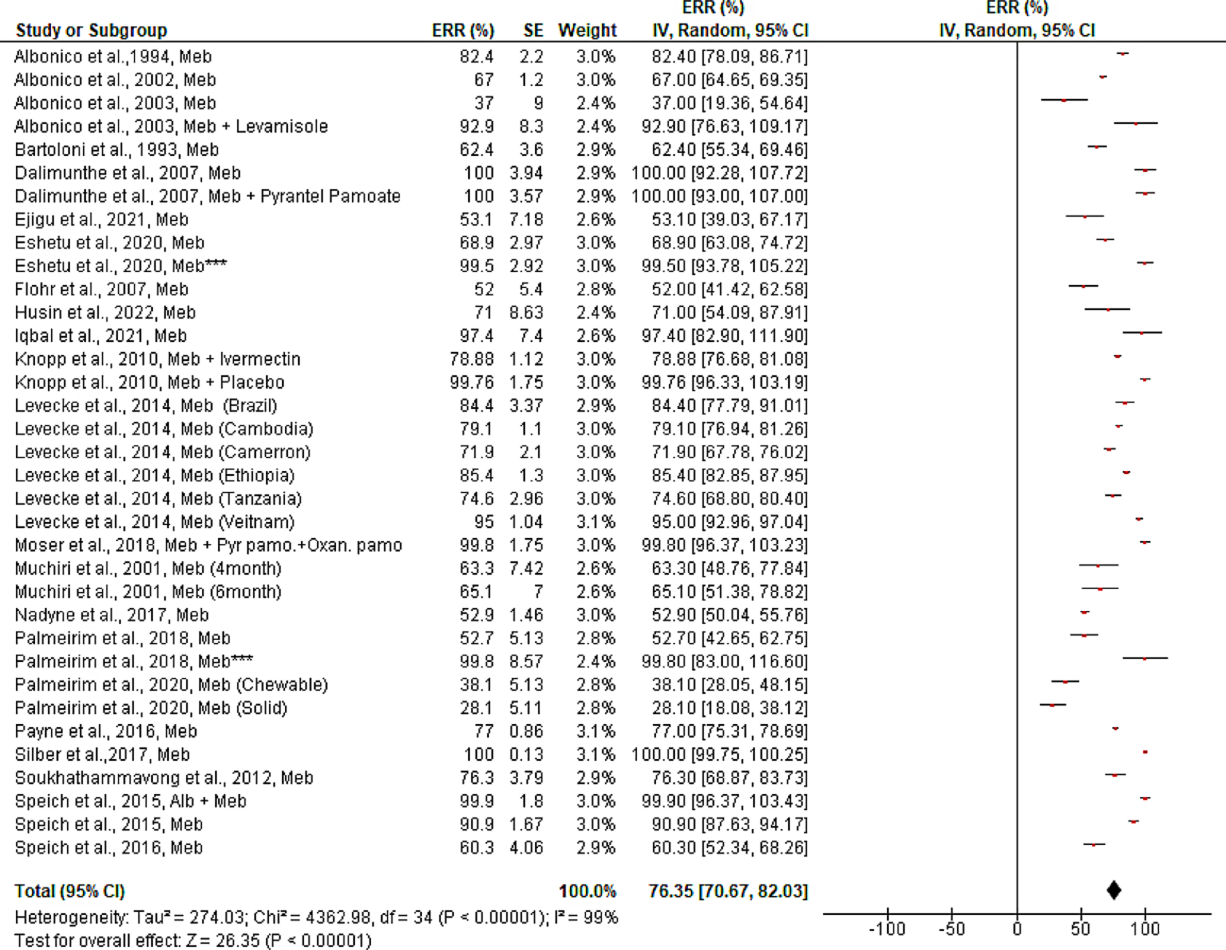
Pooled in vivo efficacy of mebendazol against Hookworms infection in pre-school and school age children. NB: Studies with asterisk (*** = single dose for three days), Alb = Albendazole, Meb = Mebendazole, Pyr.Pamo + Oxan.pamo = Pyrantel pamoate and Oxantel Pamoate

In contrast, mebendazole’s estimated efficacy against hookworms is substantially lower than albendazole (76.35, 95%CI: 70.67–82.03%) against the same parasite. The effectiveness differs between studies and geographical areas. Remarkably, 100% ERR against hookworm was demonstrated in two studies (one conducted in Ethiopia/Africa and the other in Indonesia/Asia) with a single dosage of mebendazole (500 mg/kg) or in combination with pyrantel pamoate (S5 Figure). Similarly, school children treated with single doses of mebendazole for three days in a row had > 99% effectiveness (Fig. 7). The studies included for the evaluation of the efficacy of albendazole and mebendazole against hookworms revealed the existence of publication bias both quantitatively [Egger’s regression test (bias coefficient = -6.39, 95%CI: -2.5 to -10.3)] and qualitatively (asymmetrical funnel plot) (S6 Figure).

### Sensitivity Analysis

A multivariate meta-regression model consisting of sub-groups was conducted to assess their effect on the observed high heterogeneity. Subsequently, the finding revealed that subgroups: study years after 2000, McMaster diagnosis type, and long follow-up weeks were significantly associated with reduced efficacy (ERR) of the albendazole and mebendazole drugs against *T. trichura*. While the combination of albendazole or mebendazole with other drugs, and the RCT studies had shown significantly improved efficacy against *T. trichura.* Also, the count of EPG was identified as one of the variables that negatively and significantly influenced the efficacy of albendazole or mebendazole against *A. lumbricoides*. In addition, the use of RCT and comprehensive mixed diagnostic tools [Kato-katz, PCR, concentration (floatation or sedimentation), and others] enhanced the efficacy of the drugs against *A. lumbricoides* (Table 3). However, none of the subgroups assessed had shown an effect on the pooled anthelminthic drug’s efficacy in the hookworms’ infections, rather, particular outlier data (17.5%) was found to significantly influence (Z = 3.69, *p* < 0.05) the pooled ERR against this parasite. Similarly, the lowest ERR (52.3%) reported in one of the studies included in the meta-analysis was identified as an outlier that could significantly influence the pooled anthelminthic drug’s efficacy against *A. lumbricoides*. Although the ERR reported (7%) for *T. trichura* was very far from the rest of the values for the efficacy of albendazole, significant differences (Z = 3.43, *p* > 0.05) were not observed. In addition, in all cases, the removal of the outlier values from the meta-analysis didn’t change or reduce the level of heterogeneity.

**Table 3:** A sensitivity analysis of subgroups effect on the efficacy of albendazole and mebendazole against STHs among pre-school and school age children

Variables	Covariate	T. trichiura				A. lumbricoides				Hookworms			
		Coefficient	SE	Z-value	*P*. vlue	Coefficient	SE	Z-value	*P*. vlue	Coefficient	SE	Z-value	*P*. value
	Intercept	73.53	22.9	3.21	0.00252 **	94.20	5.01	18.80	< 2e-16 ***	-10.23	81.39	-1.257	0.22
Region	Asia	-1.86	7.73	-0.24	0.81	-1.88	1.64	-1.15	0.26	-22.40	19.12	-0.117	0.91
	South America	11.31	9.73	1.16	0.25	-5.16	3.74	-1.5	0.18	88.46	77.44	1.142	0.27
	Africa *(Ref)*												
Sample/study year	Before 2000 *(Ref)*												
	2000–2010	-22.4	10.8	-2.06	0.04518 *	-0.11	2.40	-0.01	0.99	26.17	29.5	0.888	0.38
	2010–2015	-29.26	10.4	-2.8	0.00762 **	-3.64	2.472	-1.5	0.15	38.6	28.7	1.345	0.19
	After 2015	-11.48	9.85	-1.16	0.25	-0.98	1.934	-0.5	0.62	7.0	19.4	0.362	0.72
Country Income *	Low *(Ref)*												
	Low-middle	-13.18	9.33	-1.41	0.16	2.55	2.1	1.22	0.23	52.62	29.24	1.80	0.09
	Upper-middle	3.48	10.3	0.33	0.74	4.72	3.42	1.38	0.18	-14.03	53.54	-0.262	0.8
EPG	EPG at enrollment	-0.01	0.01	-1.26	0.21	-0.0018	4.738e-05	3.72	0.000768 ***	75.57	18.27	0.414	0.68
Treatment option	Alb *(Ref)*												
	Meb	10.07	8.2	1.2	0.22758	-0.3	1.41	-0.21	0.83	-74.62	13.71	-0.054	0.96
	Alb mix	20.95	6.4	3.27	0.00213 **	3.49	1.81	1.93	0.06	-50.38	17.44	-0.289	0.77
	Meb mix	22.23	8.9	2.5	0.01645 *	2.67	2.58	1.03	0.31	14.56	21.66	0.672	0.51
Age (year)	Mean	1.18	1.3	0.91	0.37	-0.58	0.25	-2.33	0.03 *	7.43	3.92	1.9	0.07
Study design	NRCT *(Ref)*												
	RCT	34.28	14.7	2.33	0.02495 *	11.1	2.699	4.13	0.0003**	-42.03	27.01	-1.56	0.13
Diagnosis method	Kato-Katz *(Ref)*												
	McMaster	-47.09	16.9	-2.9	0.00796 **	0.53	2.47	0.21	0.83	71.91	39.97	1.799	0.08
	Other **	-33.62	24.6	-1.37	0.18	7.42	2.62	2.83	0.007 **	-11.97	8.07	-1.48	0.14
Follow-up	Weeks	-6.36	2.71	-2.35	0.02368 *	-0.78	0.99	-0.78	0.44	22.07	16.94	1.3	0.21

*Classification was based on the New World Bank country classifications by income level: 2022–2023 (New World Bank country classifications by income level: 2022–2023). ** mix of different diagnosis methods [Kato-katz, PCR or Concentration (floatation or sedimentation), and others], EPG = egg per gram of stool, RCT = randomized controlled trials, NRCT = non randomized controlled trials, Alb = albendazole, Meb = mebendazole, Alb mix = Albendazole combined with other drug (s), Meb mix = Mebendazole combined with other drug (s)

## Discussion

The study estimated an overall prevalence of STHs of 35%, which was similar to the prevalence reported among school-age children in South-East Asia and the Western Pacific Regions (32.3%) and Low- and Middle-Income Countries (LMICs) (37.16%) [20, 21]. The pooled prevalence documented in the above two studies clarified that STHs continue to be the leading cause of public health problems, particularly for children. The biggest success in reducing this burden, particularly in endemic areas, was the scaling-up of the deworming program. In support of this, one study showed that the prevalence of STHs in children aged 5 to 14 years decreased from 44% in 2000 to 13% in 2018, primarily in sub-Saharan Africa. This decline was due to sustained delivery of preventive chemotherapy, improved sanitation, and economic development [22]. Also, a finding from the population-based interventions showed that a periodic application of these drugs resulted in marked decreases in the burden of STHs and reduced the magnitude of anemia in children after 4 years of its intervention [23]. To ensure achievement of the set elimination target of the World Health Organization (WHO) by 2030, designing comprehensive prevention and control approaches is essential. One of the areas that require adequate emphasis is the identification of risk factors, and designing of case-specific interventional strategies. Some of the risk factors identified are; limited knowledge and awareness of the food handlers on the transmission route of the STHs, poverty, lack of clean and potable drinking water supply, poor environmental hygiene, unsafe human or animal waste disposal systems, lack of a habit of vegetables or fruit disinfection, and, occupational type frequent exposure to the contaminates such as farming [24, 25].

Hence, this study is trying to synthesize evidence on the efficacy of the two widely used anthelmintic drugs (albendazole and mebendazole) in the preventive chemotherapy [26]. Albendazole and mebendazole have been extensively used worldwide for more than 30 years, both as stand-alone treatments or in combination with other drugs [27]. Although albendazole has been licensed for human use since 1982, still it is a drug of choice for treatment of STHs [26], the pooled efficacy estimated in the meta-analysis showed that, the ERRs of albendazole (400 mg in a single dose) against *T. trichiura, A. lumbricoides* and hookworms was found > 50%, > 95%, and > 90% respectively. The ERRs achieved by the albendazole against these STHs showed a satisfactory efficacy recommended by WHO [26]. However, in the same analysis, the efficacy reported (CR and ERR) to Albendazole and Mebendazole against *T. trichiura* was much lower than in other STHs, which could further strengthen the lower efficacy previously reported from all age groups of the population [28, 29].

While the two drugs showed different efficacy against hookworms, albendazole conveyed an excellent efficacy (93.47%) against hookworm than mebendazole (76.78%), which was consistent with the findings of previous studies [29, 30]. Reduction of CRs and ERRs of mebendazole against hookworms stressed the need for a careful understanding of the prevalence of different species of STHs in particular regions before the implementation of a periodic administration of anthelmintic drugs in the deworming campaign. Since the efficacy of the drug started decreasing from studies conducted before 2000 to after 2015, there is a possibility for the emergence of drug-resistant helminthic parasite, and it’s widespread to different areas [31, 32]. This could challenge the promising initiative and hinder the achievement of ambitious goal set by WHO in 2030 [2], particularly given the widespread problem of anthelminthic resistance in livestock as a result of frequent periodic mass treatments [30]. Although there is a trust that, deworming populations once, twice, and even three times a year could not induce a significant amount of drug resistance [33].

However, regardless of the study setting or treatment options, albendazole and mebendazole demonstrated outstanding CRs and ERRs against *A. lumbricoides*. The effectiveness, however, may be jeopardized in cases under high load of parasite (EPG), as this is observed in the sensitivity analysis between parasite EPG and ERR, where negative correlation was found. The combination of Albendazole and Mebendazole with other anthelmintic drugs such as Moxidectin at varied doses (8–24 mg/kg), ivermectin (200ug/kg), diethycarbamazine, and Oxantel pamoate (20 mg/kg), ] substantially enhanced the efficacy of Albendazole and Mebendazole against STHs, except in *A. lumbricoides*. Some studies suggested the use of oxantel pamoate (20 mg/kg single dose) alone or in combination with albendazole (400 mg single dose) or other combinations as an alternative drug or drug regimen for STHs [25, 32]. Thus combining these drugs with others well tolerated and could improve the patient’s CRs of STHs (e.g. pyrantel pamoate and oxantel pamoate), and the use of multiple doses as an option could be a better alternative option which might need further evaluation [34–36]. The drug’s efficacy could be affected by different variables including infection intensity, parasite strain, host factors such as immunity and nutritional condition, and sensitivity of the diagnosis methods [28, 37].

In addition to the inherent effectiveness of a given anthelminthic drug against STHs, other factors such as geographic locations, variations in the study parasite strain and species susceptibility/resistance to anthelmintic drugs, infection intensity (light, mild, and severe) detected at baseline, treatment options, study design, and diagnostic tools might be accountable [38, 39]. Moreover, the meta-regression analysis’s results demonstrated a strong correlation between the ERR of mebendazole and albendazole against *T. trichiura* and the follow-up periods (longer weeks associated with low efficacy). Lack of standard or fixed follow-up weeks for children post-anti-helminthic treatment might affect the accurate efficacy estimations of the drugs. One of the shortcomings of the deworming program strategy is its failure to prevent reinfection after effective deworming [40, 41]. Also, the ERR of these drugs decreased in the years following 2000, signifying that a drug-resistant variant of this parasite may arise and spread throughout the endemic areas since there were no efficacy differences observed in all the WHO deworming program-targeted regions.

### Strengths and Limitations of the Study

The strength of this study is that, to the best of our understanding, this is the most comprehensive systematic review and meta-analysis undertaken on preschool and school-age children targeted by WHO deworming program. A previous systematic review and meta-analysis on the efficacy of anthelmintic drugs against STH infections by Keiser and Utzinger in 2008 [29] was conducted almost long years ago using only 20 randomized controlled trials. Later, in 2017, Moser et al. [30] conducted a review on the efficacy of diverse anthelmintic drugs against STHs in all age populations using 44 studies. In addition, in this study detailed characteristics of the study participants at baseline and post-treatment were systematically extracted to demonstrate the overall prevalence of STHs, intensity of infections, and treatment options, to evaluate the confounders responsible for the observed high heterogeneity, and to show the key characteristics of the study participants. In addition, studies included in the meta-analysis are large enough to draw a conclusive pooled estimation of the drug’s efficacy (ERR and CR) against STHs. Also, articles published on the topic were further manually searched regardless of their years of publication (up to 31st January 2024) and the databases they exist.

One of the major limitations of the study included in this review was the variability of the drug’s efficacy reporting method, where some studies use only CR or ERR while others present it using both (CR and ERR) to show the efficacy of anthelmintic drugs. Consequently, many articles which could be eligible were excluded from the study, or if included they might be excluded from the meta-analysis. Also, the lack of standard or uniform follow-up days post-treatment was prone to the studies irregular reporting, which could potentially affect the accurate efficacy of these drugs. Under some special cases, the follow-up days are extended up to 12 weeks. Hence, for the sake of consistency, for studies containing multiple weeks of efficacy report, the one nearer to the common weeks reported (3 or 4 weeks) in other studies was selected and considered in the data analysis. Another challenge faced during data extraction and analysis was studied with incomplete data such as lack of in-depth data on sample size, and number of positive cases, which estimates standard error of the mean difficult and setting of confidence interval for the meta-analysis impossible, besides difficulty in analyze the overall burden of the STH. As a result, three eligible studies were excluded from the meta-analysis.

## Conclusion

Albendazole and mebendazole demonstrated excellent efficacy against *A. lumbricoides*, irrespective of the study setting, intensity of the infection, geographic areas, and treatment options. While, the estimated efficacy of these two drugs (either alone or in combination with other anthelmintic drugs) against *T. trichiura*, and hookworms in children meets the WHO-recommended cut point for satisfactory efficacy, despite the wide range of ERR recorded in the individual studies included in this analysis. Continuous monitoring of the effectiveness of these drugs is necessary to proactively track the emergence and widespread dissemination of drug-resistant STHs. Clinical research targeting the enhancement of these broad-spectrum anthelminthic drugs’ efficacy against STHs in general, and trichriasis and hookworms in particular are also crucial.

## Electronic Supplementary Material

Below is the link to the electronic supplementary material.

Supplementary Material 1

Supplementary Material 2

Supplementary Material 3

Supplementary Material 4

Supplementary Material 5

Supplementary Material 6

Supplementary Material 7

Supplementary Material 8

Supplementary Material 9

Supplementary Material 10

Supplementary Material 11

## Data Availability

No datasets were generated or analysed during the current study.

## Abbreviations

Alb: Albendazole
CRs: Cure rates
EPG: Egg per gram of stool
ERRs: Egg Reduction rates
Meb: Mebendazole
STH9: Soil transmitted helminthes
SE: Standard error of the mean
*StDev*: Standard deviation
