# Supplementary material for: Efficacy of Albendazole and Mebendazole Against Soil Transmitted Infections among Pre-School and School Age Children: A Systematic Review and Meta-Analysis
Source: J Epidemiol Glob Health. 2024 May 2;14(3):884–904. doi: 10.1007/s44197-024-00231-7 (PMC11442817; doi:10.1007/s44197-024-00231-7)
Supplement: Supplementary file 1 — Supplementary Material 1 [file 44197_2024_231_MOESM1_ESM.docx]

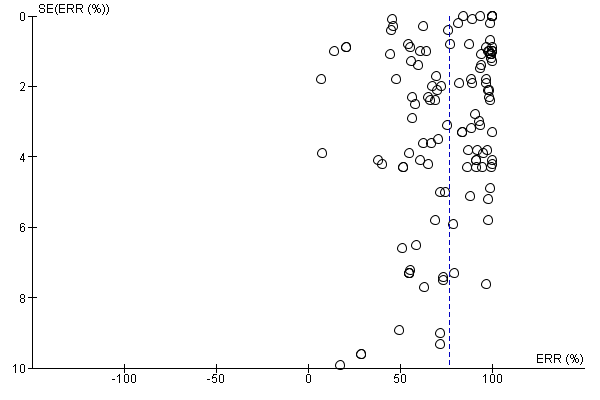


S1 Figure **Funnel plot for publication bias assessment of studies on efficacy of** Albendazole and Mebendazole against *T. trichiura*
