# Supplementary material for: Efficacy of Albendazole and Mebendazole Against Soil Transmitted Infections among Pre-School and School Age Children: A Systematic Review and Meta-Analysis
Source: J Epidemiol Glob Health. 2024 May 2;14(3):884–904. doi: 10.1007/s44197-024-00231-7 (PMC11442817; doi:10.1007/s44197-024-00231-7)
Supplement: Supplementary file 2 — Supplementary Material 2 [file 44197_2024_231_MOESM2_ESM.docx]

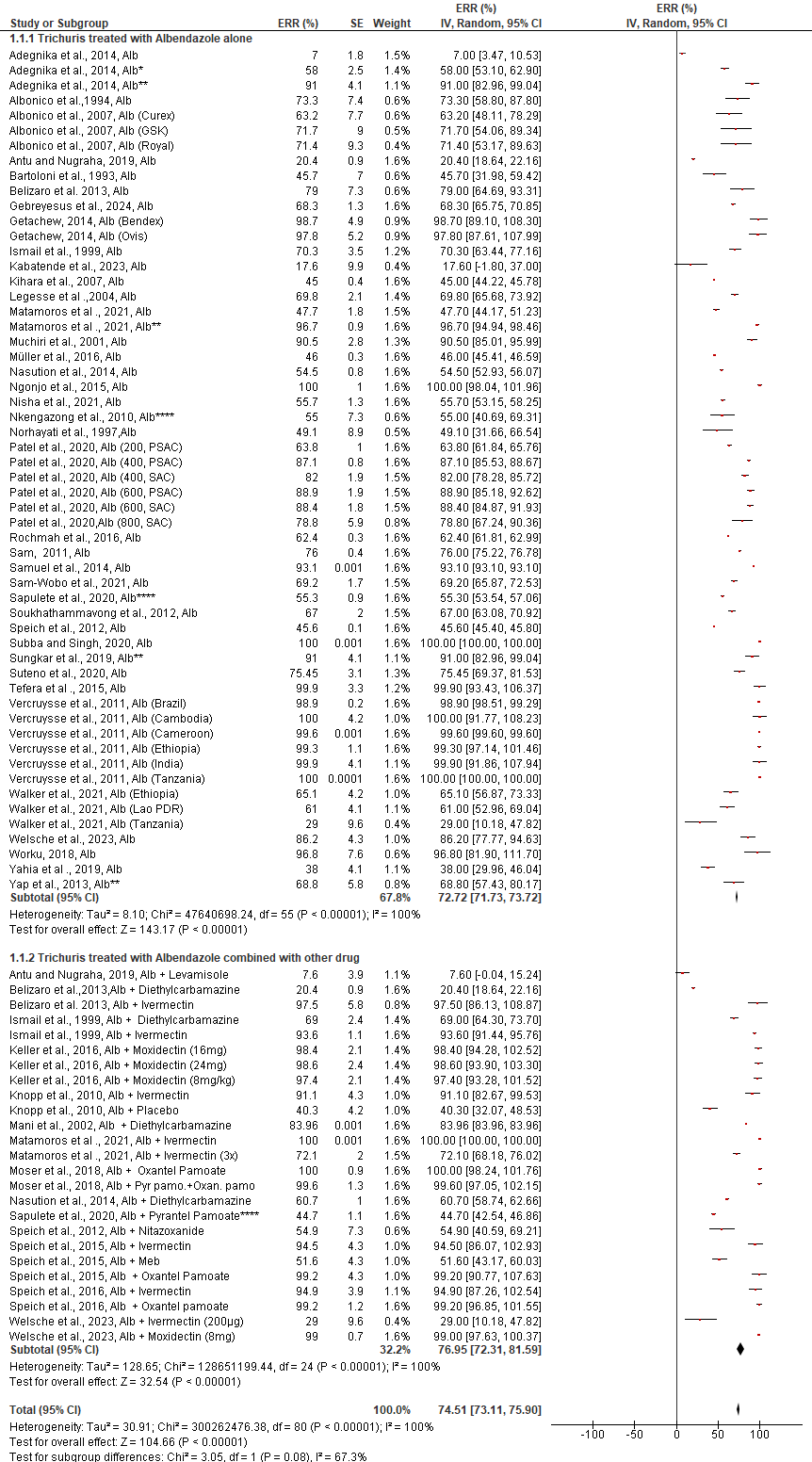


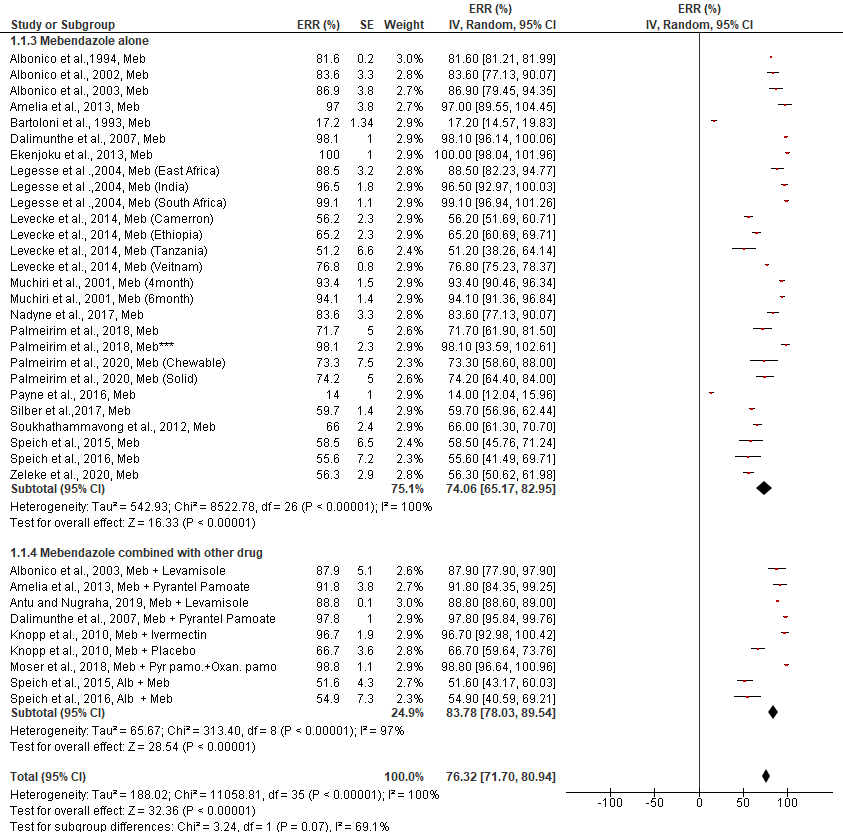


S2 Figure *In vivo* efficacies of Mebendazole and Albendazole against *T. trichiura* in children of preschool and school age in relation to different treatment alternatives. *NB: Studies with asterisk (* = triple (3x) dose, *** = single dose for three days, Alb= Albendazole, Meb = Mebendazole, Pyr.Pamo + Oxan.pamo = Pyrantel pamoate and Oxantel Pamoate*
