# Supplementary material for: Efficacy of Albendazole and Mebendazole Against Soil Transmitted Infections among Pre-School and School Age Children: A Systematic Review and Meta-Analysis
Source: J Epidemiol Glob Health. 2024 May 2;14(3):884–904. doi: 10.1007/s44197-024-00231-7 (PMC11442817; doi:10.1007/s44197-024-00231-7)
Supplement: Supplementary file 4 — Supplementary Material 4 [file 44197_2024_231_MOESM4_ESM.docx]

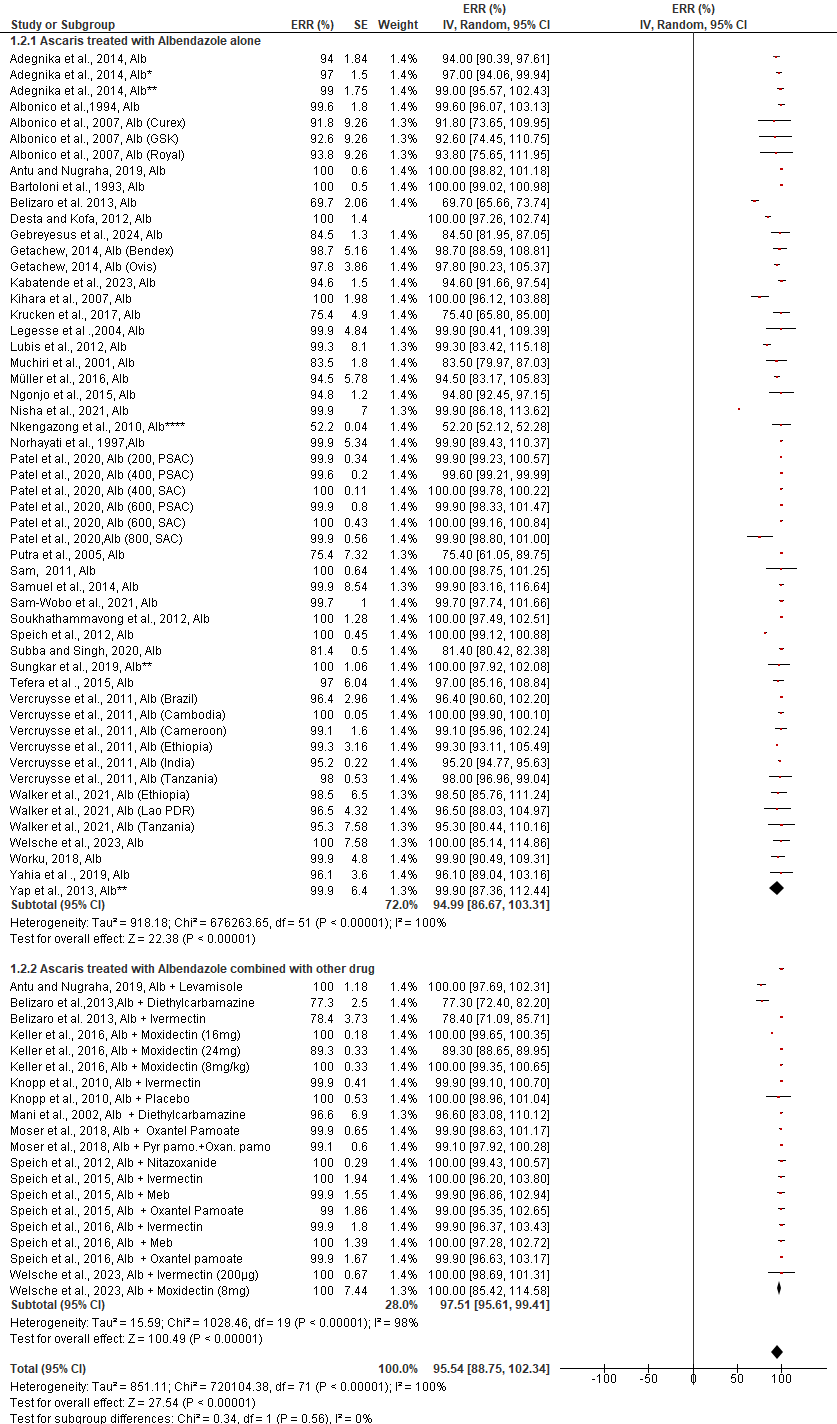


**
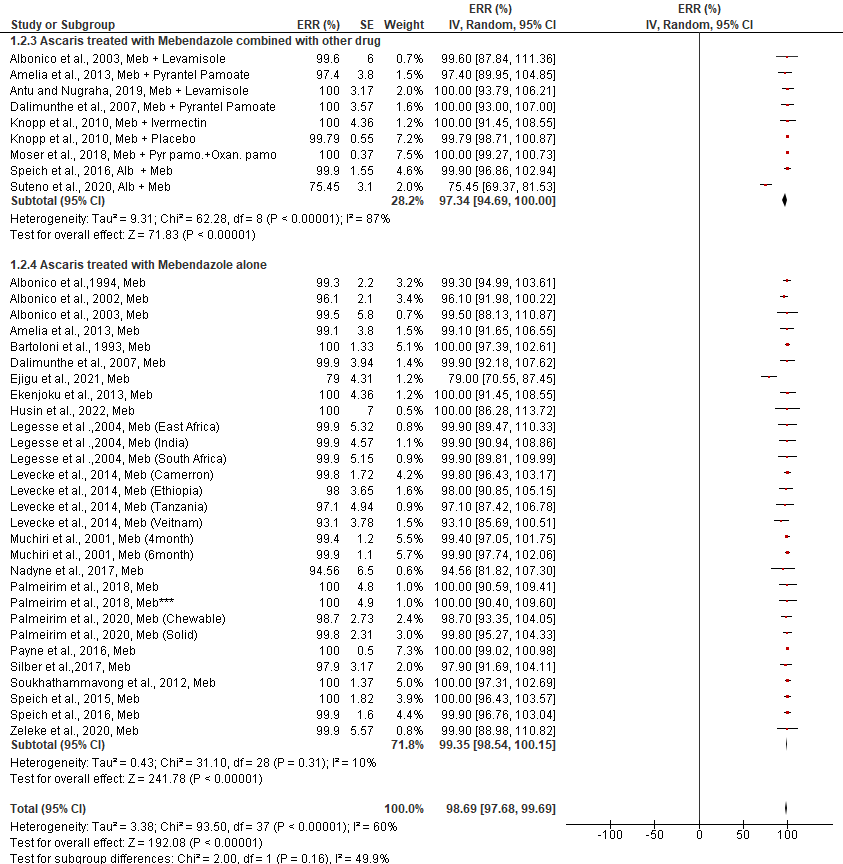
**

S4 Figure Pooled *in vivo* efficacy of Albendazole and Mebendazole against *A. lumbricoides* in pre-school and school age children with respect to different treatment options. *NB: Studies with asterisk (* = double (2x) dose, ** = triple (3x) dose, **** = multiple dose), Alb= Albendazole, Meb = Mebendazole, Pyr.Pamo + Oxan.pamo = Pyrantel pamoate and Oxantel Pamoate*
