# Supplementary material for: Efficacy of Albendazole and Mebendazole Against Soil Transmitted Infections among Pre-School and School Age Children: A Systematic Review and Meta-Analysis
Source: J Epidemiol Glob Health. 2024 May 2;14(3):884–904. doi: 10.1007/s44197-024-00231-7 (PMC11442817; doi:10.1007/s44197-024-00231-7)
Supplement: Supplementary file 5 — Supplementary Material 5 [file 44197_2024_231_MOESM5_ESM.docx]

**
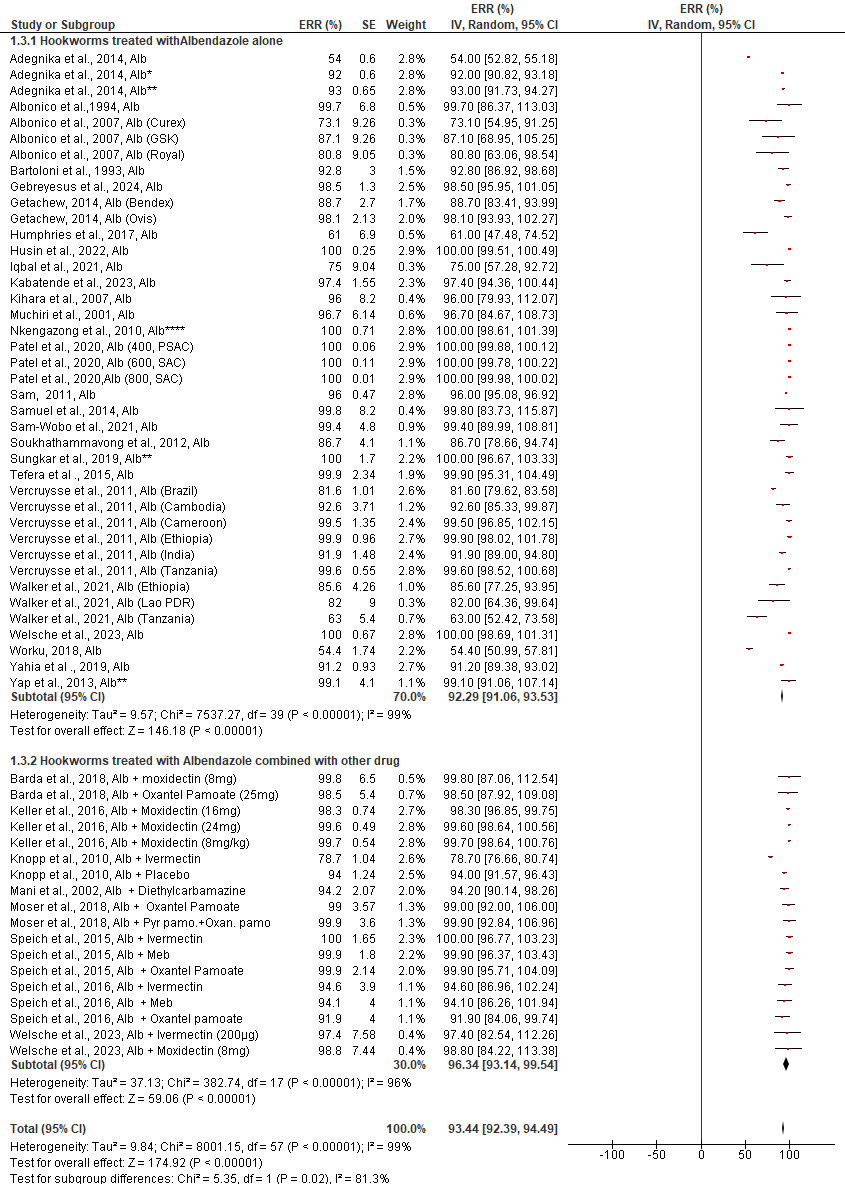
**


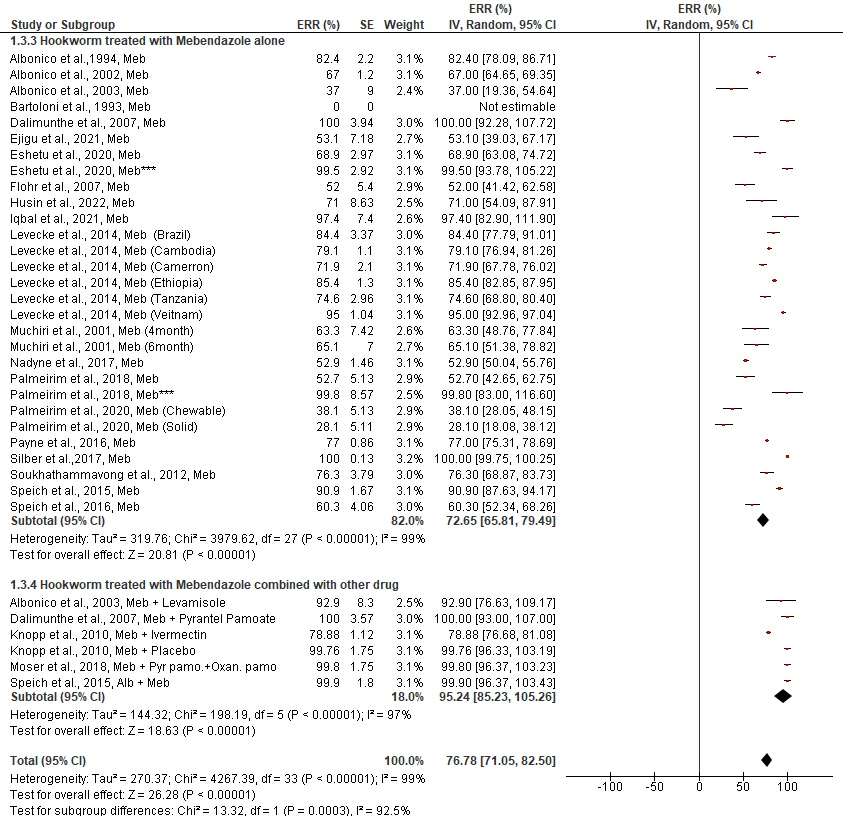


S5 Figure Pooled *in vivo* efficacy of Albendazole and Mebendazole against Hookworm in pre-school and school age children with respect to different treatment options
