# Supplementary figures and images for: Efficacy of Albendazole and Mebendazole Against Soil Transmitted Infections among Pre-School and School Age Children: A Systematic Review and Meta-Analysis
Source: J Epidemiol Glob Health. 2024 May 2;14(3):884–904. doi: 10.1007/s44197-024-00231-7 (PMC11442817; doi:10.1007/s44197-024-00231-7)

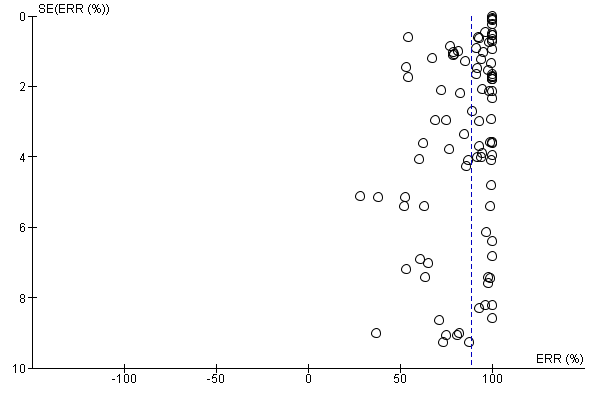


S9 Figure Funnel plot for Mebendazole and Albendazole studies against hookworms

Supplement: Supplementary file 6 — Supplementary Material 6 [file 44197_2024_231_MOESM6_ESM.docx]
