# Supplementary material for: Efficacy of Albendazole and Mebendazole Against Soil Transmitted Infections among Pre-School and School Age Children: A Systematic Review and Meta-Analysis
Source: J Epidemiol Glob Health. 2024 May 2;14(3):884–904. doi: 10.1007/s44197-024-00231-7 (PMC11442817; doi:10.1007/s44197-024-00231-7)
Supplement: Supplementary file 7 — Supplementary Material 7 [file 44197_2024_231_MOESM7_ESM.docx]

S1 Table a summary of search terms/phrases for the databases

| S/N | Database (# Abstract) | Search strategy |
| --- | --- | --- |
| 1 | PubMed (n = 321)  <https://pubmed.ncbi.nlm.nih.gov/> | (((((("efficacies"[All Fields] OR "efficacious"[All Fields] OR "efficaciously"[All Fields] OR "efficaciousness"[All Fields] OR "efficacy"[All Fields]) AND ("albendazole"[MeSH Terms] OR "albendazole"[All Fields] OR "albendazol"[All Fields]) AND ("mebendazole"[MeSH Terms] OR "mebendazole"[All Fields] OR "mebendazol"[All Fields])) OR ("albendazole"[MeSH Terms] OR "albendazole"[All Fields] OR "albendazol"[All Fields]) OR ("mebendazole"[MeSH Terms] OR "mebendazole"[All Fields] OR "mebendazol"[All Fields])) AND (("soil"[MeSH Terms] OR "soil"[All Fields]) AND ("transmit"[All Fields] OR "transmited"[All Fields] OR "transmits"[All Fields] OR "transmitted"[All Fields] OR "transmitting"[All Fields]) AND ("helminthes"[All Fields] OR "helminthic"[All Fields] OR "helminths"[MeSH Terms] OR "helminths"[All Fields] OR "helminth"[All Fields]))) OR ("soc theory health"[Journal] OR "sth"[All Fields]) OR ("ascaris lumbricoides"[MeSH Terms] OR ("ascaris"[All Fields] AND "lumbricoides"[All Fields]) OR "ascaris lumbricoides"[All Fields]) OR ("ancylostomatoidea"[MeSH Terms] OR "ancylostomatoidea"[All Fields] OR "hookworm"[All Fields] OR "hookworms"[All Fields]) OR ("trichuris"[MeSH Terms] OR "trichuris"[All Fields] OR "trichuri"[All Fields]) OR ("ancylostoma"[MeSH Terms] OR "ancylostoma"[All Fields] OR ("ancylostoma"[All Fields] AND "duodenale"[All Fields]) OR "ancylostoma duodenale"[All Fields]) OR ("necatoriasis"[MeSH Terms] OR "necatoriasis"[All Fields])) AND ("child"[MeSH Terms] OR "child"[All Fields] OR "children"[All Fields] OR "child s"[All Fields] OR "children s"[All Fields] OR "childrens"[All Fields] OR "childs"[All Fields])) AND ((medline[Filter]) AND (fha[Filter]) AND (clinicalstudy[Filter] OR clinicaltrial[Filter] OR observationalstudy[Filter] OR randomizedcontrolledtrial[Filter]) AND (humans[Filter]) AND (allchild[Filter])) |
| 2 | Scopus (n= 174)  <https://www.scopus.com> | TITLE-ABS-KEY ( efficacy OR effectiveness AND albendazole OR mebendazole AND soil AND transmitted AND helminths OR sth AND children ) AND ( LIMIT-TO ( EXACTKEYWORD , "human" ) OR LIMIT-TO ( EXACTKEYWORD , "child" ) OR LIMIT-TO ( EXACTKEYWORD , "hookworm infection" ) OR LIMIT-TO ( EXACTKEYWORD , "trichuris trichiura" ) OR LIMIT-TO ( EXACTKEYWORD , "ascaris lumbricoides" ) ) AND ( LIMIT-TO ( DOCTYPE , "ar" ) ) |
| 3 | Embase (n= 431) <https://www.embase.com> | ((('efficacy'/exp OR efficacy) AND ('albendazole'/exp OR albendazole) OR 'mebendazole'/exp OR mebendazole) AND ('soil transmitted helminths' OR (('soil'/exp OR soil) AND transmitted AND ('helminths'/exp OR helminths))) OR 'sth'/exp OR sth) AND ('children'/exp OR children) AND ([controlled clinical trial]/lim OR [randomized controlled trial]/lim) AND ('cross sectional study'/de OR 'human'/de) AND ([child]/lim OR [preschool]/lim OR [school]/lim) AND ('article'/it OR 'article in press'/it) |
| 4 | Directory Online Access Journals (n=34)  <https://doaj.org> | var SEARCH_CONFIGURED_OPTIONS={"query":{"bool":{"must":[{"term":{"_type":"article"}},{"query_string":{"query":"Efficacy of Albendazole OR mebendazole AND soil transmitted infections OR STHs OR children","default_operator":"AND"}}]}},"track_total_hits":true} |
| 5 | Science Direct (n = 106)  [https://www.sciencedirect.com/](https://www.sciencedirect.com) | Efficacy of Albendazole and mebendazole against soil transmitted infections in children AND (research articles) |
| 6 | WHO Clinical Trial.gov. n= 253  <https://classic.clinicals.gov> | Albendazole OR Mebendazole in Title Abstract Keyword AND Soil Transmitted Helminthes OR STHs OR Ascaris OR Hookworm OR Trichusris Title Abstract Keyword AND "Children" in Title Abstract Keyword |
| 7 | African Journals Online (AJOL) n= 120 | Efficacy of Albendazole and mebendazole against soil transmitted infections in children  (Manually searched) |
| 8 | Google scholar (n=930) (<https://scholar.google.com/>) | Efficacy of Albendazole OR mebendazole AND soil transmitted infections OR STHs OR children.  (Manually searched) |
