# Supplementary material for: Efficacy of Albendazole and Mebendazole Against Soil Transmitted Infections among Pre-School and School Age Children: A Systematic Review and Meta-Analysis
Source: J Epidemiol Glob Health. 2024 May 2;14(3):884–904. doi: 10.1007/s44197-024-00231-7 (PMC11442817; doi:10.1007/s44197-024-00231-7)
Supplement: Supplementary file 8 — Supplementary Material 8 [file 44197_2024_231_MOESM8_ESM.docx]

S2 Table Excluded articles and reason for their exclusion of publications on efficacy of Albendazole and Mebendazole against STHs in children

| S/N | Author ID | Title | Journal | Reason for exclusion |
| --- | --- | --- | --- | --- |
| 1 | Ebenezer et al., 2013 | Cluster-randomised trial of the impact of school-based deworming and iron supplementation on the cognitive abilities of schoolchildren in Sri Lanka's plantation sector. | Tropical medicine & international Health | Incomplete data |
|  |  |  |  |  |
| 2 | Farahmandian et al ., 1977 | Comparative studies on the evaluation of the effect of new anthelminthics on various intestinal helminthiasis in Iran. Effects of anthelminthics on intestinal helminthiasis. | Chemotherapy | Irrelevant data |
| 3 | Reynoldson et al., 1988 | Efficacy of albendazole against Giardia and hookworm in a remote Aboriginal community in the north of Western Australia. | ActaTropica | Incomplete data |
| 4 | Penggabean et al., 1988 | Efficacy of albendazole in the treatment of Trichuristrichuria and Giardia intestinalis infection in rural Malay communities. | Medical Journal of Malaysia | Incomplete data |
| 5 | Kabatende et al ., 2022 | Safety of Praziquantel and Albendazole Coadministration for the Control and Elimination of Schistosomiasis and Soil-Transmitted Helminths Among Children in Rwanda: An Active Surveillance Study. | Drug safety | Irrelevant data |
|  |  |  |  |  |
| 6 | Uneke , 2010 | Soil transmitted helminth infections and schistosomiasis in school age children in sub-Saharan Africa: efficacy of chemotherapeutic intervention since World Health Assembly Resolution 2001 | Tanzania Journal of Health Research | Irrelevant data |
|  |  |  |  |  |
| 7 | Rajendran et al., 2018 | Sustainability of soil-transmitted helminth control following a single-dose co-administration of albendazole and diethylcarbamazine. | Transactions of the Royal Society of Tropical Medicine and Hygiene | Incomplete data |
|  |  |  |  |  |
| 8 | Joseph et al., 2015 | The Effect of Deworming on Growth in One- Year-Old Children Living in a Soil-Transmitted Helminth-Endemic Area of Peru: A Randomized Controlled Trial. PL | PLoS Neglected Tropical Disease | Incomplete data |
|  |  |  |  |  |
| 9 | Vlaminck et al., 2019 | Therapeutic efficacy of albendazole against soil-transmitted helminthiasis in children measured by five diagnostic methods. | PLoS Neglected Tropical Disease | Incomplete data |
| 10 | Dissanaike et al., 1978 | A comparative trial of oxantel-pyrantel and mebendazole in multiple helminth infection in school children. | Drug | Incomplete data |
| 11 | Namwanje et al., 2011 | A randomised controlled clinical trial on the safety of co-administration of albendazole, ivermectin and praziquantel in infected schoolchildren in Uganda | Transactions of the Royal Society of Tropical Medicine and Hygiene | Incomplete data |
|  |  |  |  |  |
| 12 | Jinabhai et al., 2001 | A randomized controlled trial of the effect of antihelminthic treatment and micronutrient fortification on health status and school performance of rural primary school children | Annals of Tropical Paediatrics | Incomplete data |
|  |  |  |  |  |
| 13 | Stothard et al., 2009 | A spot-check of the efficacies of albendazole or levamisole, against soil-transmitted helminthiases in young Ungujan children, reveals low frequencies of cure. | Annals of Tropical Medicine & Parasitology | Incomplete data |
|  |  |  |  |  |
| 14 | Echazu et al., 2017 | Albendazole and ivermectin for the control of soil-transmitted helminths in an area with high prevalence of Strongyloidesstercoralis and hookworm in northwestern Argentina: A community-based pragmatic study. | PLoS Neglected Tropical Disease | Incomplete data |
|  |  |  |  |  |
| 15 | Krolewiecki et al., 2022 | An adaptive phase II/III safety and efficacy randomized controlled trial of single day or three-day fixed-dose albendazole-ivermectin co-formulation versus albendazole for the treatment of *Trichuris trichiura* and other STH infections. ALIVE trial protocol. | Gates Open Research | Incomplete data |
|  |  |  |  |  |
| 16 | Levecke et al., 2014 | Assessment of anthelmintic efficacy of mebendazole in school children in six countries where soil-transmitted helminthes are endemic. | PLoS Neglected Tropical Disease | Incomplete data |
|  |  |  |  |  |
| 17 | Belew et al., 2015 | Assessment of Efficacy and Quality of Two Albendazole Brands Commonly Used against Soil- Transmitted Helminthes Infections in School Children in Jimma Town, Ethiopia. | PLoS Neglected Tropical Disease | Incomplete data |
|  |  |  |  |  |
| 18 | Shrestha et al., 2014 | Assessment of Efficacy of Single-Dose Albendazole in Treatment of Intestinal Helminth Parasites in School-Children of Bhaktapur | Journal of Natural History Museum | Incomplete data |
|  |  |  |  |  |
| 19 | Zongo et al., 2016 | Assessment of schistosomiasis and intestinal helminths following mass drug administration in the Centre and Plateau Central regions of Burkina Faso | International Journal of Biological and Chemical Science | Incomplete data |
|  |  |  |  |  |
| 20 | Hadju et al., 1996 | Comparative trials using albendazole and mebendazole in the treatment of soil-transmitted helminths in schoolchildren on Penang, Malaysia. Southeast Asian | Medical Journal of Indonesia | Incomplete data |
|  |  |  |  |  |
| 21 | Sacko et al., 1999 | Comparison of the efficacy of mebendazole, albendazole and pyrantel in treatment of human hookworm infections in the southern region of Mali, West Africa | Transactions of the Royal Society of Tropical Medicine and Hygiene | Incomplete data |
| 22 | Albonico et al., 2012 | Comparison of the Kato-Katz thick smear and McMaster egg counting techniques for monitoring drug efficacy against soil-transmitted helminths in schoolchildren on Pemba Island, Tanzania. | Transactions of the Royal Society of Tropical Medicine and Hygiene | Incomplete data |
|  |  |  |  |  |
| 23 | Olds et al., 1999 | Double-blind placebo-controlled study of concurrent administration of albendazole and praziquantel in schoolchildren with schistosomiasis and geohelminths | The Journal of Infectious Diseases | Incomplete data |
|  |  |  |  |  |
| 24 | Aryadnyani et al., 2019 | Single-dose Albendazole 400 mg Effectiveness in Ascaris lumbricoides and Trichuris trichiura Infections | Asian Journal of Applied Sciences | Incomplete data |
| 25 | Campolina et al., 2015 | Effective anthelmintic therapy of residents living in endemic area of high prevalence for Hookworm and Schistosoma mansoni infections enhances the levels of allergy risk factor anti-Der p1 IgE | Results in Immunology | Irrelevant data |
|  |  |  |  |  |
| 26 | Ngonjo et al., 2015 | Effectiveness of albendazole on soil transmitted nematodes among school going children in kakamega county, kenya | African Journal of Health Sciences | Irrelevant data |
| 27 | Brito et al ., 2016 | Effectiveness of the treatment of helminth infections (S. mansoni, Ancylostomidae, T. trichiura, A. lumbricoides) on hemoglobin concentration in school-children and adolescents. | Journal of Human Nutrition & Food Science | Irrelevant data |
|  |  |  |  |  |
| 28 | Mani et al., 2004 | Effectiveness of two annual, single‐dose mass drug administrations of diethylcarbamazine alone or in combination with albendazole on soil‐transmitted | Tropical Medicine and International Health | Incomplete data |
|  |  | helminthiasis in filariasis elimination programme |  |  |
| 29 | Theriault et al., 2014 | Efficacy and reinfection with soil-transmitted helminths 18-weeks post-treatment with albendazole-ivermectin, albendazole-mebendazole, albendazole-oxantel pamoate and mebendazole | PLoS Neglected Tropical Disease | Incomplete data |
|  |  |  |  |  |
| 30 | Olsen , 2007 | Efficacy and safety of drug combinations in the treatment of schistosomiasis, soil-transmitted helminthiasis, lymphatic filariasis and onchocerciasis | Transactions of the Royal Society of Tropical Medicine and Hygiene | Irrelevant data |
|  |  |  |  |  |
| 31 | Patel et al., 2019 | Efficacy and safety of ivermectin and albendazole co- administration in school-aged children and adults infected with Trichuristrichiura: study protocol for a multi-country randomized controlled double-blind trial. | BMC Infectious Diseases | Incomplete data |
|  |  |  |  |  |
| 32 | Kshirsagar et al., 2017 | Efficacy and tolerability of treatment with single doses of diethylcarbamazine (DEC) and DEC plus albendazole (ABZ) for three consecutive years in lymphatic filariasis: a field study in India | Parasitology Research | Irrelevant data |
|  |  |  |  |  |
| 33 | Edelduok et al., 2013 | Efficacy of a single dose albendazole chemotherapy on human intestinal helminthiasis among school children in selected rural tropical communities | Annals of Tropical Medicine and Public Health | Incomplete data |
|  |  |  |  |  |
| 34 | Nontasut et al., 2000 | Efficacy Of Multiple Dose Mebendazole Against Trichuriasis In Thai And Karen Patients | Southeast Asian J Trop Med Public Health | Incomplete data |
| 35 | Mekonnen et al., 2013 | Efficacy of different albendazole and mebendazole regimens against heavy-intensity Trichuristrichiura infections in school children, Jimma Town, Ethiopia | Pathogens and Global Health | Incomplete data |
| 36 | Namwanje et al ., 2010 | Efficacy of single and double doses of albendazole and mebendazole alone and in combination in the treatment of Trichuristrichiura in school-age children in Uganda. |  | Incomplete data |
|  |  |  |  |  |
| 37 | Hailu et al., 2018 | Efficacy of single dose albendazole and praziquantel drugs among helminth-infected school children at Rural Bahir Dar, northwest Ethiopia. | Tropical Doctor | Incomplete data |
|  |  |  |  |  |
| 38 | Bockarie et al., 2007 | Efficacy of single-dose diethylcarbamazine compared with diethylcarbamazine combined with albendazole against Wuchereriabancrofti infection in Papua New Guinea | The American Society of Tropical Medicine and Hygiene | Incomplete data |
| 39 | Dahesh, 2017 | Evaluation of a deworming campaign by albendazole during 2016 in a rural area of Giza Governorate, Egypt | Parasitologists United Journal | Incomplete data |
| 40 | Ehrhardt et al., 2006 | Evaluation of coverage of deworming interventions in Vietnam. Southeast Asian | Southeast Asian Journal of Tropical Medicine and Public Health | Irrelevant data |
|  |  |  |  |  |
| 41 | Walker et al., 2021 | Individual responses to a single oral dose of albendazole indicate reduced efficacy against soil-transmitted helminths in an area with high drug pressure | PLOS Neglected Tropical Diseases | Incomplete data |
| 42 | Gichuki et al., 2019 | Long term school based deworming against soil-transmitted helminths also benefits the untreated adult population: results from a community-wide cross sectional survey | Journal of Tropical Medicine | Incomplete data |
| 43 | Sanza et al., 2013 | Monitoring the impact of a mebendazole mass drug administration initiative for soil-transmitted helminthiasis (STH) control in the Western Visayas Region of the Philippines from 2007 through 2011. | ActaTropica | Irrelevant data |
|  |  |  |  |  |
| 44 | Friis et al., 2003 | Effects on haemoglobin of multi-micronutrient supplementation and multi-helminth chemotherapy: a randomized, controlled trial in Kenyan school children | European Journal of Clinical Nutrition | Irrelevant data |
|  |  |  |  |  |
| 45 | Olds, 1994 | Phase II clinical study on safety and efficacy of concurrent administration of Albendazole and Praziquantel in school‐aged children with schistosomiasis and geohelminth infections | Tropical Disease Research | Incomplete data |
|  |  |  |  |  |
| 46 | Bundy et al., 1985 | Population dynamics and chemotherapeutic control of Trichuristrichiura infection of children in Jamaica and St. Lucia | Transactions of the Royal Society of Tropical Medicine and Hygiene | Irrelevant data |
| 47 | Cedric et al., 2020 | Prevalence of Geo-Helminths and Evalua-tion of Single Dose of Albendazole (400 mg) among School Children in Poumougne, Western Region, Cameroon | International Journal of | Incomplete data |
|  |  |  | Tropical Diseases |  |
| 48 | Cabada et al., 2014 | Prevalence of soil-transmitted helminths after mass albendazole administration in an indigenous community of the Manu jungle in Peru | Pathogens and Global Health | Incomplete data |
|  |  |  |  |  |
| 49 | Stothard et al., 2014 | Preventive chemotherapy for schistosomiasis and soil-transmitted helminthiasis by cotreatment with praziquantel and albendazole | Journal of Clinical Investigation | Irrelevant data |
|  |  |  |  |  |
| 50 | Addis et al., 1997 | Randomised placebo-controlled comparison of ivermectin and albendazole alone and in combination for Wuchereria bancrofti microfilaraemia in Haitian children | The Lancet | Irrelevant data |
|  |  |  |  |  |
| 51 | Krucken et al., 2017 | Reduced efficacy of albendazole against Ascaris lumbricoides in Rwandan school children | International Journal for Parasitology: Drugs and Drug Resistance | Incomplete data |
| 52 | Muslim and Lim | Higher efficacy of a single dosage albendazole and different soil-transmitted helminths re-infection profiles amongst indigenous Negritos from inland jungle versus those in resettlement at town peripheries | Tropical Biomedicine | Irrelevant data |
|  |  |  |  |  |
|  | 2022 |  |  |  |
| 53 | Curico et al | Resistance to single dose albendazole and reinfection with intestinal helminths among children ages 2 to 11 years from the Peruvian Amazon region: a study protocol. | BMC Infectious Diseases | Incomplete data |
|  |  |  |  |  |
|  | 128 |  |  |  |
| 54 | Colella et al., 2022 | Risk profiling and efficacy of albendazole against the hookworms Necatoramericanus and Ancylostomaceylanicum in Cambodia to support control programs in Southeast Asia and the Western Pacific, | The Lancet Regional Health - Western Pacific | Incomplete data |
|  |  |  |  |  |
| 55 | Scherrer et al., 2009 | Sequential analysis of helminth egg output in human stool samples following albendazole and praziquantel administration | ActaTropica | Incomplete data |
| 56 | Cleary et al., 2007 | Single low-dose mebendazole administered quarterly for Ascaris treatment | The American Journal of the Medical Sciences | Incomplete data |
| 57 | Annisa et al., | The Effect of Single-Dose Albendazole on the Prevalence of SoilTransmitted Helminth Infections and Nutritional Status of Children in Perokonda Village, Southwest Sumba | eJournalKedokteran Indonesia | Incomplete data |
|  |  |  |  |  |
|  | 2017 |  |  |  |
| 58 | Patel et al ., 2021 | Efficacy and Safety of Albendazole in Hookworm-infected Preschool-aged Children, School-aged Children, and Adults in Côte d'Ivoire: A Phase 2 Randomized, Controlled Dose-finding Trial. | Clinical Infectious Diseases | Incomplete data |
| 59 | Maipanich et al., 1997 | Effect of Albendazole and Mebendazole on soil transmitted helmenth eggs | Southeast Asian Journal of Tropical Medicine and Public Health | Incomplete data |
|  |  |  |  |  |
| 60 | Tee et al., 2022 | Efficacy of triple dose albendazole treatment for soil-transmitted helminth infections | PLOS ONE | Incomplete data |
| 61 | Hurlimann et al., 2022 | Efficacy and safety of co-administered ivermectin and albendazole in school-aged children and adults infected with Trichuristrichiura in Côte d'Ivoire, Laos, and Pemba Island, Tanzania: a double-blind, parallel-group, phase 3, randomised controlled trial, | Lancet Infectious Diseases | Incomplete data |
| 62 | [Albonico  et al. 1994](https://pubmed.ncbi.nlm.nih.gov/?size=200&term=Albonico+M&cauthor_id=7941002) | Efficacy of a single dose of mebendazole on prevalence and intensity of soil-transmitted nematodes in Zanzibar | Trop Geogr Med | Incomplete data |
|  |  |  |  |  |
| 63 | Sheild et al., 1984 | Reinfection with intestinal helminths after treatment with mebendazole and fluctuations in individual Ascarislumbricoides infections with time | Papua and New Guinea Medical Journal | Incomplete data |
|  |  |  |  |  |
|  |  |  |  |  |
| 64 | Legesse et al., 2002 | Efficacy of alebendazole and mebendazole in the treatment of Ascaris and Trichuris infections. | Ethiopian Medical Journal | Incomplete data |
|  |  |  |  |  |
| 65 | Ismail, 1999 | Comparative efficacy of single dose anthelmintics in relation to intensity of geohelminth infections. | The Ceylon Medical Journal | Incomplete data |
|  |  |  |  |  |
| 66 | Admas et al., 2004 | Efficacy of albendazole against the whipworm Trichuris trichiura — a randomised, controlled tria | South African medical journal | Incomplete data |
| 67 | Steinmann et al ., 2011 | Efficacy of Single-Dose and Triple-Dose Albendazole and Mebendazole against Soil-Transmitted Helminths and ***Taenia*** spp.: A Randomized Controlled Trial | PLoS One | Incomplete data |
| 68 | Pambe et al., 1989 | A study of the efficacy and safety of albendazole (Zentel) in the treatment of intestinal helmenthiasis in Kenyan children less than 2 years of age. | East African Medical Journal | Incomplete data |
|  |  |  |  |  |
|  |  |  |  |  |
| 69 | Belew et al., | Assessment of Efficacy and Quality of Two Albendazole Brands Commonly Used against Soil-Transmitted Helminth Infections in School Children in Jimma Town, Ethiopia Sileshi | PLOS Neglected Tropical Diseases | Irrelevant data |
| 70 | Edelduok et al., 2013 | Efficacy of a single dose albendazole chemotherapy on human intestinal helminthiasis among school children in selected rural tropical communities | Annals of Tropical Medicine and Public Health | Incomplete data |
| 71 | Wijaya et al., 2020 | The Effect of Albendazole Administration and Iron-Folic Acid Supplementation on Hemoglobin Level in Primary Schoolchildren with Soil-transmitted Helminth Infections | Journal of Medical Sciences | Incomplete data |
| 72 | Olsen et al., 2009 | Albendazole and mebendazole have low efficacy against Trichuris trichiura in school-age children in Kabale District, Uganda | Transactions of the Royal Society of Tropical Medicine and Hygiene | Incomplete data |
| 73 | Sarmah,1988 | A randomized controlled trial of pyrantel and mebendazole in children with enterobiasis and concomitant ascariasis. | Indian Pediatrics Journal | Inaccessible full text |
|  |  |  |  |  |
| 74 | Pambe et al., 1989 | A study of the efficacy and safety of albendazole (Zentel) in the treatment of intestinal helmenthiasis in Kenyan children less than 2 years of age. | East African Medical Journal | Inaccessible full text |
| 75 | Legesse et al., 2002 | Efficacy of alebendazole and mebendazole in the treatment of Ascaris and Trichuris infections. | Ethiopian Medical Journal | Inaccessible full text |
| 76 | Musgrave et al., 1979 | Evaluation of a new antihelminthic for trichuriasis, hookworm, and stronglyloidiasis | The Medical Journal of Austrilia | Inaccessible full text |
| 77 | Adugna et al.,2007 | Efficacy of mebendazole and albendazole for Ascarislumbricoides and hookworm infections in an area with long time exposure for anti-helminthes, Northwest Ethiopia. Ethiopia | Ethiopian Medical Journal | Inaccessible full text |
| 78 | Zhang et al.,1998 | Field trials on the efficacy of albendazole composite against intestinal nematodiasis | Chinese Journal of Parasitology & Parasitic Diseases | Inaccessible full text |
|  |  |  |  |  |
| 79 | Wei et al.,1982 | Helminth and pinworm control for primary school and kindergarten children by periodical mass-examination and treatment in Suan-Yuan District, Taipei (author's transl) | Chinese Journal of Microbiology and Immunology | Inaccessible full text |
|  |  |  |  |  |
| 80 | Fierlafijn and Vanparijs, 1973 | Mebendazole in enterobiasis: a placebo-- controlled trial in a paediatric community | Tropical and Geographical Medicine | Inaccessible full text |
