# Supplementary material for: Efficacy of Albendazole and Mebendazole Against Soil Transmitted Infections among Pre-School and School Age Children: A Systematic Review and Meta-Analysis
Source: J Epidemiol Glob Health. 2024 May 2;14(3):884–904. doi: 10.1007/s44197-024-00231-7 (PMC11442817; doi:10.1007/s44197-024-00231-7)
Supplement: Supplementary file 9 — Supplementary Material 9 [file 44197_2024_231_MOESM9_ESM.docx]

S3 (a) Table. Risk of bias for randomized clinical trials (ROB-2) on the efficacy of Albendazole and Mebendazole against STHs in children

|  | Critical appraisal item (**low risk**, **high risk, and unclear risk of bias**) | | | | | | |
| --- | --- | --- | --- | --- | --- | --- | --- |
| Authors ID | Random sequence generation (e.g. computer based randomization) | Allocation concealment (selection bias) (participants are blinded to the data collectors) sealed postal allocation | Blinding of participants and person (performance bias) | Blinding of outcome assessment (detection bias) | Incomplete outcome bias/attrition bias ( | Reporting bias | Other bias |
| Adegnika et al., 2014 | Low risk | Low risk | Low risk | Low risk | Low risk | Low risk | Low risk |
| Albonico et al.,1994 | Low risk | Low risk | Low risk | Low risk | Low risk | Low risk | Low risk |
| Albonico et al., 2002 | Low risk | Low risk | Low risk | Low risk | Low risk | Low risk | Low risk |
| Albonico et al., 2007 | Low risk | Low risk | Low risk | Low risk | Low risk | Low risk | Low risk |
| Albonico et al., 2003 | Low risk | Low risk | Low risk | Low risk | high risk | Low risk | Low risk |
| Amelia et al., 2013 | Low risk | high risk | unclear risk | high risk | unclear risk | Low risk | Low risk |
| Antu and Nugraha, 2019 | high risk | high risk | Low risk | high risk | unclear risk | Low risk | Low risk |
| Barda et al., 2018 | Low risk | Low risk | Low risk | unclear risk | Low risk | Low risk | Low risk |
| Bartoloni et al., 1993 | Low risk | high risk | Low risk | high risk | unclear risk | Low risk | Low risk |
| Belizario et al., 2003 | unclear risk | Low risk | Low risk | Low risk | unclear risk | Low risk | Low risk |
| Dalimunthe et al., 2007 | Low risk | unclear risk | Low risk | high risk | unclear risk | Low risk | Low risk |
| Ekenjoku et al., 2013 | Low risk | high risk | Low risk | high risk | low risk | high risk | high risk |
| Eshetu et al., 2020 | Low risk | Low risk | Low risk | Low risk | Low risk | Low risk | Low risk |
| Flohr et al., 2007 | Low risk | Low risk | Low risk | Low risk | Low risk | Low risk | Low risk |
| Getachew, 2014 | high risk | high risk | Low risk | Low risk | unclear risk | Low risk | Low risk |
| Husin et al., 2022 | high risk | high risk | high risk | high risk | high risk | high risk | Low risk |
| Ismail et al., 1999 | Low risk | high risk | Low risk | high risk | unclear risk | Low risk | Low risk |
| Keller et al., 2016 | Low risk | Low risk | Low risk | unclear risk | Low risk | Low risk | Low risk |
| Knopp et al., 2010 | Low risk | Low risk | Low risk | Low risk | Low risk | Low risk | Low risk |
| Levecke et al., 2014 | high risk | high risk | Low risk | unclear risk | Low risk | Low risk | Low risk |
| Legesse et al .,2004 | Low risk | high risk | Low risk | high risk | Low risk | Low risk | Low risk |
| Matamoros et al ., 2021 | Low risk | Low risk | Low risk | high risk | high risk | Low risk | Low risk |
| Moser et al., 2018 | Low risk | Low risk | Low risk | Low risk | Low risk | Low risk | Low risk |
| Muchiri et al., 2001 | Low risk | Low risk | Low risk | Low risk | Low risk | Low risk | Low risk |
| Patel et al., 2020 | Low risk | Low risk | Low risk | Low risk | Low risk | Low risk | Low risk |
| Palmeirim et al., 2018 | Low risk | Low risk | Low risk | Low risk | Low risk | Low risk | Low risk |
| Palmeirim et al., 2020 | Low risk | Low risk | Low risk | Low risk | Low risk | Low risk | Low risk |
| Putra et al., 2005 | Low risk | High risk | Low risk | High risk | Low risk | Low risk | high risk |
| Sapulete et al., 2020 | Low risk | Low risk | Low risk | Low risk | Low risk | Low risk | Low risk |
| Silber et al.,2017 | Low risk | Low risk | Low risk | Low risk | Low risk | Low risk | Low risk |
| Soukhathammavong et al., 2012 | Low risk | Low risk | Low risk | Low risk | Low risk | Low risk | Low risk |
| Speich et al., 2012 | Low risk | Low risk | Low risk | Low risk | Low risk | Low risk | Low risk |
| Speich et al., 2015 | Low risk | Low risk | Low risk | Low risk | Low risk | Low risk | Low risk |
| Speich et al., 2016 | Low risk | Low risk | Low risk | Low risk | Low risk | Low risk | Low risk |
| Suteno et al., 2020 | Low risk | High risk | Low risk | High risk | Low risk | Low risk | Low risk |
| Tefera et al., 2015 | Low risk | High risk | Low risk | Low risk | Low risk | Low risk | Low risk |
| Vercruysse et al., 2011 | Unclear risk | Unclear risk | Low risk | High risk | Low risk | Low risk | Low risk |
| Walker et al., 2021 | High risk | High risk | Low risk | Unclear risk | Unclear risk | Low risk | Low risk |
| Welsche et al., 2023 | Low risk | Low risk | Low risk | Low risk | Low risk | Low risk | Low risk |
| Yap et al., 2013 | Low risk | Low risk | Low risk | Low risk | Low risk | Low risk | Low risk |

S3 (b) Table. Risk bias for the Non-randomized Studies – of Interventions (ROBINS-I) on the efficacy of Albendazole and Mebendazole against STHs in children

| Authors ID | Critical appraisal item (**low risk**, moderate **risk, serious, and critical risk of bias**) | | | | | | |
| --- | --- | --- | --- | --- | --- | --- | --- |
|  | Bias due to **confounding** | Bias in selection of participants into the study | Bias in classification of interventions | Bias due to deviation from intended intervention | Bias due to **missing data** | Bias in measurement of the outcome | Bias in selection of report results |
| Ejigu et al., 2021 | Moderate risk | Low risk | Low risk | Low risk | Low risk | Moderate risk | Low risk |
| Gebreyesus et al., 2024 | Low risk | Moderate risk | Low risk | Low risk | Low risk | Moderate risk | Low risk |
| Humpharis et al., 2017 | Low risk | Serious risk | Low risk | Moderate risk | Low risk | Low risk | Low risk |
| Iqbal et al., 2021 | Moderate risk | Moderate risk | Low risk | Low risk | Moderate risk | Serious risk | Serious risk |
| Kabatende et al., 2023 | Low risk | Low risk | Low risk | Low risk | Low risk | Low risk | Low risk |
| Kihara et al., 2007 | Low risk | Moderate risk | Low risk | Low risk | Low risk | Moderate risk | Low risk |
| Krücken et al., 2017 | Low risk | Low risk | Low risk | Low risk | Low risk | Low risk | Low risk |
| Levecke et al., 2014 | Moderate risk | Low risk | Low risk | Low risk | Low risk | Low risk | Low risk |
| Lubis et al., 2012 | Moderate risk | Low risk | Low risk | Low risk | Low risk | Low risk | Moderate risk |
| Müller et al., 2016 | Moderate risk | Moderate risk | Low risk | Low risk | Low risk | Moderate risk | Low risk |
| Mani et al., 2002 | Moderate risk | Low risk | Low risk | Low risk | Low risk | Low risk | Low risk |
| Nadyne et al., 2017 | Low risk | Low risk | Low risk | Low risk | Low risk | Low risk | Low risk |
| Nisha et al., 2021 | Moderate risk | Moderate risk | Low risk | Low risk | Low risk | Moderate risk | Low risk |
| Nkengazong et al., 2010 | Moderate risk | Serious risk | Low risk | Low risk | Low risk | Moderate risk | Low risk |
| Ngonjo et al., 2015 | Low risk | Low risk | Low risk | Low risk | Low risk | Low risk | Low risk |
| Norhayati et al., 1997 | Moderate risk | Moderate risk | Low risk | Serious risk | Low risk | Moderate risk | Low risk |
| Payne et al., 2016 | Moderate risk | Low risk | Low risk | Low risk | Low risk | Moderate risk | Low risk |
| Rochmah et al., 2016 | Low risk | Moderate risk | Low risk | Low risk | Low risk | Serious risk | Low risk |
| Sam, 2011 | Moderate risk | Low risk | Low risk | Low risk | Low risk | Moderate risk | Low risk |
| Samuel et al., 2014 | Low risk | Moderate risk | Low risk | Low risk | Serious risk | Moderate risk | Low risk |
| Sam-Wobo et al., 2021 | Moderate risk | Serious risk | Low risk | Low risk | Low risk | Moderate risk | Low risk |
| Subba and Singh, 2020 | Moderate risk | Low risk | Low risk | Moderate risk | Low risk | Serious risk | Low risk |
| Sungkar et al., 2019 | Low risk | Low risk | Low risk | Low risk | Moderate risk | Moderate risk | Low risk |
| Worku, 2018 | Low risk | Low risk | Low risk | Low risk | Low risk | Moderate risk | Low risk |
| Yahia et al ., 2019 | Low risk | Moderate risk | Low risk | Low risk | Low risk | Low risk | Low risk |
| Zeleke et al., 2020 | Moderate risk | Low risk | Low risk | Low risk | Low risk | Moderate risk | Low risk |
