# Supplementary material for: Efficacy of Albendazole and Mebendazole Against Soil Transmitted Infections among Pre-School and School Age Children: A Systematic Review and Meta-Analysis
Source: J Epidemiol Glob Health. 2024 May 2;14(3):884–904. doi: 10.1007/s44197-024-00231-7 (PMC11442817; doi:10.1007/s44197-024-00231-7)
Supplement: Supplementary file 10 — Supplementary Material 10 [file 44197_2024_231_MOESM10_ESM.docx]

S5 Table characteristics of studies included in the Study of Albendazole and Mebendazole efficacy against STHs in Children

| **Authors ID** | **Region/** | **Year** | **Treatment type** | **Study design** | | | | **Key characteristics** | | | | **Diagnosis** | **Follow-up weeks** | ***Trichuris trichura*** | | | | | |  | | **Ascaris lumbricoides** | | | | | |  | | **Hookworm** | | | | | |  | |
| --- | --- | --- | --- | --- | --- | --- | --- | --- | --- | --- | --- | --- | --- | --- | --- | --- | --- | --- | --- | --- | --- | --- | --- | --- | --- | --- | --- | --- | --- | --- | --- | --- | --- | --- | --- | --- | --- |
|  |  |  |  |  |  |  |  |  |  |  |  |  |  | **# infection** | **Infection intensity** | | | **EPG^a^** | **EPG^b^** | **Treatment outcomes** | | **# infection** | **Infection intensity** | | | **EPG^a^** | **EPG^b^** | **Treatment outcomes** | | **# infection** | **Infection intensity** | | | **EPG^a^** | **EPG^b^** | **Treatment outcomes** | |
|  |  |  |  | **Setting** | **Study type** | **Sample size** | **STH +ve** | **Age (year)** | | **Sex** | |  |  |  |  |  |  |  |  |  |  |  |  |  |  |  |  |  |  |  |  |  |  |  |  |  |  |
|  |  |  |  |  |  |  |  | **Range** | **mean** | **M** | **F** |  |  |  | **L** | **M** | **H** |  |  | **ERR** | **CR** |  | **L** | **M** | **H** |  |  | **ERR** | **CR** |  | **L** | **M** | **H** |  |  | **ERR** | **CR** |
| Adegnika et al., 2014 | Africa/ Gabon | 2010- 2011 | Alb (400mg) | Sch.Children | RCT | 436 | 266 | 4-14 | 9.1 | 29 | 29 | Kato-Katz | 6 | 50 | ND | ND | ND | 700 | 352 | 7 | 40 | 39 | ND | ND | ND | 4794 | 188 | 94 | 85 | 13 | ND | ND | ND | 317 | 360 | 54 | 54 |
|  |  |  | Alb (2X dose) |  |  |  |  |  | 8.9 | 26 | 25 |  |  | 39 | ND | ND | ND | 464 | 260 | 58 | 67 | 32 | ND | ND | ND | 5409 | 1136 | 97 | 91 | 13 | ND | ND | ND | 213 | 80 | 92 | 92 |
|  |  |  | Alb (3 X dose) |  |  |  |  |  | 8.2 | 6 | 58 |  |  | 53 | ND | ND | ND | 415 | 280 | 91 | 83 | 37 | ND | ND | ND | 4734 | 180 | 99 | 91 | 15 | ND | ND | ND | 305 | 80 | 93 | 93 |
| Albonico et al.,1994 | Africa/ Tanzania | 1992-1993 | Alb (400 mg) | Sch.Children | RCT | 2648 | 402 | 6-12 | ND | ND | ND | Kato-Katz | 3 | 1174 | ND | ND | ND | 655 | 174 | 73.3 | 10.5 | 1174 | ND | ND | ND | 239 | 0.05 | 99.6 | 98.9 | 1174 | ND | ND | ND | 391 | 8 | 99.7 | 56.8 |
|  |  |  | Meb (500 mg) |  |  |  |  |  |  |  |  |  |  | 1120 | ND | ND | ND | 658 | 120 | 81.6 | 14.2 | 1120 | ND | ND | ND | 164 | 0.08 | 99.3 | 97.8 | 1120 | ND | ND | ND | 423 | 73 | 82.4 | 22.4 |
| Albonico et al., 2002 | Afri/Zanzibar | 2000 | Meb (500mg/kg)1X | Sch.Children | RCT | 1329 | 448 | 8-13 | 9.5 |  |  | Kato-Katz | 3 | 448 | ND | ND | ND | 257 | 41 | 83.6 | 25.2 |  | 448 | ND | ND | 5 | 0.1 | 96.1 | 98 |  | 448 | ND | ND | 588 | 197 | 67 | 13.2 |
| Albonico et al., 2007 | Asia/ Nepal |  | Alb (400mg) GSK | Community | RCT | 2140 | 1506 | 6-14 | 11.6 | 682 | 595 | Kato-Katz | 3 | 429 | ND | ND | ND | 79 | 22 | 71.7 | 28.6 | 429 | ND | ND | ND | 13 | 0 | 92.6 | 97 | 429 | ND | ND | ND | 13 | 0.9 | 87.1 | 74.3 |
|  |  |  | Alb (400mg) Royal drug |  |  |  |  |  |  |  |  |  |  | 419 | ND | ND | ND | 75 | 21 | 71.4 | 26.6 | 419 | ND | ND | ND | 17 | 0.1 | 93.8 | 95 | 419 | ND | ND | ND | 15 | 2 | 80.8 | 53.3 |
|  |  |  | Alb (400mg) Curex |  |  |  |  |  |  |  |  |  |  | 429 | ND | ND | ND | 60 | 21 | 63.2 | 28 | 429 | ND | ND | ND | 19 | 0.6 | 91.9 | 82.6 | 429 | ND | ND | ND | 12 | 2.4 | 73.1 | 50.7 |
| Albonico et al., 2003 | Africa/ Tanzania | 1999 | Meb (500mg) | Sch.Children | RCT | 904 | 458 | 6-14 | 11.5 | 138 | 147 | Kato-Katz | 3 | 206 | 108 | 93 | 5 | 534 | 69 | 86.9 | 22.8 | 175 | 117 | 25 | 33 | 264 | 0.4 | 99.5 | 93.8 | 271 | 207 | 33 | 31 | 674 | 619 | 37 | 4.4 |
|  |  |  | Meb (500mg) + levamisole (40/80mg) |  |  |  |  |  | 11.7 | 123 | 163 |  |  | 268 | 207 | 60 | 1 | 776 | 94 | 87.9 | 17.1 | 180 | 53 | 17 | 10 | 248 | 0.1 | 99.6 | 100 | 272 | 241 | 17 | 14 | 604 | 42 | 92.9 | 26 |
| Amelia et al., 2013 | Asia/Indonesia | 2009 | Meb (500mg/kg)plus pyrantel pamoate 10 mg/kg | Sch.Children | RCT | 288 | 130 | 9 | ND | ND | ND | Kato-Katz | 4 | 65 | ND | ND | ND | 118.1 | 3.6 | 91.8 | 89.5 | ND | ND | ND | 1012 | 9.1 | ND | 97.4 | 98.5 | ND | ND | ND | ND | ND | ND | ND | ND |
|  |  |  | Meb (500mg/kg)1x |  |  |  |  | 9.6 | ND | ND | ND |  |  | 65 | ND | ND | ND | 131.9 | 10.8 | 97 | 78.5 | ND | ND | ND | 1160 | 30.2 | ND | 99.1 | 95.4 | ND | ND | ND | ND | ND | ND | ND | ND |
| Antu and Nugraha, 2019 | Asia/ Indonesia | 2015 | Alb (400 mg) | Sch.Children | RCT | 807 | 185 | 6-14 | 9.2 | 35 | 25 | Kato-Katz | 1 | 13 | 13 | 0 | 0 | ND | 20.4 | 20.4 | 66.7 | 17 | 50 | 4 | 0 | ND | 0 | 100 | 100 | ND | ND | ND | ND | ND | ND | ND | ND |
|  |  |  | Alb (400 mg) + Levamisole (25 mg) |  |  |  |  |  | 8.9 | 28 | 32 |  |  | 26 | 24 | 2 | 0 | ND | 7.6 | 7.6 | 94.7 | 34 | 39 | 2 | 0 | ND | 0 | 100 | 100 | ND | ND | ND | ND | ND | ND | ND | ND |
|  |  |  | Meb (500 mg) plus Levamisole (25 mg) |  |  |  |  |  | 9.1 | 31 | 29 |  |  | 25 | 24 | 1 | 0 | ND | 8.8 | 8.8 | 92.3 | 11 | 46 | 1 | 0 | ND | 0 | 100 | 100 | ND | ND | ND | ND | ND | ND | ND | ND |
|  |  |  | Alb (400 mg) + Levamisole (25 mg) |  |  |  |  |  | 8.9 | 28 | 32 |  |  | 26 | 24 | 2 | 0 | ND | 7.6 | 7.6 | 94.7 | 34 | 39 | 2 | 0 | ND | 0 | 100 | 100 | ND | ND | ND | ND | ND | ND | ND | ND |
| Barda et al. 2018 | Afr/Tanzania |  | Alb (400 mg) + moxidectin (8mg) | Sch.Children | Singles blinded non-inferiority RCT | 942 | 701 | 12-18 |  |  |  | Kato-Katz | 2-3 | 634 | ND | ND | ND | ND | ND | ND | ND | ND | ND | ND | ND | ND | ND | ND | ND | ND | ND | ND | ND | ND | ND | ND | ND |
|  |  |  | Alb (400 mg) + Oxantel Pamoate (25mg/kg) |  |  |  |  |  |  |  |  |  |  |  |  |  |  |  |  |  |  |  |  |  |  |  |  |  |  |  |  |  |  |  |  |  |  |
| Bartoloni et al., 1993 | S.America/Venzueal | 1990 | Alb (400 mg) | Sch.Children | ND | 117 | 48 | 2-9 |  |  |  | Kato-Katz | 2-3 | 15 | ND | ND | ND | 148 |  |  |  | 6 | ND | ND | ND | 1900 |  |  |  | 33 | ND | ND | ND | 710 |  |  |  |
|  |  |  | Meb (500mg) |  |  |  |  |  |  |  |  |  |  | 11 | ND | ND | ND | 348 |  |  |  | 11 | ND | ND | ND | 5662 |  |  |  | 29 | ND | ND | ND | 1292 |  |  |  |
|  |  |  |  |  |  | 62 | 40 |  |  |  |  |  |  |  |  |  |  |  |  |  |  |  |  |  |  |  |  |  |  |  |  |  |  |  |  |  |  |
| Belizario et al., 2003 | Asia/Philipines | 1998 | Alb (400mg/kg)plus Ivermectin (200µg) | Sch.Children | RCT, placeb | 2284 | 784 | 6-12 |  | 1199 | 1085 | Kato-Katz | 1-2 | 149 | 61 | 68 | 20 | 4948.1 | 122.5 | 35.1 | 97.5 | 105 | 97.5 | 42 | 30 | 41011.4 | 198.8 | 97.5 | 99.5 | ND | ND | ND | ND | ND | ND | ND | ND |
|  |  |  | Alb (400mg/kg) |  |  |  |  |  |  |  |  |  |  | 149 | 58 | 75 | 17 | 6376.2 | 2930.8 | 31.5 | 54 | 99 | 54 | 50 | 14 | 21656 | 1520.2 | 93 | 93 | ND | ND | ND | ND | ND | ND | ND | ND |
|  |  |  | Alb (400mg/kg)plus Diethylcarbamazine (6mg) |  |  |  |  |  |  |  |  |  |  | 156 | 50 | 75 | 31 | 7544.2 | 1557.6 | 20 | 79.4 | 120 | 79 | 53 | 26 | 32821 | 1113.3 | 96.6 | 96.6 | ND | ND | ND | ND | ND | ND | ND | ND |
| Dalimunthe et al., 2007 | Asia/Indonesia | 2002 | Meb (500mg/kg)1X | Sch.Children | DBRCT | 326 | 311 |  | 10.5 |  |  | Kato-Katz | 4 | 70 | ND | ND | ND | ND | ND | 98.1 | 97.3 | 70 | ND | ND | ND | ND | ND | 99.9 | 96.9 | 70 | ND | ND | ND | ND | ND | 100 | 100 |
|  |  |  | Meb (100mg/kg)1X Pyrantel pamoate(10mg/kg), |  |  |  |  |  | 10.6 |  |  |  |  | 65 | ND | ND | ND | ND | ND | 97.9 | 94.2 | 65 | ND | ND | ND | ND | ND | 100 | 100 | 65 | ND | ND | ND | ND | ND | 100 | 100 |
| Ekenjoku et al., 2013 | Africa/Nigeria | 2005 | Meb (500mg/kg)1x | Sch.Children | RCT | 400 | 284 | 4-12 | ND | ND | ND | Conc.floatation | 1 | 19 | ND | ND | ND | ND | ND | 100 | 100 | 74 | ND | ND | ND | ND | ND | 100 | 100 | 101 | ND | ND | ND | ND | ND | ND | 90.45 |
| Eshetu et al., 2020 | Africa/ Ethiopia | 2019 | Meb (500 mg) | Sch.Children | RCT | 300 | 120 | 6-14 | 10.8 1 | 17 | 35 | McMaster | 2-3 | ND | ND | ND | ND | ND | ND | ND | ND | ND | ND | ND | ND | ND | ND | ND | ND | 52 | 43 | 7 | 2 | 1216 | 378 | 68.9 | 30.8 |
|  |  |  | Meb (multiple dose |  |  |  |  |  | 10.5 | 28 | 23 |  |  | ND | ND | ND | ND | ND | ND | ND | ND | ND | ND | ND | ND | ND | ND | ND | ND | 51 | 45 | 5 | 1 | 1134.3 | 5.88 | 99.5 | 96.1 |
| Ejigu et al., 2021 | Afr/Ethiopia/Bahirdar | 2018 | Meb (500mg/kg)1X | Sch.Children | Longitudinal | 422 | 296 | 7-18 | 11.87 | 203 | 219 | Kato-Katz | 2-4 | ND | ND | ND | ND | ND | ND | ND | ND | 89 |  |  |  | 129 | 79 | 76.4 | 60 | 148 |  |  |  | 281 | 215 | 53.1 | 32.4 |
| Flohr et al., 2007 | Asia/ Vietnam | 2005 | Meb (500mg) | Sch.Children | . RCT | 271 | 168 | 6-11 | 9 | 65 | 72 | Conc.floatation, McMaster | 2 | ND | ND | ND | ND | ND | ND | ND | ND | ND | ND | ND | ND | ND | ND | ND | ND | 90 | ND | ND | ND | 263 | 126 | 52 | 38 |
| Gebreyesus et al., 2024 | Afr/Ethiopia/Southern | 2019 | Alb (400mg/kg) (1x) | Sch.Children | Prospective cohort study | 5-15 |  |  |  |  |  | Kato-Katz | 4 | 109 | 109 | 0 | 0 | ND | ND | 814 |  |  | 550 | 240 | 24 | ND | ND |  |  | 455 | 441 | 14 | 0 | ND | ND |  |  |
| Getachew, 2014 | Afr/Ethiopia/Jimma | 2013/2014 | Alb (400mg/ Bendex | Sch.Children | RCT | 679 | 418 | 5-18 | 10.3 |  |  | McMaster, | 2 | 129 | ND | ND | ND | 909 | 688 | 24.4 |  | 106 | ND | ND | ND | 8706 | 1l12 |  | 98.7 | 56 | ND | ND | ND | 355 | 40 | 88.7 | ND |
|  |  |  | Alb (400mg/kg) Ovis® |  |  |  |  |  |  |  |  |  |  | 137 | ND | ND | ND | 769 | 612 | 20.4 |  | 101 | ND | ND | ND | 7935 | 175 |  | 97.8 | 56 | ND | ND | ND | 335 | 6 | 98.1 | ND |
| Humpharis et al., 2017 | Afr/Ghana | 2011 | Alb (400mg/kg) (1x) | Community | Cross-sectional | 140 | 82 | 6-13 |  |  |  | Kato-Katz | 2 | ND | ND | ND | ND | ND | ND | ND | ND | ND | ND | ND | ND | ND | ND | ND | ND | 82 | 79 | 3 | 0 |  |  |  |  |
| Husin et al., 2022 | Asia/ Indonesia/ | 2021 | Alb (400mg) | Sch.Children | RCT | 449 | 199 | 6-12 | 9 | 56 | 44 | Kato-Katz | 4 | 85 | 69 | 16 | 0 |  |  |  | 61.2 | 64 | 54 | 10 | 0 |  |  |  | 87.5 | 4 | 4 | 0 | 0 |  |  | 100 | 100 |
|  |  |  | Meb(500mg/kg) |  |  |  |  |  | 9 | 47 | 50 |  |  | 73 | 64 | 29 | 0 |  |  |  | 65.6 | 29 | 20 | 9 | 0 |  |  |  | 31 | 6 | 6 | 0 | 0 |  |  |  | 83.3 |
| Iqbal et al., 2021 | Asia/ Pakistan | 2018 | Alb (400-450mg) | Sch.Children | Cross-sectional | 296 | 192 | 6-15 | ND | ND | ND | Direct wet method | ND | ND | ND | ND | ND | ND | ND | ND | ND | ND | ND | ND | ND | ND | ND | ND | ND | 156 | 137 | 17 | 2 | 3405 | 852 | 75 | 75 |
|  |  |  | Meb (300 -350mg) |  |  |  |  |  |  |  |  |  |  | ND | ND | ND | ND | ND | ND | ND | ND | ND | ND | ND | ND | ND | ND | ND | ND |  |  |  |  | 2994 | 869 | 71 | 71 |
| Ismail et al., 1999 | Asia/ Sri lanka | 1999 | Alb (400mg) | Sch.Children | RCT | 176 | 155 | 4-14 | ND | 84 | 71 | Kato-Katz | 7 | 55 | ND | ND | ND | 1567 | 466 | 70.3 | 43.6 | ND | ND | ND | ND | ND | ND | ND | ND | ND | ND | ND | ND | ND | ND | ND | ND |
|  |  |  | Alb (400mg) + Diethylcarbamazine (6mg/kg) |  |  |  |  |  |  |  |  |  |  | 47 | ND | ND | ND | 1652.6 | 196 | 69 | 29.8 | ND | ND | ND | ND | ND | ND | ND | ND | ND | ND | ND | ND | ND | ND | ND | ND |
|  |  |  | Alb (400mg) + iver(200 µg/kg) |  |  |  |  |  |  |  |  |  |  | 33 | ND | ND | ND | 1544 | 78.7 | 93.8 | 79.3 | ND | ND | ND | ND | ND | ND | ND | ND | ND | ND | ND | ND | ND | ND | ND | ND |
| Kabatende et al., 2023 | Afri/Rwanda/ | 2019 | Alb (400mg/kg) | Sch.Children | Cross-sectional | 4998 | 1526 | 5-15 |  |  |  | Kato-Katz |  | 1404 | 1262 | 137 | 5 |  |  | 17.6 | 40.3 | 1064 | 791 | 254 | 19 |  |  | 94.6 | 95.1 | 61 | 61 | 0 | 0 |  |  | 97.4 | 96.7 |
| Keller et al., 2016 | Africa | 2018 | Alb (400mg) plus Moxidectin (8mg) | Sch.Children | RCT | 379 | 294 | 16-18 | 16.8 | 30 | 12 | Kato-Katz | 2-3 | 42 | 38 | 4 | 0 | 133.2 | 3.4 | 97.4 | 62.5 | 7 | 6 | 1 | 0 | 3141.3 | 0 | 100 | 75 | 11 | 11 | 0 | 0 | 82.5 | 0.3 | 99.7 | 81.8 |
|  |  |  | Alb (400mg) plusMoxidectin (16mg) |  |  |  |  |  | 16.8 | 26 | 16 |  |  | 42 | 38 | 4 | 0 | 174.8 | 2.9 | 98.4 | 61.9 | 4 | 1 | 3 | 0 | 3190.6 | 0.9 | 100 | 100 | 15 | 14 | 1 | 0 | 88.3 | 1.5 | 98.3 | 80 |
|  |  |  | Alb (400mg)plus Moxidectin (24mg) |  |  |  |  |  | 17 .0 | 28 | 13 |  |  | 41 | 37 | 4 | 0 | 165.4 | 2.3 | 98.6 | 69.2 | 7 | 4 | 3 | 0 | 9261.4 | 989.1 | 89.3 | 20 | 10 | 10 | 0 | 0 | 49.9 | 0.2 | 99.6 | 90 |
| Knopp et al., 2010 | Africa/ Tanzania, Zanzibar, | 2009 | Alb plus placebo | Sch.Children | RCT | 1240 | 610 | >5 | 10.9 | 79 | 71 | Kato-katz | 3 | 150 | 138 | 12 | 0 | 154 | 154 | 40.3 | 9.8 | 19 | 11 | 7 | 1 | 2680 | 0 | 100 | 100 | 44 | 44 | 0 | 0 | 67 | 4 | 94 | 59 |
|  |  |  | Alb plus iver |  |  |  |  |  | 11 | 69 | 84 |  |  | 153 | 142 | 11 | 0 | 127 | 121 | 91.1 | 37.9 | 15 | 10 | 4 | 1 | 1699 | 1 | 99.9 | 92.86 | 37 | 36 | 1 | 0 | 61 | 13 | 78.7 | 66.67 |
|  |  |  | Mebplus placebo |  |  |  |  |  | 10.8 | 64 | 89 |  |  | 153 | 144 | 9 | 0 | 146 | 136 | 66.7 | 18.6 | 19 | 8 | 11 | 0 | 2414 | 5 | 99.79 | 77.78 | 39 | 39 | 0 | 0 | 6774 | 43 | 99.76 | 35.3 |
|  |  |  | Meb plus iver |  |  |  |  |  | 11 | 62 | 92 |  |  | 154 | 144 | 10 | 0 | 160 | 154 | 96.7 | 55.1 | 20 | 14 | 5 | 1 | 553 | 0 | 100 | 100 | 40 | 40 | 0 | 0 | 61 | 13 | 78.68 | 25.7 |
| Kihara et al., 2007 | Africa/Kenya | 2004 | Alb (400mg/kg) plus praziquantol | Sch.Children | Cross-sectional | 2300 | 441 | 4-18 |  |  |  | Kato-Katz | 8 | 20 | 20 | 0 | 0 | ND | ND | 45 | 18.2 | 42 | 42 | 0 | 0 | ND | ND | 100 | 100 | 396 | 393 | 2 | 1 | ND | ND | 96 | 96 |
| Krücken et al., 2017 | Africa/Rwanda | 2014 | Alb (400mg/kg)1x | Sch.Children | Cross-sectional | 1182 | 698 | 6-10 | 8 | 477 | 705 | Conc, flot  PCR | 1-2 | ND | ND | ND | ND | ND | ND | ND | ND | ND | 394 | ND | ND | ND | ND | 75.4 | 86.7 | ND | ND | ND | ND | ND | ND | ND | ND |
| Levecke et al., 2014 | SA/Brazil | 2011-2012 | Meb (500mg/kg) | Sch.Children | Cross-sectional | 5830 | 2589 | 4-18 | 9.3 |  |  | McMaster, kato-katz. Conc. floatation | 2 | 0 | 0 | 0 | 0 | 0 |  | 0 |  | 133 | 74 | 58 | 1 | 8076 |  | 97.5 |  | 172 | 167 | 5 | 0 | 420 |  | 84.4 | ND |
|  | Afr/Cameroon |  |  |  |  |  |  |  | 10 |  |  |  |  | 175 | 160 | 19 | 0 | 420 |  | 56.2 |  | 132 | 83 | 48 | 1 | 6006 |  | 99.8 |  | 161 | 157 | 2 | 1 | 406 |  | 71.9 | ND |
|  | SA?Cambodia |  |  |  |  |  |  |  |  |  |  |  |  | ND | ND | ND | ND | ND | ND | ND | ND | ND | ND | ND | ND | ND | ND | ND | ND | 160 | 158 | 3 | 0 | 310 |  | 79.1 | ND |
|  | Afr/Ethiopia |  |  |  |  |  |  |  | 11.5 |  |  |  |  | 326 | 273 | 52 | 10 | 653 |  | 65.9 |  | 279 | 172 | 105 | 2 | 6497 |  | 98.6 |  | 100 | 100 | 0 | 0 | 266 |  | 65.4 | ND |
|  | Afr/Tanzania |  |  |  |  |  |  |  | 10.7 |  |  |  |  | 505 | 238 | 256 | 11 | 2092 |  | 51.2 |  | 378 | 234 | 140 | 30 | 6761 |  | 97.1 |  | 226 | 202 | 17 | 7 | 854 |  | 74.6 | ND |
|  | Asia/Veitnam |  |  |  |  |  |  |  | 8.4 |  |  |  |  | 69 | 57 | 12 | 0 | 546 |  | 76.8 |  | 289 | 115 | 140 | 32 | 30857 |  | 93.1 |  | 80 | 75 | 4 | 1 | 679 |  | 95 | ND |
| Legesse et al .,2004 | Africa/ Ethiopia | 2003 | Alb(400 mg) | Sch.Children | RCT | 717 | 703 | 6-19 | 10.5 | 55 | 75 | Kato-Katz | 3 | 130 | ND | ND | ND | 445.7 | 258 | 69.8 | 17.1 | 130 | ND | ND | ND | 6982.2 | 305.1 | 99.9 | 92.5 | ND | ND | ND | ND | ND | ND | ND | ND |
|  |  |  | Meb (100mg) East Afr |  |  |  |  |  | 10.1 | 53 | 90 |  |  | 143 | ND | ND | ND | 522.3 | 172 | 88.5 | 27.9 | 143 | ND | ND | ND | 6198.2 | 1295.9 | 99.9 | 93 | ND | ND | ND | ND | ND | ND | ND | ND |
|  |  |  | Meb (100mg) Indian |  |  |  |  |  | 10.9 | 47 | 76 |  |  | 123 | ND | ND | ND | 376.4 | 118 | 96.5 | 53.5 | 123 | ND | ND | ND | 6925.5 | 384 | 99.9 | 99 | ND | ND | ND | ND | ND | ND | ND | ND |
|  |  |  | Meb (100mg) SouthAfri |  |  |  |  |  | 11.1 | 54 | 84 |  |  | 138 | ND | ND | ND | 414.1 | 91.4 | 99.1 | 89.8 | 138 | ND | ND | ND | 8188.9 | 1833.2 | 99.9 | 96.5 | ND | ND | ND | ND | ND | ND | ND | ND |
| Lubis et al., 2012 | Asia/Indonesia |  | Alb (400mg/kg)1x | Sch.Children | Cross-sectional | ND | 229 |  |  |  |  | Kato-Katz | 3-4 | ND | ND | ND | ND | ND | ND | ND | ND | 123 | 105 | 17 | 1 |  |  | 99.3 | 96.7 | ND | ND | ND | ND | ND | ND | ND | ND |
|  |  |  | Meb (500mg/kg)1X |  |  |  |  |  |  |  |  |  |  | ND | ND | ND | ND | ND | ND | ND | ND | 106 | 84 | 21 | 1 |  |  | 100 | 100 | ND | ND | ND | ND | ND | ND | ND | ND |
| Mani et al., 2002 | Asia/ India | 1998-2000 | Alb plus Diethylcarbamazine | Sch.Children | Cross-sectional | 646 | 321 | 1–15 | ND | 177 | 144 | Kato-Katz | 3 | 15 | 15 | 0 | 0 | 81.58 | 0.27 | 83.96 | 81.58 | 176 | 176 | 0 | 0 | 23.51 | 0.81 | 96.6 | 74.3 | 53 | 53 | 0 | 0 | 1.48 | 0.09 | 94.2 | 89.5 |
| Matamoros et al ., 2021 | Central America/ Honduras | 2019 | Alb 1X (400 mg) | Sch.Children | RCT | 377 | 176 | 2–14 | 8.8 | 12 | 12 | Kato-Katz | 2-3 | 24 | ND | ND | ND | 1402.4 | 733 | 47.7 | 4.2 | ND | ND | ND | ND | ND | ND | ND | ND | ND | ND | ND | ND | ND | ND | ND | ND |
|  |  |  | Alb 1X(400 mg) plus iver (600 μg/kg) |  |  |  |  |  | 8.1 | 20 | 15 |  |  | 35 | ND | ND | ND | 1593.5 | 54.2 | 96.7 | 88.6 | ND | ND | ND | ND | ND | ND | ND | ND | ND | ND | ND | ND | ND | ND | ND | ND |
|  |  |  | Alb (400 mg) for 3 days |  |  |  |  |  | 8.9 | 8 | 10 |  |  | 18 | ND | ND | ND | 1233.1 | 344 | 72.1 | 33.3 | ND | ND | ND | ND | ND | ND | ND | ND | ND | ND | ND | ND | ND | ND | ND | ND |
|  |  |  | Alb (400 mg) plus iver (600 μg/kg) for 3 days |  |  |  |  |  | 8.2 | 20 | 20 |  |  | 40 | ND | ND | ND | 1159.7 | 0 | 100 | 100 | ND | ND | ND | ND | ND | ND | ND | ND | ND | ND | ND | ND | ND | ND | ND | ND |
| Moser et al., 2018 | Asia/ Laos | 2017 | Alb (400mg) pyrantel pamoate(20mg/kg), and oxantel pamoate(20mg/kg) | Sch.Children | RCT | 1524 | 533 | 6-15 | 12.3 | 71 | 70 | Kato-Katz | 4 | 43 | 42 | 1 | 0 | 68.1 | 0.3 | 99.6 | 93 | 26 | 15 | 5 | 2 | 3363.8 | 0.5 | 99.9 | 90.9 | 141 | 112 | 15 | 14 | 671.4 | 0.9 | 99.9 | 84.1 |
|  |  |  | Alb (400mg) plus oxantel pamoate(20mg/kg) |  |  |  |  |  | 12.4 | 74 | 66 |  |  | 52 | 4 | 0 | 138 | 88.8 | 0 | 100 | 100 | 24 | 16 | 6 | 2 | 2710.3 | 0.2 | 99.9 | 95.8 | 140 | 112 | 20 | 8 | 706.9 | 7.2 | 99 | 52.9 |
|  |  |  | Meb (500mg) Pyrantel pamoate(20mg/kg), and oxantel pamoate(20mg/kg) |  |  |  |  |  | 12.2 | 43 | 26 |  |  | 26 | 1 | 0 | 69 | 49.1 | 0.6 | 98.8 | 88.5 | 15 | 11 | 3 | 1 | 2720.3 | 0 | 100 | 100 | 69 | 56 | 9 | 4 | 703.8 | 2.5 | 99.6 | 69.6 |
| Moser et al., 2018 |  |  | Alb (400mg) plus oxantel pamoate(20mg/kg) |  |  |  |  |  |  |  |  |  |  |  |  |  |  |  |  |  |  |  |  |  |  |  |  |  |  |  |  |  |  |  |  |  |  |
| Müller et al., 2016 | Afri/South Africa | 2015 | Alb (400mg/kg)1x | Sch.Children | Cross-sectional | ND | ND | 9-14 | 73 | 74 |  | Kato-Katz | 2 | 90 | 59 | 28 | 3 | 1082 | 587 | 46 | 1.1 | 71 | 24 | 38 | 9 | 10952 | 629 | 94.3 | 97.2 | ND | ND | ND | ND | ND | ND | ND | ND |
| Muchiri et al., 2001 | Africa/Kenya | 1996 | Meb (600 mg) | Sch.Children | RCT | 1186 | 726 | 4 -19 | 10.9 | 195 | 221 | Kato-Katz | 4 | 293 | ND | ND | ND | 61 | 4 | 93.4 | 60.6 | 416 | ND | ND | ND | 2227 | 12 | 99.4 | 79.6 | 256 | ND | ND | ND | 196 | 66 | 66.3 | 50 |
|  |  |  | Meb (600) (every 4months) |  |  |  |  |  | 10.1 | 201 | 196 |  |  | 226 |  |  |  | 101 | 6 | 94.1 | 68.3 | 397 |  |  |  | 2062 | 2 | 99.9 | 97.5 | 258 |  |  |  | 215 | 32 | 85.1 | 55 |
|  |  |  | Alb (600mg/kg |  |  |  |  |  | 11.2 | 164, | 209 |  |  | 268 |  |  |  | 74 | 7 | 90.5 | 67.8 | 373 |  |  |  | 1924 | 7 | 99.6 | 83.5 | 212 |  |  |  | 120 | 4 | 96.7 | 92.4 |
| Nadyne et al., 2017 | Afric/Cameroon | 2015 | Meb (500mg/kg)3days | Sch.Children | Cross-sectional | 410 | 259 | 3-15 |  | 201 | 209 | Kato-Katz | 10 | 239 | 184 | 41 | 14 | 39.9 | 60.7 | 39.9 | 60.7 | 105 | 71 | 35 | 9 | 12854.4 | 706.3 | 94.5 | 93.9 | 24 | 22 | 2 | 0 | 378 | 178 | 52.9 | 70.8 |
| Nasution et al., 2014 | Asia/Indonesia | 2009 | Alb (400mg/kg) | Sch.Children | RCT | 212 | 116 | >2 | 9.5 |  |  | Kato-Katz | 4 | 53 | ND | ND | ND | 208.3 | 37.4 | 54.5 | 66 | ND | ND | ND | ND | ND | ND | ND | ND | ND | ND | ND | ND | ND | ND | ND | ND |
|  |  |  | Alb (400mg/kg)plus Diethylcarbamazine (6mg) |  |  |  |  |  | 9.6 |  |  |  |  | 55 | ND | ND | ND | 204.1 | 51.8 | 60.7 | 60 | ND | ND | ND | ND | ND | ND | ND | ND | ND | ND | ND | ND | ND | ND | ND | ND |
| Nkengazong et al., 2010 | Afric/Cameroon | 2007 | Alb (600mg/kg) 3days | Sch.Children | Cross-sectional | 420 | 178 | 1-20 |  |  |  | Kato-Katz | 12 | 150 | ND | ND | ND | ND | ND | 55.3 | 84.6 | 131 | ND | ND | ND | ND | ND | 52.2 | 82 | 10 | ND | ND | ND | ND | ND | 100 | 100 |
| Ngonjo et al., 2015 | Africa/Kenya | 2014 | Alb (400mg) | Sch.Children | Cross-sectional | 1396 | 731 | 4-16 |  |  |  | Kato-Katz | 2 | 11 | ND | ND | ND | ND | ND | 100 | 100 | 607 | ND | ND | ND | 3673 | 59 | 94.8 | 98.4 | 5 | ND | ND | ND | ND | ND | ND | ND |
| Nisha et al., 2021 | Asia/ Malaysia | 2019 | Alb (400mg/kg) | Community | Cross-sectional | 68 | 58 | 2-17 | ND | 33 | 35 | McMaster | 4 | 58 | ND | ND | ND | 1814.7 | 805 | 55.7 | 41.4 | 58 | ND | ND | ND | 24907 | 8.9 | 99.9 | 93.1 | ND | ND | ND | ND | ND | ND | ND | ND |
| Nontasut et al., 1997 | Asia/ Thailand | 1997 | Alb (400mg) | Sch.Children | Cross-sectional | ND | ND | ND | ND | ND | ND | kato-katz | 4 | 27 | ND | ND | ND | ND | ND | ND | 33.3 | 17 | ND | ND | ND | ND | ND | ND | 83.3 | 24 | ND | ND | ND | ND | ND | ND | 91.6 |
|  |  |  | meb (100mg) |  |  |  |  |  |  |  |  |  |  | 33 | ND | ND | ND | ND | ND | ND | 93.3 | 33 | ND | ND | ND | ND | ND | ND | 100 | 33 | ND | ND | ND | ND | ND | ND | 81.8 |
|  |  |  | Mab (25mg) |  |  |  |  |  |  |  |  |  |  | 21 | ND | ND | ND | ND | ND | ND | 38.1 | 15 | ND | ND | ND | ND | ND | ND | 93.3 | 25 | ND | ND | ND | ND | ND | ND | 64 |
|  |  |  | Meb (50mg0 |  |  |  |  |  |  |  |  |  |  | 29 | ND | ND | ND | ND | ND | ND | 41.4 | 21 | ND | ND | ND | ND | ND | ND | 90.5 | 37 | ND | ND | ND | ND | ND | ND | 48.6 |
|  |  |  | Meb (75mg) |  |  |  |  |  |  |  |  |  |  | 31 | ND | ND | ND | ND | ND | ND | 51.4 | 17 | ND | ND | ND | ND | ND | ND | 88.2 | 17 | ND | ND | ND | ND | ND | ND | 35.3 |
| Norhayati et al., 1997 | Asia/ Malaysia | 1997 | Alb (400mg) | Sch.Children | Cross-sectional | 205 | 123 | 1-13 | ND | 95 | 110 | kato-katz | 4 | 110 | ND | ND | ND | 13904 | 7084 | 49.1 | 5.5 | 77 | ND | ND | ND | 39814 | 32 | 99.9 | 97.4 | 29 | ND | ND | ND | 464 | 66 | 96.6 | 93.1 |
| Ortiz et al., 2002 | South America/ Peru | 2000 | Alb (400mg) | ND | RCT | ND | ND | 2-l 1 | 7.9 | 100 | 110 | Kato-Katz | 3 | 19 | 19 | 0 | 0 | 335 | 5 | 98.4 | 58 | 35 | 24 | 8 | 3 | 1291 | 1 | 99.9 | 91 | ND | ND | ND | ND | ND | ND | ND | ND |
|  |  |  | Alb (400mg) |  |  |  |  |  | 3.8 | 16 | 14 |  |  | 23 | 24 | 5 | 1 | 327.4 | 42.4 | 87.1 | 17.4 | 5 | 3 | 2 | 0 | 3958.5 | 14.3 | 99.6 | 66.7 | 1 | ND | ND | ND | ND | ND | 100 | 100 |
|  |  |  | Alb (600 mg) |  |  |  |  |  | 4.0 | 18 | 8 |  |  | 18 | 21 | 6 | 0 | 324.7 | 37.4 | 88.5 | 27.8 | 3 | 2 | 1 | 0 | 30 | 0 | 100 | 100 | 0 | 0 | 0 | 0 | 0 | 0 | 0 | ND |
|  |  |  | Alb (400mg) |  |  |  |  |  | 8.9 | 15 | 30 |  |  | 43 | 30 | 15 | 0 | 542.3 | 97.4 | 82 | 16.3 | 18 | 8 | 9 | 1 | 2781 | 0.9 | 99.9 | 88.9 | 1 | ND | ND | ND | ND | ND | 0 | 0 |
|  |  |  | Alb (600 mg) |  |  |  |  |  | 8.7 | 19 | 27 |  |  | 43 | 31 | 15 | 0 | 517.3 | 59.9 | 88.4 | 25.6 | 10 | 4 | 6 | 0 | 3770.7 | 0 | 100 | 100 | 2 | ND | ND | ND | ND | ND | 100 | 100 |
|  |  |  | Alb (800mg) |  |  |  |  |  | 8.8 | 17 | 33 |  |  | 41 | 30 | 12 | 1 | 507.9 | 108 | 78.8 | 17.1 | 13 | 6 | 7 | 0 | 3262 | 0.9 | 99.9 | 84.6 | 3 | ND | ND | ND | ND | ND | 100 | 100 |
| Palmeirim et al., 2018 | Africa/ Tanzania | 2017 | Meb 500 mg | Sch.Children | RCT | 354 | 206 | 6-12 | 10.1 | 54 | 39 | Kato-Katz | 3 | 92 | 59 | 30 | 0 | 655.9 | 186 | 71.7 | 6.8 | 93 | 28 | 15 | 0 | 2698.5 | 0 | 100 | 100 | 93 | 89 | 3 | 1 | 220.2 | 70.5 | 52.7 | 13 |
|  |  |  | Meb 100 mg/kg (multiple dose |  |  |  |  |  | 10.1 | 47 | 46 |  |  | 93 | 56 | 35 | 0 | 726.8 | 14 | 98.1 | 42.9 | 93 | 20 | 28 | 0 | 4113 | 0.2 | 100 | 98 | 93 | 90 | 3 | 0 | 234.9 | 0.1 | 99.8 | 97.9 |
| Palmeirim et al., 2020 | Africa/ Tanzania | 2019 | Meb (chewable) | Sch.Children | RCT | 1465 | 500 | 3-12 | 9.4 | 110 | 88 | Kato-Katz | 2-3 | 193 | 118 | 75 | 2 | 631.5 | 169 | 73.3 | 9.8 | 105 | 36 | 57 | 14 | 6811.6 | 0.3 | 98.7 | 95.3 | 197 | 191 | 5 | 1 | 219 | 69 | 38.1 | 12.7 |
|  |  |  | Meb (solid) |  |  |  |  |  | 9.3 | 118 | 80 |  |  | 193 | 126 | 68 | 1 | 576.7 | 149 | 74.2 | 7.3 | 89 | 38 | 43 | 10 | 7352.4 | 0.2 | 99.8 | 97.8 | 196 | 192 | 4 | 2 | 226.7 | 66.2 | 28.1 | 11.2 |
| Patel et al., 2020 | Africa/ Cote d'Ivoire | 2018-2019 | Alb (200mg) | Sch.Children | RCT | 493 | 187 | 2-12 | 4.0 | 18 | 9 | Kato-Katz | 3 | 21 | 22 | 5 | 0 | 273.3 | 99.1 | 63.8 | 9.5 | 8 | 2 | 6 | 0 | 3436 | 0.9 | 99.9 | 83.3 | 0 | 0 | 0 | 0 | ND | ND | ND | ND |
| Payne et al., 2016 | Africa/ Cameroon | 2016 | Meb (500 mg) | Sch.Children | Cross-sectional | 948 | 50 | 3-21 | 8.61 | 477 | 471 | Conc McMaster | 3 | 7 | 7 | 0 | 0 | 50 | 43 | 14 | 14.29 | 16 | 14 | 1 | 1 | 1895 | 0 | 100 | 100 | 27 | 27 | 0 | 0 | 65 | 15 | 77 | 70.4 |
| Putra et al., 2005 | Asia/Indonesia | 2002 | Alb (400mg/kg)1x | Sch.Children | RCT | 434 | 333 | 6-13 | 8.6 |  |  | Kato-Katz | 4 | ND | ND | ND | ND | ND | ND | ND | ND | 134 | 57 | 57 | 20 | ND | ND | 100 | 100 | ND | ND | ND | ND | ND | ND | ND | ND |
| Rahman et al., 1996 | Asia/ Malaysia | 1990 | Alb (400mg) | Sch.Children | Cross-sectional | 192 | ND | 0-12 | ND | ND | ND | conc. | 4 | 184 | ND | ND | ND | ND | ND | ND | 83.4 | 67 | ND | ND | ND | ND | ND | ND | 87.3 | 37 | ND | ND | ND | ND | ND | ND | 89.1 |
|  |  |  | Meb (400mg) |  |  |  |  |  |  |  |  |  |  |  | ND | ND | ND | ND | ND | ND | 33.3 | 18 | ND | ND | ND | ND | ND | ND | 83.3 |  | ND | ND | ND | ND | ND | ND |  |
| Rochmah et al., 2016 | Asia/ Indonesia | 2013 | Alb 9400mg) | Sch.Children | Cross-sectional | 65 | 28 | 7-13 | ND | 35 | 30 | Kato-Katz | 2 | 18 | ND | ND | ND | 582 | 219 | 62.4 | 12.8 | ND | ND | ND | ND | ND | ND | ND | ND | ND | ND | ND | ND | ND | ND | ND | ND |
| Sam, 2011 | Asia/ Vellore | 2008 | Alb | Sch.Children | Cross-sectional | 2549 | 161 | 6-14 | 11.2 | 1300 | 1249 | Kato-Katz, McMaster | 2 | 22 | 20 | 2 | 0 | ND | ND | 76 | 84.6 | 33 | 27 | 9 | 0 | ND | ND | 100 | 100 | 24 | 23 | 1 | 0 | ND | ND | 96 | 83.6 |
| Samuel et al., 2014 | Africa/ Ethiopia |  | Alb (400mg) | Sch.Children | Cross-sectional | 298 | 263 | 5-16 |  | 167 | 153 | Kato-Katz | 3 | 133 | 122 | 11 | 0 | 145.7 | 24.6 | 83.1 | 30.8 | 148 | 130 | 18 | 0 | 1224.9 | 1.2 | 99.9 | 96.6 | 142 | 112 | 19 | 11 | 1101 | 1.1 | 99.8 | 97.4 |
| Sam-Wobo et al., 2021 | Africa/Nigeria | 2016 | Alb (400mg/kg)1x | Sch.Children | Cross-sectional | 282 | 151 | 6-16 |  |  |  | Kato-Katz | 3 | 3 | 3 | 0 | 0 | 288 | 0 | 69.2 | ND | 131 | 107 | 24 | 0 | 5096 | 2 | 99.7 | ND | 60 | 48 | 12 | 0 | 2202 | 2 | 99.4 |  |
| Sapulete et al., 2020 | Asia/Indonesia | 2017-2018 | Alb (400mg) | Sch.Children | RCT | 600 | 392 | 8-12 | 9.3 |  |  | Kato-Katz | 8 | 30 | 30 | 0 | 0 | 10 | 0 | 55.3 | 60 | ND | ND | ND | ND | ND | ND | ND | ND | ND | ND | ND | ND | ND | ND | ND | ND |
|  |  |  | Alb (400mg) plus pyrantel pamoate 10 mg/kg |  |  |  |  |  | 9.4 |  |  |  |  | 30 | 30 | 0 | 0 | 14 | 3 | 44.7 | 40 | ND | ND | ND | ND | ND | ND | ND | ND | ND | ND | ND | ND | ND | ND | ND | ND |
| Silber et al.,2017 | Africa/ Ethiopia, Rwanda | 2015 | Meb (500mg/kg) | Health facilities | RCT | 726 | 295 | 1-16 | 7.9 | 71 | 78 | Kato-Katz | 3 | 124 | 100 | 24 | 0 | 647.8 | 261 | 59.7 | 42 | 86 | 34 | 45 | 7 | 17,610.10 | 366.7 | 97.9 | 83.7 | 4 | 4 | 0 | 0 | ND | ND | 100 | 100 |
| Soukhathammavong et al., 2012 | Asia/Lao PDR | 2009 | Alb(400 mg) | Sch.Children | RCT | 465 | 200 | 6–12 | 9.0 | 130 | 70 | Kato-Katz | 3 | 39 | 38 | 4 | 0 | na | 75 | 67 | 33.3 | 28 | 18 | 7 | 3 | 1,567 | 0 | 100 | 92.9 | 89 | 72 | 9 | 8 | 859.1 | 63 | 86.7 | 36 |
|  |  |  | Meb (500 mg) |  |  |  |  |  | 9.0 | 49 | 33 |  |  | 43 | 43 | 0 | 0 | na | 48 | 66 | 27.9 | 30 | 18 | 8 | 3 | 1,584 | 0 | 100 | 93.3 | 82 | 67 | 7 | 8 | 707 | 147 | 76.3 | 17.6 |
| Speich et al., 2012 | Afri/Tanzania/Pemba | 2011 | Alb (400mg/kg)1x | Sch.Children | RCT | 701 | 353 |  | 9.7 | 85 | 92 | Kato-Katz | 3 | 142 | 131 | 10 | 1 | 164 | 9 | 45.6 | 14.5 | 9 | 9 | 0 | 0 | 207 | 100 | 100 |  | 11 | ND | ND | ND | ND | ND | ND | 81.8 |
|  |  |  | Alb (400mg/kg)1x plus Nitazoxanide (1000mg) |  |  |  |  |  | 9.8 | 82 | 83 |  |  | 142 | 133 | 10 | 0 | 152 | 69 | 54.9 | 16 | 6 | 5 | 1 | 0 | 686 | 100 | 100 |  | 15 | ND | ND | ND | ND | ND | ND | 85.5 |
| Subba and Singh, 2020 | Asia/ India | 2016 | Alb (400mg/kg) | Sch.Children | Cross-sectional | 300 | 10 | 5-18 | ND | ND | ND | Wet mount, conc | 2 | 1 | ND | ND | ND | 100 | 0 | 100 | 100 | 9 | ND | ND | ND | 200-1500 | 100-600 | 81.4 | 55.5 | ND | ND | ND | ND | ND | ND | ND | ND |
| Speich et al., 2015 | Africa/ Tanzania | 2013 | Alb ( 400 mg) plus iver (200 µg/kg) | Sch.Children | RCT | 650 | 440 | 6- 14 | 9.0 | 56 | 54 | Kato-Katz | 3 | 110 | 75 | 35 | 0 | 489 | 27 | 94.5 | 30 | 50 | 27 | 20 | 3 | 2023 | 0.04 | 100 | 98 | 43 | 41 | 2 | 0 | 108 | 5 | 17.5 | 22.2 |
|  |  |  | Alb (400 mg) plus meb (500 mg) |  |  |  |  |  | 8.9 | 57 | 53 |  |  | 110 | 78 | 31 | 1 | 409 | 198 | 51.6 | 9 | 40 | 20 | 17 | 3 | 1074 | 0.29 | 99.9 | 97.5 | 48 | 46 | 1 | 1 | 137 | 10 | 95.4 | 250 |
|  |  |  | Alb (400 mg) plus Oxantel Pamoate (20 mg/kg) |  |  |  |  |  | 8.9 | 58 | 52 |  |  | 110 | 72 | 37 | 1 | 491 | 4 | 99.2 | 74 | 48 | 29 | 18 | 16 | 1378 | 0.22 | 99.9 | 97.9 | 55 | 54 | 1 | 0 | 88 | 8 | 92.7 | 47.8 |
|  |  |  | Meb (500 mg) |  |  |  |  |  | 8.8 | 55 | 55 |  |  | 110 | 82 | 28 | 0 | 479 | 199 | 58.5 | 9 | 47 | 30 | 16 | 1 | 1035 | 0.36 | 100 | 95.5 | 43 | 43 | 0 | 0 | 79 | 32 | 90.9 | 45.5 |
| Speich et al., 2016 | Africa/ Tanzania | 2013-2014 | Alb (400 mg) plus iver (200 μg/kg | Sch.Children | RCT | 650 | 405 | 6-14 | ND | ND | ND | Kato-Katz | 3 | 100 | ND | ND | ND | 489.9 | 25 | 94.9 | 28 | 46 | ND | ND | ND | 2385.8 | 0.1 | 99.9 | 97.8 | 100 | ND | ND | ND | 113.1 | 6.1 | 94.6 | 44.7 |
|  |  |  | Alb (400 mg) – meb (500 mg) |  |  |  |  |  |  |  |  |  |  | 101 | ND | ND | ND | 390 | 176 | 54.9 | 8.9 | 36 | ND | ND | ND | 1195.3 | 0 | 100 | 100 | 101 | ND | ND | ND | 139.8 | 8.3 | 94.1 | 48.8 |
|  |  |  | Alb (400 mg) – oxantel pamoate (20 mg/kg) |  |  |  |  |  |  |  |  |  |  | 100 | ND | ND | ND | 471.3 | 3.7 | 99.2 | 68 | 43 | ND | ND | ND | 1503.4 | 0.2 | 99.9 | 97.7 | 100 | ND | ND | ND | 89.2 | 7.1 | 91.9 | 48 |
|  |  |  | Meb (500 mg) |  |  |  |  |  |  |  |  |  |  | 104 | ND | ND | ND | 467.8 | 208 | 55.6 | 7.7 | 41 | ND | ND | ND | 1095.1 | 0.4 | 99.9 | 95.4 | 104 | ND | ND | ND | 80 | 31.8 | 60.3 | 24.4 |
| Sungkar et al., 2019 | Asia?/Indonesia | 2016 | Alb (400mg/kg) 3days | Sch.Children | Cross-sectional | 246 | 192 | 1-15 |  | 82 | 132 | kato-katz | 2 | 157 | ND | ND |  | 1344 | 12 | 91 | 61 | 148 | ND | ND | ND | 8592 | 24 | 100 | 97 | 24 | ND | ND | ND | 24 | 0 | 100 | 100 |
| Suteno et al., 2020 | Asia/ Indonesia/ Talawi, Batu Bara | 2020 | Alb (400mg/kg) | Sch.Children | RCT | 463 | 235 | 6-12 | 9.3 | 61 | 36 | Kato-Katz | 4 | 99 | 60 | 30 | 0 | 912.01 | 223.87 | 75.45 | 52.5 | ND | ND | ND | ND | ND | ND | ND | ND | ND | ND | ND | ND | ND | ND | ND | ND |
|  |  |  | Alb (400mg/kg) + Meb (500mg/kg) |  |  |  |  |  | 8.5 | 52 | 45 |  |  | 97 | 64 | 32 | 0 | 2041 | 131.82 | 93.5 | 71.1 | ND | ND | ND | ND | ND | ND | ND | ND | ND | ND | ND | ND | ND | ND | ND | ND |
| Tefera et al., 2015 | Afr/Ethiopia/Jimma | 2010 | Alb (400mg/kg)1x | Sch.Children | RCT | 715 | 326 |  |  |  |  | McMaster |  | 67 | 143 | 14 | 0 | 436.24 | 139.5 | 99.9 | 99.4 | 169 | 149 | 10 | 3 | 2052 | 4.94 | 99.9 | 59.9 | 165 | 62 | 1 | 0 | 395.24 | 26.98 | 99.9 | 93.7 |
| Vercruysse et al., 2011 | Africa,/ Ethiopia/ Tanzania /Cameroon Asia/ India Latine America/ Brazil | 2009 | Alb | Sch.Children | RCT | 8841 | 2319 | 4- 18 | ND | 1358 | 1552 | McMaster | 2-4 | 105 | 823 | 215 | 8 | ND | ND | 99.9 | 85.7 | 151 | 662 | 270 | 20 | ND | ND | 99.1 | 93.3 | 91 | 859 | 40 | 13 | ND | ND | 99.9 | 98.9 |
|  |  |  |  |  |  |  |  |  |  |  |  |  |  | 1 |  |  |  | ND | ND | 100 | 100 | 50 | ND | ND | ND | ND | ND | 98 | 98 | 52 | ND | ND | ND | ND | ND | 99.6 | 52 |
|  |  |  |  |  |  |  |  |  |  |  |  |  |  | 2 |  |  |  | ND | ND | 99.6 | 100 | 5 | ND | ND | ND | ND | ND | 99.2 | 100 | 127 | ND | ND | ND | ND | ND | 99.5 | 87.4 |
|  |  |  |  |  |  |  |  |  |  |  |  |  |  | 386 |  |  |  | ND | ND | 99.9 | 47.4 | 298 | ND | ND | ND | ND | ND | 93 | 99.3 | 140 | ND | ND | ND | ND | ND | 91.9 | 87.1 |
|  |  |  |  |  |  |  |  |  |  |  |  |  |  | 18 |  |  |  | ND | ND | 98.9 | 88.9 | 21 | ND | ND | ND | ND | ND | 98.9 | 95.2 | 95 | ND | ND | ND | ND | ND | 81.6 | 74.4 |
|  |  |  |  |  |  |  |  |  |  |  |  |  |  | 396 |  |  |  | ND | ND | 100 | 21 | 279 | ND | ND | ND | ND | ND | 82.6 | 96.4 | 349 | ND | ND | ND | ND | ND | 92.6 | 86.6 |
| Walker et al., 2021 | Afr/Ethiopia | 2021 | Alb (400mg/kg, 1x) | Sch.Child | RCT | 645 | 441 | 6-14 | 11 | 310 | 335 | Kato-Katz. qPCR | 2-3 | 106 | ND | ND | ND | ND | ND | 65.1 | ND | 137 | ND | ND | ND | ND | ND | 98.5 | ND | 90 | ND | ND | ND | ND | ND | 85.6 | ND |
|  | Asia/Lao PDR |  |  |  |  |  |  |  |  |  |  |  |  | 105 | ND | ND | ND | ND | ND | 61 | ND | 111 | ND | ND | ND | ND | ND | 96.5 | ND | 228 | ND | ND | ND | ND | ND | 82 | ND |
|  | Afr/Tanzania |  |  |  |  |  |  |  |  |  |  |  |  | 245 | ND | ND | ND | ND | ND | 29 | ND | 193 | ND | ND | ND | ND | ND | 95.3 | ND | 139 | ND | ND | ND | ND | ND | 63.3 | ND |
| Welsche et al., 2023 | Africa/ Tanzania | 2021 | Alb (400 mg) plus Moxidectin (8 mg) | Sch.Child | RCT | 771 | 550 | 12-19 | 15.8 | 59 | 148 | Kato-Katz | 2-3 | 207 | 144 | 63 | 0 | 450 | 14.3 | 96.8 | 34.3 | 207 | 39 | 61 | 6 | 7819 | 0 | 100 | 100 | 207 | 64 | 0 | 0 | 16 | 125 | 98.8 | 75 |
|  |  |  | Alb (400mg) plus Iver (200 µg/kg) |  |  |  |  |  | 15.8 | 48 | 163 |  |  | 211 | 148 | 63 | 0 | 468 | 4.6 | 99 | 54 | 211 | 49 | 55 | 6 | 5934 | 0.1 | 100 | 96.4 | 211 | 70 | 0 | 0 | 26 | 112 | 97.4 | 62.9 |
|  |  |  | Albendazole (400mg) |  |  |  |  |  | 15.6 | 6 | 13 |  |  | 19 | 14 | 5 | 0 | 421 | 58 | 86.2 | 26.3 | 19 | 4 | 7 | 1 | 8512 | 0.4 | 100 | 91.7 | 19 | 3 | 0 | 0 | 128 | 0 | 100 | 100 |
| Worku, 2018 | Africa, Ethiopia/Jimma | 2017 | Alb (400mg/kg) | Sch.Child | prospective cohor | 393 | 138 | 5-15 | 10 | 182 | 211 | kato-katz | 3 | 88 | 82 | 6 | 0 | 342.1 | 212 | 38 | 42 | 57 | 29 | 25 | 3 | 88977.6 | 12 | 99.9 | 98.2 | 21 | 20 | 1 | 0 | 226.8 | 105.6 | 54.4 | 71.4 |
| Yahia et al ., 2019 | Africa/ Egypt | 2017- 2018 | Alb (400 mg/kg) | Sch.Child | Cross-sectional | 314 | 78 | 2 -13 | ND | 191 | 123 | Kato-Katz, wet mount | 2 | ND | ND | ND | ND | ND | ND | ND | ND | 65 | 57 | 7 | 1 | 4173 | 163 | 96.1 | 93.8 | 17 | 14 | 3 | 0 | 982 | 68 | 91.2 | 88.2 |
| Yap et al., 2013 | Asia/Chaina | 2011-2012 | Alb (400mg/kg)3x | Sch.Child | DBRCT | 250 | 211 | 9-12 |  |  |  | Kato-Katz | 4 | 92 | 82 | 9 | 1 | 216.3 | 24.3 | 88.8 | 19.6 | 94 | 13 | 54 | 27 | 15850 | 1.3 | 99.9 | 91.5 | 60 | 59 | 1 | 0 | 130.4 | 1.2 | 99.1 | 96.7 |
| Zeleke et al., 2020 | Africa/ North west Ethiopia/ | 2018 | Meb (500mg/kg) | Sch.Child | Cross-sectional | 504 | 130 | ND | ND | ND | ND | kato-katz, wet mount | 2-3 | 14 | 14 | 0 | 0 | 149 | 65.1 | 56.3 | 28.6 | 64 | 46 | 17 | 1 | 5186.4 | 2.8 | 99.9 | 96.9 | 52 | 52 | 0 | 0 | 334.4 | 168.5 | 49.6 | 23.1 |
