# Supplementary material for: Efficacy of Albendazole and Mebendazole Against Soil Transmitted Infections among Pre-School and School Age Children: A Systematic Review and Meta-Analysis
Source: J Epidemiol Glob Health. 2024 May 2;14(3):884–904. doi: 10.1007/s44197-024-00231-7 (PMC11442817; doi:10.1007/s44197-024-00231-7)
Supplement: Supplementary file 11 — Supplementary Material 11 [file 44197_2024_231_MOESM11_ESM.docx]

**ROBINS-1 risk of bias assessment tools in seven domains of critical appraisal**

**Domain 1: Risk of bias due to confounding**

**Question (s):** Did the authors control for all the important confounding factors for which this was necessary? Were confounding factors that were controlled for (and for which control was necessary) measured validly and reliably by the variables available in this study? Did the authors control for any variables after the start of the exposure period being studied that could have been affected by the exposure? Did the use of negative controls, or other considerations, suggest serious uncontrolled confounding? Is the risk of bias (due to confounding) sufficiently high, in the context of its likely direction and the magnitude of the estimated exposure effect, to threaten conclusions about whether the exposure has an important effect on the outcome?

**Risk of Bias:**

- **Low risk**: No confounding expected.
- **Moderate risk**: i) Confounding expected, all known important confounding domains appropriately measured and controlled for; and (ii) Reliability and validity of measurement of important domains were sufficient, such that we do not expect serious residual confounding.
- **Serious risk**: (i) At least one known important domain was not appropriately measured, or not controlled for; or (ii) Reliability or validity of measurement of an important domain was low enough that we expect serious residual confounding.
- **Critical risk**: (i) Confounding inherently not controllable or (ii) The use of negative controls strongly suggests unmeasured confounding.

**Domain 2: Risk of bias arising from measurement of the exposure**

Question (s): Does the measured exposure well-characterize the exposure metric specified to be of interest in this study? Was the exposure likely to be measured with error, or misclassified? Is the risk of bias (arising from measurement of exposure) sufficiently high, in the context of its likely direction and the magnitude of the estimated exposure effect, to threaten conclusions about whether the exposure has an important effect on the outcome?

**Risk of Bias:**

- **Low risk:** (i) All participants who would have been eligible for the target trial were included in the study; and (ii) For each participant, start of follow up and start of intervention coincided
- **Moderate risk:** (i) Selection into the study may have been related to intervention and outcome; and The authors used appropriate methods to adjust for the selection bias; or (ii) Start of follow up and start of intervention do not coincide for all participants; and (a) the proportion of participants for which this was the case was too low to induce important bias; or (b) the authors used appropriate methods to adjust for the selection bias; or (c) the review authors are confident that the rate (hazard) ratio for the effect of intervention remains constant over time.
- **Serious risk:** (i) Selection into the study was related (but not very strongly) to intervention and outcome; and This could not be adjusted for in analyses; or (ii) Start of follow up and start of intervention do not coincide; and A potentially important amount of follow-up time is missing from analyses; and The rate ratio is not constant over time.
- **Critical risk:** (i) Selection into the study was very strongly related to intervention and outcome; and this could not be adjusted for in analyses; or (ii) A substantial amount of follow-up time is likely to be missing from analyses;

**Domain 3: Risk of bias in selection of participants into the study (or into the analysis)**

**Question (s):** Did follow-up begin at (or close to) the start of the exposure window for most participants? Is the effect of exposure likely to be constant over the period of follow up analyzed? Were these characteristics likely to be influenced by the outcome or a cause of the outcome?

**Risk of Bias:**

- **Low risk:** i) Intervention status is well defined; and (ii) Intervention definition is based solely on information collected at the time of intervention
- **Moderate risk:** (i) Intervention status is well defined; and (ii) Some aspects of the assignments of intervention status were determined retrospectively
- **Serious risk:** (i) Intervention status is not well defined; or (ii) Major aspects of the assignments of intervention status were determined in a way that could have been affected by knowledge of the outcome.
- **Critical risk:** (Unusual) An extremely high amount of misclassification of intervention status, e.g. because of unusually strong recall biases.

**Domain 4: Risk of bias due to post-exposure interventions**

**Question (s):**  Were there post-exposure interventions that were influenced by prior exposure during the follow-up period? Is it likely that the analysis corrected for the effect of post-exposure interventions that were influenced by prior exposure? What is the predicted direction of bias due to confounding? Is the risk of bias (due post-exposure interventions) sufficiently high, in the context of its likely direction and the magnitude of the estimated exposure effect, to threaten conclusions about whether the exposure has an important effect on the outcome?

**Risk of Bias:**

- **Low risk:** (i) Any deviations from intended intervention reflected usual practice; or (ii) Any deviations from usual practice were unlikely to impact on the outcome.
- **Moderate risk:** (i) there were deviations from intended intervention, but their impact on the outcome is expected to be slight. or (ii) The important co-interventions were not balanced across intervention groups, or there were deviations from the intended interventions (in terms of implementation and/or adherence) that were likely to impact on the outcome; and The analysis was appropriate to estimate the effect of starting and adhering to intervention, allowing for deviations (in terms of implementation, adherence and co-intervention) that were likely to impact on the outcome.
- **Serious risk:** (i) The important co-interventions were not balanced across intervention groups, or there were deviations from the intended interventions (in terms of implementation and/or adherence) that were likely to impact on the outcome; and (ii) The analysis was not appropriate to estimate the effect of starting and adhering to intervention, allowing for deviations (in terms of implementation, adherence and cointervention) that were likely to impact on the outcome.
- **Critical risk:** (i) There were substantial imbalances in important cointerventions across intervention groups, or there were substantial deviations from the intended interventions (in terms of implementation and/or adherence) that were likely to impact on the outcome; and (ii) The analysis was not appropriate to estimate the effect of starting and adhering to intervention, allowing for deviations (in terms of implementation, adherence and cointervention) that were likely to impact on the outcome.

**Domain 5: Risk of bias due to missing data**

**Question (s):** Were complete data on exposure status available for all, or nearly all, participants? Were complete data on the outcome available for all, or nearly all, participants? Were complete data on confounding variables available for all, or nearly all, participants? Was exclusion from the analysis because of missing data (in exposure, confounders or the outcome) likely to be related to the true value of the outcome? Were all or most predictors of missingness (in exposure, confounders or the outcome) included in the analysis model? Was the analysis based on imputing missing values? Was imputation performed appropriately?

**Risk of Bias:**

- **Low risk:** (i) Data were reasonably complete; or (ii) Proportions of and reasons for missing participants were similar across intervention groups; or (iii) The analysis addressed missing data and is likely to have removed any risk of bias.
- **Moderate risk:** (i) Proportions of and reasons for missing participants differ slightly across intervention groups; and (ii) The analysis is unlikely to have removed the risk of bias arising from the missing data
- **Serious risk:** (i) Proportions of missing participants differ substantially across interventions; or Reasons for missing differ substantially across interventions; and (ii) The analysis is unlikely to have removed the risk of bias arising from the missing data; or Missing data were addressed inappropriately in the analysis; or The nature of the missing data means that the risk of bias cannot be removed through appropriate analysis.
- **Critical risk:** (i) (Unusual) There were critical differences between interventions in participants with missing data; and (ii) Missing data were not, or could not, be addressed through appropriate analysis.

**Domain 6: Risk of bias arising from measurement of the outcome**

**Question (s):** Could measurement or ascertainment of the outcome have differed between exposure groups or levels of exposure? Were outcome assessors aware of study participants’ exposure history? Could assessment of the outcome have been influenced by knowledge of participants’ exposure history? What is the predicted direction of bias arising from measurement of outcomes? Is the risk of bias (arising from measurement of outcomes) sufficiently high, in the context of its likely direction and the magnitude of the estimated exposure effect, to threaten conclusions about whether the exposure has an important effect on the outcome?

**Risk of Bias:**

- **Low risk:** (i) The methods of outcome assessment were comparable across intervention groups; and (ii) The outcome measure was unlikely to be influenced by knowledge of the intervention received by study participants (i.e. is objective) or the outcome assessors were unaware of the intervention received by study participants; and (iii) Any error in measuring the outcome is unrelated to intervention status.
- **Moderate risk:** (i) The methods of outcome assessment were comparable across intervention groups; and (ii) The outcome measure is only minimally influenced by knowledge of the intervention received by study participants; and (iii) Any error in measuring the outcome is only minimally related to intervention status.
- **Serious risk:** (i) The methods of outcome assessment were not comparable across intervention groups; or (ii) The outcome measure was subjective (i.e. vulnerable to influence by knowledge of the intervention received by study participants); and The outcome was assessed by assessors aware of the intervention received by study participants; or (iii) Error in measuring the outcome was related to intervention status.
- **Critical risk:** The methods of outcome assessment were so different that they cannot reasonably be compared across intervention groups.

**Domain 7: Risk of bias in selection of the reported result**

**Question (s):** Was the result reported in accordance with an available, pre-determined analysis plan? Is the reported effect estimate likely to be selected, based on desirability of the magnitude (or statistical significance) of the estimated effect of exposure on outcome, from multiple *exposure measurements* within the exposure domain? Is the reported effect estimate likely to be selected, based on desirability of the magnitude (or statistical significance) of the estimated effect of exposure on outcome, from multiple *outcome* *measurements* within the outcome domain? Is the reported effect estimate likely to be selected, based on desirability of the magnitude (or statistical significance) of the estimated effect of exposure on outcome, from multiple *analyses* of the exposure-outcome relationship? What is the predicted direction of bias due to selection of the reported result? Is the risk of bias (due to selection of the reported result) sufficiently high, in the context of its likely direction and the magnitude of the estimated exposure effect, to threaten conclusions about whether the exposure has an important effect on the outcome?

**Risk of Bias:**

- **Low risk**: There is clear evidence (usually through examination of a pre-registered protocol or statistical analysis plan) that all reported results correspond to all intended outcomes, analyses and subcohorts.
- **Moderate risk**: (i) The outcome measurements and analyses are consistent with an a priori plan; or are clearly defined and both internally and externally consistent; and (ii) There is no indication of selection of the reported analysis from among multiple analyses; and (iii) There is no indication of selection of the cohort or subgroups for analysis and reporting on the basis of the results
- **Serious risk**: (i) Outcomes are defined in different ways in the methods and results sections, or in different publications of the study; or (ii) There is a high risk of selective reporting from among multiple analyses; or (iii) The cohort or subgroup is selected from a larger study for analysis and appears to be reported on the basis of the results
- **Critical risk**: (i) There is evidence or strong suspicion of selective reporting of results; and (ii) The unreported results are likely to be substantially different from the reported results.

***RoB-1 Risk of bias assessment tool***

- **Domain 1. Adequate sequence generation?**
  - Question: Was the allocation sequence adequately generated?
  - Bias: random sequence generation (selection bias)
  - Explanation: selection bias (biased allocation to intervention) due to inadequate generation of a randomized sequence

Implementation of this criterion for the evaluation of the studies using the first domain of the critical appraisal tool was:

- If randomization was carried out with an appropriate method, such as a computer-assisted or lottery method, the study was classified as having a “low” risk of a bias; if randomization was not done, the study was classified as having a “high” risk of a bias; and if insufficient information regarding the implementation process was available, the study could not be classified as having a high or low risk of a bias and was defined instead as having an “unclear” risk of a bias.
- **Domain 2. Allocation concealment?**
  - Question: Was allocation adequately concealed?
  - Bias: Allocation concealment (selection bias)
  - Explanation: selection bias (biased allocation to intervention) due to inadequate concealment of allocations prior to assignment.

If selection of the participants is blinded to the data collectors, or through use of sealed postal allocation, it was classified as having a ‘*low*’ risk of bias; or if selection of participants was not blinded to the data collector or the participants were selected based on specific criteria, it was classified as a ‘*high’* risk of bias; or if the information provided is not clear or insufficient to decide, it was classified as an ‘*unclear*’ risk of bias.

- **Domain 3. Blinding?**
- Question: Was knowledge of the allocated interventions adequately prevented?
- Bias: binding (performance bias and detection bias)
- Explanation: performance bias or detection bias due to knowledge of the allocated interventions after assignment

This performance or detection bias was evaluated for each study using a method. If the statistical analysis were blindly conducted by a person who is unaware of the population or some proportion of the findings (slides) was randomly evaluated by a person blind to the intervention or study group, it was classified as a ‘low’ risk factor; if the analysis or outcome evaluation was not conducted blindly, it was classified as a risk of bias; if information about such blinding was missing in the study, it was classified as an ‘unclear’ risk of bias.

- **Domain 4.** Is incomplete outcome data addressed?
  - Question: Were incomplete outcome data adequately addressed?
  - Bias: incomplete outcome data (attrition bias)
  - Explanation: attrition bias due to the amount, nature, or handling of an incomplete outcome

The classification of the risk of bias was based on the number of participants who missed or lost a follow-up or refused to continue the follow-up. If the missed participants were very small or none, it was classified as a ’low’ risk of bias; if a substantial number of the study participants were missed or lost (e.g., >15%) before final data collection, it was classified as a ‘high’ risk of bias; if there is no information about the attrition rate of the participants, the risk of bias is classified as 'unclear'.

- **Domain 5. Free of selective reporting?**
  - Question: Are reports of the study free of suggestions of selective outcome reporting?
  - Bias: selective reporting (reporting bias)
  - Explanation

Bias for selective reporting was assessed objectively based on the expected primary findings targeted in the study protocol, which were egg reduction rate (ERR) and cure rate (CR). Studies with clear reporting of these findings along with supplementary results were classified as ‘’ risk factors, while studies that missed this information or wrongly presented the findings were classified as ‘high’ risk factors. A study with unclear information about the primary findings was classified as having an ‘unclear’ risk of bias.

**Domain 6** **Free of other biases?**

- - Question: Was the study apparently free of other problems that could put it at high risk of bias?
  - Bias: other bias
  - Explanation: bias due to problems not covered in the table

This appraisal domain was assessed to see if there are biases that are not covered by the six criteria.
